# Equity in antenatal care visits among adolescent mothers: An analysis of 54 country levels trend and projection of coverage from 2000 to 2030

**DOI:** 10.7189/jogh.12.04016

**Published:** 2022-03-19

**Authors:** Mizanur Rahman, Fahima Hossain, Rashedul Islam, Jenny Jung, Syed Riaz Mahmud, Masahiro Hashizume

**Affiliations:** 1 Hitotsubashi Institute for Advanced Study, University of Hitotsubashi, Tokyo, Japan; 2 Department of Global Health Policy, School of International Health, The University of Tokyo, Tokyo, Japan; 3 Global Public Health Research Foundation, Dhaka, Bangladesh; 4 Maternal, Child and Adolescent Health Program, Burnet Institute, Melbourne, Australia

## Abstract

**Background:**

Ensuring utilization of antenatal care (ANC) services by adolescent mothers (ages 10-19) is an enormous challenge in low-and middle-income countries (LMICs). This study provides the first comprehensive analysis of ANC visits among adolescent and adult mothers.

**Methods:**

Using all available Demographic and Health Survey and Multiple Indicator Cluster Surveys between 2000 and 2019 in 54 LMICs, we estimated proportion of ANC visits among women. Bayesian hierarchical regression models were used to estimate trend, projection, and determinants of single and four ANC visits (ANC1 and ANC4) independently. Equity analysis were performed to assess the magnitude of wealth-based and urban-rural inequalities in access to ANC visits.

**Results:**

Compared to women aged 36-49 years, coverage of ANC1 and ANC4 are expected to increase significantly for adolescent mothers and women aged 20-35 years. This increase was observed at the national level, as well as both urban and rural areas in most countries between 2000 and 2030. By 2030, the coverage of ANC1 is predicted to reach 80% or more in all countries except Angola, Central African Republic and Togo, whereas only 16 countries are predicted to reach 80% or more for ANC4. According to wealth quintile, the lowest inequalities with highest coverage of 80% or more ANC4 will be observed in Armenia, Cambodia, Dominican Republic, Ghana, Maldives, Indonesia, and Sao Tome and Principe in 2030. Determinant analysis found increased odds of receiving ANC visits during pregnancy for adolescent mothers with higher educational levels, frequency of listening/watching mass media, and various household socio-economic status factors.

**Conclusions:**

This study calls for advanced, innovative and cost-effective approaches to increase ANC coverage among adolescent mothers, particularly in rural areas and/or in low socioeconomic groups.

Pregnancy among adolescents contributes to 11% of global births (16 million births among girls aged 15-19 years annually), of which 95% occur in low- and middle-income countries (LMICs) [1,2]. Adolescent pregnancy is a global concern due to its detrimental consequences on health and socio-economic aspects. An estimated 10% of global annual maternal deaths occur in adolescents, and maternal deaths are ranked as the second leading cause of death in this age group worldwide [2]. Thus, reducing the health and societal impacts of adolescent pregnancy is critical due to the higher risk of adverse maternal and infant outcomes faced such as maternal mortality, maternal anemia, gestational hypertension, malnutrition, eclampsia, neonatal mortality, preterm birth, and low birth weight [3,4].

Antenatal care (ANC) services provide a critical opportunity to manage and prevent adverse outcomes in adolescent mothers. Starting with ANC, receiving a continuum of care from early pregnancy to the postpartum period is vital in providing timely application of effective interventions to improve maternal and newborn health outcomes. It is widely established that the number of ANC visits and consequent institutional delivery, postnatal care visits, and childhood immunization are inversely associated with the risks of maternal and newborn mortalities and morbidities [5-8]. Yet, adolescent health care services remain often poorly coordinated in both high-income and LMICs [9-11]. Particularly for adolescent mothers, effective coordination highly influences care seeking behavior by three service-related factors: availability, acceptability, and affordability. Moreover, adolescent mothers often face additional barriers to utilization of ANC services such as biological immaturity, financial constraints, cultural beliefs, reduced familial support, lack of health education, and reduced decision-making autonomy. Despite these vulnerabilities [12,13], the lack of focus on adolescent mothers during the Millennium Development Goal (MDG) era drew attention to the propagated barriers to ANC visits and efforts to address this “left behind” population were prioritized in the post-2015 agenda [14]. Furthermore, the need to address inequity is increasingly highlighted by previous research which demonstrated gaps and inadequate ANC and skilled birth attendant (SBA) utilization and hence higher rates of maternal deaths among the poor, rural and less-educated women [15,16].

In order to develop and implement adolescent-specific interventions to reduce inequity, a comprehensive understanding of the level and trends of ANC utilization and their determinants is essential. For this purpose, an important metric for policy makers is the country-specific estimates of ANC utilization measured by at least one visit and four visits during pregnancy. However, majority of current literature explore these estimates focusing on adult pregnant women [12,13,17-20], while the systematic examination of the variations in access to ANC services among adolescent mothers remains rare. Therefore, this study focused on adolescent mothers as the primary population of interest, and also on adult mothers categorized into two age groups (20-35 and 36-49 years) as secondary target populations. For this purpose, a priori hypothesis was generated that the trend in single and four ANC visits of reproductive age group of women increased over time in LMICs and that it was influenced by individual-, household-, and country-specific socio-economic factors. The objective of this study was to estimate the recent trends in access to ANC services and to develop projections up to 2030 for 54 LMICs at the national level, as well as by socio-economic status and area of residence. This study further assessed the determinants and magnitude of wealth-based inequality in access to health services, and provides comparative findings for women of different age groups.

## METHODS

### Data sources

This study was a secondary data analysis using relevant data from all Demographic and Health Surveys (DHS) and Multiple Indicator Cluster Surveys (MICS) carried out in LMICs since 2000. Both surveys were cross-sectional in nature and provided nationally representative sample with high response rate. DHS and MICS followed similar standardized methods and questionnaires to collect information on a wide range of key demographic and health issues relevant to LMICs. The detailed sources of individual level data are available at https://dhsprogram.com/Data/ and https://mics.unicef.org/surveys. The study included 54 countries and 224 survey data points from 2000 to 2019. The DHS and MICS followed cross-sectional cluster survey methods to select households and collect information of all household members. The primary population of interest was adolescent mothers, and the secondary target population was adult mothers categorized into two age groups (20-35 and 36-49 years). Participants with most recent pregnancy or birth were included in this study. The details of the included 54 countries with brief survey characteristics are presented in the supplemental appendix (Figure S1 and Table S1 in the **Online Supplementary Document**).

### Outcome variables

Since ANC was proven to be a potential determinant of maternal and child health outcome, it was selected as the outcome variable. Consistent with previous studies [17], coverage of single ANC visit (ANC1) was defined as the proportion of women with recent pregnancy or birth who received at least one ANC visit from skilled health professional such as doctor, nurse, midwife and other country-specific health cadres. Similarly, coverage of four ANC visits (ANC4) refers to the proportion of women who received at least four ANC visits from skilled health professionals. Since DHS, MICS, or other online macro databases didn’t present proportion of ANC visits which are provided only by skilled providers directly, country-specific individual level DHS and MICS data sets were used to estimate proportion of ANC1 and ANC4 from skilled health professional at national, urban-rural and different wealth-quintile levels. All the proportion estimates were considered for probability sampling weights.

### Predictor variables

Country and year specific socio-demographic index (SDI) and Human Resource for Health (HRH) were used to estimate trend and projection of ANC visits for each country. Availability, comparability, and consistency with previous literature [12,13,18,19,21-25], were considered for a range of individual, household and community level independent variables such as age of household head (<30 years, 30-45 years, 46-60 years, and >60 years), sex of the household head (male or female), adolescent mother’s level of education (no education, primary, secondary, and higher education), parity (1, 2, and 3 or more), access to mass media (none, listen or watch once a week, and listen or watch at least once a week), household wealth quintile (poorest, poorer, average, rich, and richest), and area of residence (urban or rural). Wealth index, an asset-based measure of household wealth, is widely used as a measure of socio-economic status for household in LMICs [26]. Generally, principal component analysis is used to calculate a wealth score, which represents the position of a household in the wealth distribution relative to the rest of the households of that country [27,28]. The sample is divided into quintiles to represent a relative measure of household wealth, where the lowest 20% values of the wealth score presents the bottom 20% household of the wealth distribution (ie, poorest) and the highest 20% values presents the richest households. The areas of residence of a household were classified by the survey authorities either as rural or urban based on geographical boundaries defined by the national authorities in each of the 54 countries during the survey period.

### Analysis

The primary outcomes were the proportion of ANC1 and ANC4 among women aged 15-19, 20-35, and 36-49 years. We fitted Bayesian hierarchical regressions to estimate coverage of ANC visits over time, by age group, area of residence, wealth quintile and country, with two models – one for prevalence of ANC1 and another for ANC4. Using fully Bayesian statistical inference [11], our posterior estimates of ANC visits variables were able to flexibly borrow strength such that country-specific estimates were informed by study data from the same country, where available, and by study data from other countries in the same region or the same year. Non-informative priori was employed in this analysis. The following Bayesian hierarchical regression model was used to estimate the trend in, and projection of health service indicators up to 2030 at country level:

*y_ijkl_* ~ *Normal*(*ŷ_jkl_*,*τ^2^*)

*ŷ_jkl_* = *β_0,jk_*+*β_1,jk_year_jk_*+*β_2,jk_SDI_jk_*+*β_3,jkl_HRH_jk_*

*β_jk_* ~ *N*(*β_j_*,*σ_j_^2^*)

*β_j_* ~ *N*(β,*σ^2^*)

β ~ *Normal*(0,10000)

σ_j_, σ ~ *Gamma*(0.001,0.001)

τ ~ *Uniform*(0,1000)

where y is the logit-transformed probability of ANC visit prevalence from year *l* in country k and region j. SDI and HRH were the year-specific (*l*) predictor variables from country k in region j. *β_jk_* = [*β_0,jk_*, *β_1,jk_*, *β_2,jk_*, *β_3,jk_*] is the country specific linear regression model parameter vector which includes intercept (*β_0,jk_*) and the slopes for year (*β_1,jk_*), SDI (*β_2,jk_*), and HRH (*β_3,jk_*). *τ^2^* is the model error variance, *β_j_* = [*β_0,j_*, *β_1,j_*, *β_2,j_*, *β_3,j_*] is the vector of model parameter mean for country region j. σ*_j_*^2^ = [*σ_0,j_^2^, σ_1,j_^2^*, *σ_2,j_^2^*, *σ_3,j_^2^*] is the vector of variance of model parameters among countries belongings to region j, while β = [*β_0_*, *β_1_*, β, *β_3_*] and σ*_j_*^2^ = [*σ_0_^2^, σ_1_^2^*, *σ_2_^2^*, *σ_3_^2^*] are the means and variance among regions, respectively. Non-informative prior distributions were assigned to β, τ, *σ_j_*, and σ, which represent the hyperparameters and follow a normal distribution with mean 0 and variance 10 000; a uniform distribution with lower (0) and upper (1000) limits; and a gamma distribution with shape parameter k (0.001) and scale parameter (θ), (0.0001), respectively. The hyperparameters of τ, *σ_j_*, and σ, are considered non-informative as there is no information about their distribution.

The quintile model was developed based on the quintiles specific proportion of ANC visits with covariates from the national model. An interaction term was added in the quintiles model nesting the same quintiles within the country. We fitted our ANC models using Bayesian inference, sampling from the posterior distribution over the parameters using Gibbs Monte Carlo, a Markov chain Monte Carlo (MCMC) method, as implemented in the algorithm in Just Another Gibbs Sampler (JAGS) software (version 4.2). In MCMC algorithm, we used 10 000 iterations with three chain, 10 thinning, and 2000 sample burn-in. Only countries with at least one data point were included in this study. 95% credible interval (CrI) were constructed from the 2.5 and 97.5 percentiles of the posterior samples. For model diagnostic, trace plot and Gelman-Rubin diagnostic statistics were used to check the convergence status. Both covariates and the model’s hierarchical structure influence how data from other countries influence predictions for a given country. We examined the sensitivity of our results by two approaches: (1) the exclusion of country-level covariates (SDI and HRH), and (2) altering priors for the hyperparameters. The detailed information of the model parameters and sensitivity analysis is presented in the supplemental appendix (e-method 1 in the **Online Supplementary Document**).

Finally, the magnitude of wealth-based inequality in access to ANC services was calculated for each year using slope index of inequality (SII). The SII values range between -100 to +100 and a value equal to 0 indicates no inequality. Country-specific rate of change in coverage of ANC visits between 2000 and 2030 was estimated from the predicted values. Descriptive analysis and rate of change were calculated using Stata 16.1 version (StataCorp, College Station, TX USA).

## RESULTS

### Survey characteristics

In total, data from 224 nationally representative household survey data conducted between 2000 and 2019 were included in this study, representing 54 LMICs. The country-specific survey data points with survey types are presented in the appendix (Figure S1 in the **Online Supplementary Document**).

### National coverage of ANC visits

**Table 1** shows country-specific predicted coverage of ANC visits among adolescent mothers at national level in 2000, 2018, and 2030. Compared to women aged 20-35 years, the coverage of ANC visits among adolescent mothers is relatively low, however higher when compared to women aged 36-49 years (Table S3-S4 in the **Online Supplementary Document**). Overall, the coverage of ANC4 was much lower than ANC1 in all age groups and across all countries. For adolescent mothers, the rate of increase in ANC4 was higher than ANC1 in most countries between 2000 and 2030 (**Table 1**). The predicted coverage of ANC1 is expected to reach around 80% or more in 52 of 54 countries except Angola, Central African Republic and Togo by 2030 (**Table 1** and Figure S2 in the **Online Supplementary Document**). However, the predicted coverage of ANC4 reaching 80% or greater by 2030 was observed in only 16 of 54 countries: Maldives, Nepal, Cambodia, Indonesia, Philippines, Timor-Leste, Rwanda, Congo, Ghana, Liberia, Sao Tome and Principe, the Gambia, Dominican Republic, Honduras, Armenia, and Kyrgyzstan (**Table 1** and Figure S3 in the **Online Supplementary Document**). Several countries such as Bangladesh, Malawi, Mozambique, Tanzania, Zambia, Central African Republic, Guinea, and Nigeria, are predicted to have around 50% or less coverage of ANC4 in 2030.

**Table 1:** National level predicted coverage of antenatal care visits among adolescent mothers aged 15-19 years in LMICs, 2000-2030

Country	ANC1 (proportion, 95% CrI)			ANC4 (proportion, 95% CrI)		
**2000**	**2018**	**2030**	**2000**	**2018**	**2030**
Afghanistan	11.0 (3.7-24.5)	71.5 (53.2-84.8)	94.2 (78.7-99.2)	4.1 (1.0-10.1)	21.7 (13.7-32.6)	52.6 (21-84.8)
Bangladesh	45.2 (26.7-63.7)	68.6 (52.3-81.9)	79.1 (47.3-95.3)	15.3 (9.8-22.2)	27.4 (19.9-36.5)	39.1 (20.6-60.9)
India	71.1 (44.2-90.3)	82.7 (66.7-92.6)	85.7 (53.0-98.1)	29.3 (17.3-44.4)	55.1 (42.4-67.4)	71.2 (50.4-87.0)
Maldives	99.9 (99.7-100.0)	99.3 (98.4-99.7)	95.9 (84.6-99.7)	88.2 (76.0-95.0)	87.7 (81.7-92.2)	86.2 (68.3-95.4)
Nepal	36.1 (21.1-53.8)	84.1 (74.4-91.2)	95.6 (88.2-98.8)	15.7 (11.1-22.0)	62.2 (53.0-70.7)	87.6 (78.8-93.6)
Pakistan	46.2 (16.5-79.0)	85.1 (73.4-92.6)	94.5 (82.7-99.0)	19.5 (8.8-36.1)	44.3 (33.8-55.0)	64.2 (44.2-81.7)
Cambodia	45.0 (28.7-61.9)	98.7 (97.4-99.5)	99.9 (99.7-100.0)	14.3 (9.9-19.4)	85.7 (79.6-90.4)	98.4 (96.8-99.4)
Indonesia	86.7 (75.4-93.7)	94.8 (89.9-97.5)	96.9 (90.3-99.3)	68.6 (57.5-78.5)	83.7 (77.7-88.2)	89.8 (81.2-95.2)
Laos	23.1 (12.1-36.8)	75.7 (61.4-87.1)	93.2 (82.6-98.3)	12.7 (3.9-31.4)	49.3 (37.5-61.6)	77.5 (51.1-93.5)
Myanmar	75.3 (31.2-97.0)	83.6 (67.9-93.3)	82.6 (41.1-98.7)	31.6 (5.6-70.1)	49.9 (35.8-63.8)	62.9 (24.8-90.9)
Papua New Guinea	55.8 (3.2-98.5)	80.9 (64.7-91.5)	84.3 (37.0-99.3)	37.5 (6.3-79.7)	50.9 (38.9-62.5)	60.7 (30.0-88.1)
Philippines	86.4 (74.6-93.9)	93.9 (88.8-97.1)	96.0 (88.7-99.1)	63.5 (51.6-73.9)	80.5 (73.7-86.0)	87.7 (78.2-94.4)
Timor-Leste	71.8 (32.1-95.2)	86.2 (72.5-94.7)	88.0 (54.7-99.2)	26.1 (10.3-48.2)	76.0 (64.1-84.8)	92.8 (81.1-98.4)
Vietnam	68.3 (54.0-80.7)	91.2 (83.7-95.9)	96.3 (89.4-99.2)	23.7 (15-33.4)	53.0 (40.4-64.9)	72.4 (50.8-87.4)
Angola	93.5 (83.7-98.1)	67.7 (48.6-83.0)	37.9 (7.5-79.0)	33.5 (17.2-53.4)	50.2 (37.9-61.6)	62.2 (37.2-82.9)
Burundi	83.1 (72.9-90.2)	99.2 (98.7-99.6)	99.9 (99.8-100.0)	23.1 (9.6-41.9)	52.2 (41.5-63.1)	72.1 (48.3-89.6)
Comoros	74.0 (58.4-86.0)	93.1 (84.2-97.7)	96.9 (88.1-99.6)	28.2 (8.4-59.3)	49.8 (31.8-68.6)	64.3 (22.7-93.1)
Ethiopia	20.3 (11.0-33.3)	72.8 (57.0-84.8)	92.1 (78.0-98.1)	8.9 (5.9-12.3)	30.0 (22-38.9)	53.6 (35.2-70.8)
Kenya	75.1 (62.5-85.0)	95.3 (91.3-97.8)	98.5 (95.8-99.7)	38.8 (27.8-51.1)	53.2 (42.6-63.7)	64.5 (44.0-81.3)
Lesotho	84.1 (66.5-94.3)	97.0 (92.9-98.9)	98.7 (94.2-99.9)	67.2 (53.9-78.4)	65.6 (54.1-76.1)	64.7 (41.4-84.0)
Madagascar	66.5 (52.9-78.9)	86.8 (78.6-92.7)	93.3 (83.1-97.9)	29.0 (16.6-44.2)	56.3 (35.1-76.6)	72.3 (35.1-94.3)
Malawi	90.5 (84.5-94.6)	97.9 (96.6-98.8)	99.2 (98.3-99.7)	55.2 (46.2-63.0)	40.3 (32.3-49.1)	31.9 (20.1-47.0)
Mozambique	89.8 (81.4-95.3)	92.0 (86.2-95.7)	92.5 (81.3-97.8)	52.2 (40.1-65.5)	49.5 (40.7-58.1)	48.2 (32.3-64.0)
Rwanda	82.8 (71.8-90.6)	99.6 (99.2-99.8)	100.0 (99.9-100.0)	8.8 (6.0-12.5)	51.6 (41.2-62.1)	84.7 (73.4-92.7)
Tanzania	91.4 (81.3-96.8)	97.3 (95.1-98.7)	98.5 (95.8-99.7)	55.6 (42.2-68.7)	48.8 (39.1-58.3)	44.8 (27.5-63.9)
Uganda	92.8 (86.9-96.4)	95.7 (92.6-97.7)	96.6 (91.1-99.1)	41.5 (31.7-52.2)	57.0 (46.2-67.4)	66.8 (48.8-81.7)
Zambia	91.3 (84.1-95.9)	96.5 (93.8-98.2)	97.9 (94.7-99.5)	61.8 (50.4-71.5)	53.0 (42.7-62.1)	47.4 (30.3-63.6)
Zimbabwe	87.6 (73.5-96.0)	92.1 (85.7-96.5)	92.8 (75.7-99.0)	54.5 (39.7-68.1)	68.3 (59.1-76.3)	76.3 (57.1-88.9)
Benin	75.9 (54.9-90.1)	82.2 (72.2-89.5)	84.7 (59.9-96.1)	57.6 (43.4-71.0)	47.9 (39.1-56.0)	44.6 (26.1-62.6)
Burkina Faso	74.4 (56.4-87.5)	97.9 (94.0-99.5)	99.5 (97.0-100.0)	15.1 (9.0-22.7)	48.9 (32.7-66.9)	73.3 (46.0-92.3)
Cameroon	69.6 (56.0-81.1)	88.9 (78.9-94.7)	94.3 (82.7-98.7)	54.5 (40.2-68.2)	52.4 (41.0-63.5)	51.7 (29.7-73.6)
Central African Republic	61.7 (45.8-76.6)	74.0 (50.7-90.7)	78.5 (38.7-97.5)	26.5 (18.5-35.9)	33.7 (19.5-48.6)	40.0 (16.1-67.1)
Chad	37.6 (24.3-52.2)	70.3 (54.3-84.2)	84.4 (61.4-96.1)	11.1 (6.8-16.9)	37.8 (26.9-48.9)	63.2 (40.2-82.1)
Congo	78.7 (57.6-91.9)	93.3 (86.7-97.1)	96.3 (85.9-99.5)	65.6 (51.5-78.1)	76.3 (67.2-84.0)	81.9 (65.4-92.9)
Cote d'Ivoire	79.1 (56.0-92.8)	93.6 (86.8-97.4)	96.5 (87.1-99.6)	32.3 (6.5-72.2)	49.5 (37.4-62.3)	62.3 (25.8-89.6)
Democratic Republic of the Congo	79.2 (65.7-88.7)	85.6 (77.0-91.8)	88.3 (73.2-96.2)	36.8 (24.2-51.3)	47.2 (38.2-56.4)	55.8 (37.0-73.2)
Ghana	91.2 (83.4-96.0)	98.1 (96.1-99.2)	99.2 (97.1-99.9)	60.1 (49.1-70.3)	80.5 (72.3-87.2)	88.6 (75.9-95.6)
Guinea	84.7 (68.6-94.2)	86.1 (77.5-92.0)	85.3 (65.7-96.0)	55.6 (41.0-68.3)	43.0 (34.4-52.1)	35.6 (20.9-52.1)
Liberia	47.8 (19.3-77.1)	98.6 (97.0-99.4)	99.9 (99.6-100.0)	44.5 (28.0-64.4)	79.5 (71.1-86.8)	91.4 (81.5-97.1)
Mali	56.9 (38.4-74.1)	82.5 (73.1-89.3)	91.2 (80.2-97.2)	27.9 (20.1-37.1)	41.4 (33.1-49.7)	52.5 (37.0-66.2)
Niger	37.9 (21.7-55.0)	89.5 (77.3-96.1)	97.6 (91.0-99.7)	8.9 (4.1-16.0)	41.2 (25.7-57.9)	71.2 (40.2-92.0)
Nigeria	43.3 (27.9-60.3)	57.0 (43.2-69.1)	65.3 (40.3-84.3)	32.3 (23.8-41.6)	39.3 (32.2-46.8)	44.9 (31.5-59.1)
Sao Tome and Principe	80.0 (66.2-90.4)	100.0 (99.9-100)	100.0 (100.0-100.0)	60.1 (35.4-80.2)	82.4 (72.8-90.1)	89.6 (71.9-97.9)
Senegal	78.4 (67.2-87.8)	96.6 (94.6-97.9)	99.0 (97.8-99.7)	32.0 (20.9-44.1)	50.3 (42.8-57.9)	63.0 (47.4-77.6)
Sierra Leone	64.9 (49.0-77.5)	98.5 (97.3-99.2)	99.8 (99.6-100.0)	45.7 (28.9-62.5)	78.1 (69.9-84.9)	89.9 (78.6-96.2)
The Gambia	96.1 (90.3-98.8)	99.0 (98.0-99.5)	99.5 (98.4-99.9)	59.6 (45.3-72.9)	74.2 (66.2-80.6)	81.5 (67.5-90.9)
Togo	81.3 (63.3-92.8)	66.1 (49.9-80.2)	53.0 (22.2-83.2)	25.0 (10.0-50.1)	46.8 (32.2-61.6)	62.7 (27.8-88.4)
Dominican Republic	64.7 (30.1-90.3)	99.8 (99.5-100.0)	100.0 (100.0-100.0)	82.3 (67.6-92.2)	94.0 (89.2-97.2)	96.9 (89.9-99.5)
Haiti	78.6 (55.4-92.7)	91.9 (85.0-96.3)	95.1 (82.3-99.3)	42.5 (28.2-59.4)	59.0 (48.5-69.6)	70.1 (49.0-86.3)
Honduras	82.7 (57.6-95.4)	97.6 (93.1-99.4)	98.9 (93.1-100.0)	83.9 (70.6-91.9)	87.0 (77.9-93.4)	87.4 (66.8-97.4)
**Central and Eastern Europe:**						
Albania	96.7 (89.5-99.4)	91.6 (85.1-95.7)	81.4 (48.7-96.5)	72.2 (56.0-85.8)	75.7 (67.9-82.5)	77.9 (60.6-90.0)
Armenia	85.6 (70.2-94.8)	99.9 (99.9-100.0)	100.0 (100.0-100.0)	68.1 (54.7-78.9)	94.5 (91.7-96.4)	98.6 (96.7-99.5)
Kyrgyzstan	85.4 (67.8-95.3)	99.5 (99.0-99.7)	99.9 (99.8-100.0)	69.1 (45.5-87.2)	90.6 (86.5-93.6)	95.9 (89.8-98.8)
Tajikistan	75.6 (62.5-86.7)	92.4 (86.2-96.5)	96.5 (90.7-99.2)	47.5 (22.1-73.7)	68.2 (57.7-78.3)	78.7 (56.3-93.3)

ANC1 – single antenatal visit, ANC4 – at least four antenatal visits, CrI – credible intervals, LMIC – low- and middle-income countries

### Urban-rural disparities in coverage of ANC visits

**Figure 1:**
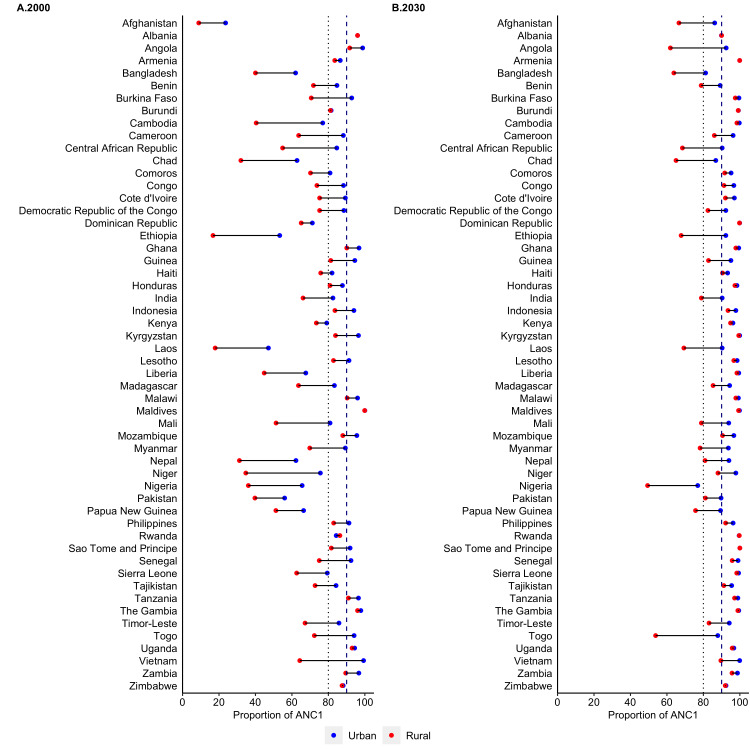
Coverage of at least single antenatal care visit among adolescent mothers aged 15-19 according to area of residence in 54 LMICs, 2000-2030. LMICs – low- and middle-income countries

**Figure 1** presents the urban-rural specific predicted coverage of ANC1 for all 54 included countries in 2000 and 2030. The observed predicted coverage of ANC1 by area of residence is presented in **Figure 1** and Table S4 in the **Online Supplementary Document**. Similar to national coverage trend, the predicted coverage of single visits both at urban and rural areas increased substantially from 2000 to 2030 in all countries. Around 50% or more countries already reached ANC1 coverage by 80% or more in 2000 at urban areas and all countries are predicted to reach the target by 2030 except Angola, Albania and Togo. Similarly, in rural areas, the coverage of ANC1 is expected to cross 80% by 2030 by most countries except Albania, Angola, Bangladesh, Central African Republic, Myanmar, Nigeria, and Togo. Although the coverage of ANC1 is increasing rapidly, a wide urban-rural disparity was observed in several countries including Angola, Central African Republic, Myanmar, Nigeria, and Togo. The lowest urban-rural coverage gap was noticed in Albania, Armenia, Burkina Faso, Burundi, Cambodia, Dominican Republic, Ghana, Kenya, Kyrgyzstan, Liberia, Malawi, Rwanda, Sao Tome and Principe, Sierra Leone, The Gambia, and Zimbabwe.

**Figure 2:**
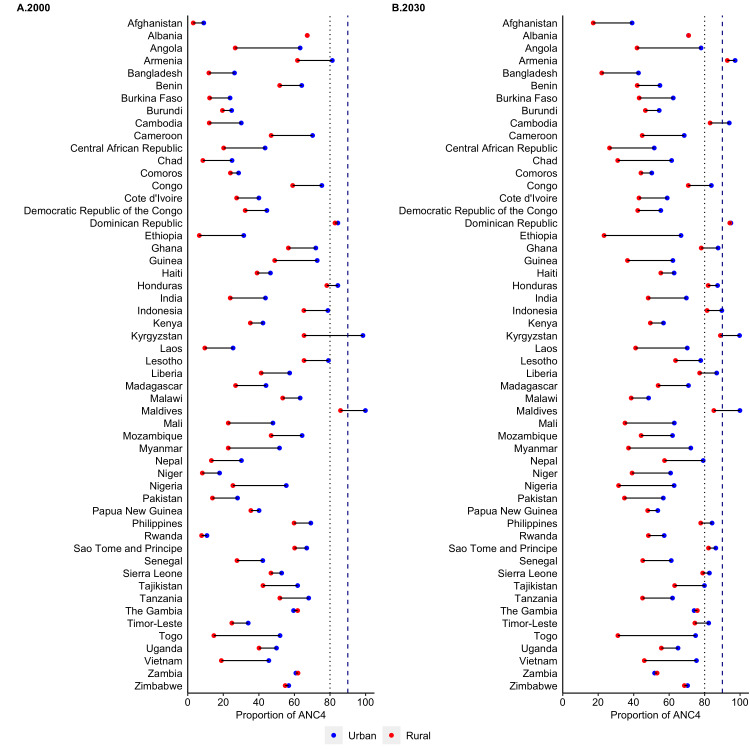
Coverage of at least four antenatal care visits among adolescent mothers aged 15-19 according to area of residence in 54 LMICs, 2000-2030. LMICs – low- and middle-income countries

The predicted coverage of ANC4 in 2000 and 2030 by area of residence across 54 LMICs are presented in **Figure 2** and Table S5 in the **Online Supplementary Document**. Of the 54 countries, only Dominican Republic and Maldives reached 80% coverage in 2000 in both urban and rural residences, and are predicted to maintain this coverage up to the year 2030. Although, the coverage of ANC4 increased at a faster rate than ANC1 in urban and rural areas (Table S6 in the **Online Supplementary Document**), only 16 countries are predicted to reach 80% or more in both urban and rural regions by 2030: Armenia, Cambodia, Dominican Republic, The Gambia, Ghana, Honduras, Indonesia, Kyrgyzstan, Liberia, Maldives, Nepal, Philippines, Rwanda, Timor-Leste, Sao Tome and Principe, Sierra Leone. Similar to ANC1, the access to ANC4 in urban areas was substantially higher than those in rural areas and only the urban areas in 13 countries are predicted to reach 80% or more in 2030. Most of the urban areas in countries had coverage rates ranging from 60% to 80% in 2030, and most of the rural areas’ coverage rates ranged from 35% to 75%. Relatively low urban-rural disparities for ANC4 coverage were observed in six countries such as Albania, Armenia, Cambodia, Dominican Republic, The Gambia and Zimbabwe. A wide urban-rural disparity in coverage rates of ANC4 is predicted to be observed in Afghanistan, Angola, Bangladesh, Benin, Burkina Faso, Cameroon, Central African Republic, Chad, Ethiopia, Guinea, India, Mali, Mozambique, Myanmar, Nigeria, Pakistan, Togo, and Vietnam.

### Wealth-based inequity in coverage of ANC visits

**Figure 3:**
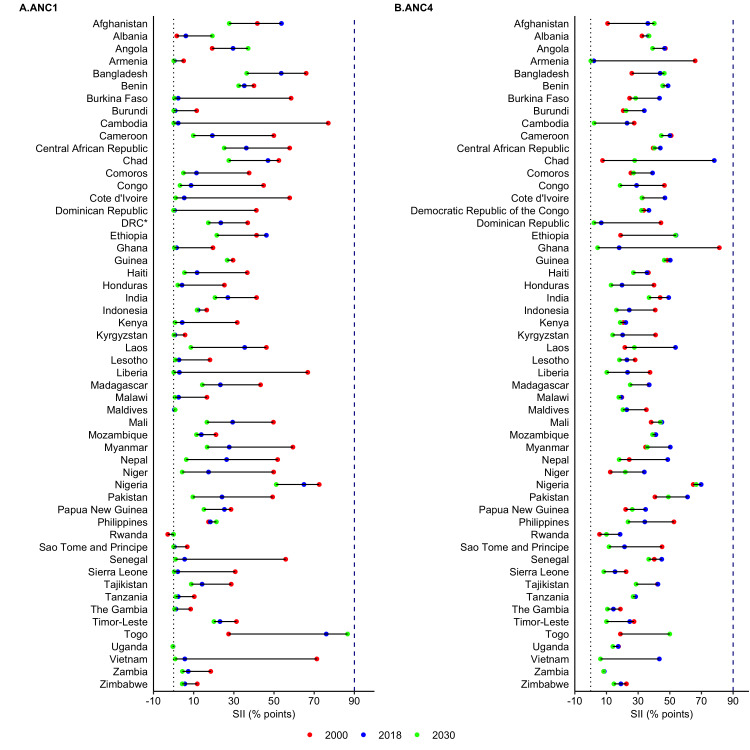
Magnitude of socioeconomic inequalities in access to antenatal care visits among adolescent mothers aged 15-19, 2000-2030. SII - slope index of inequality, ANC1 – single antenatal care visit, ANC4 – at least four antenatal visits, DRC – Democratic Republic of Congo

The ANC coverage by household wealth quintiles was assessed to determine the magnitude of inequalities in access to ANC services among adolescent mothers. The SII values are presented in **Figure 3**. The detailed observable proportion of ANC visits for each quintile and SII values for each country are presented in the **Online Supplementary Document** (Table S7-S9). The richest quintile group in all countries reached ANC1 coverage of 80% or more in 2018 and 2030 (Table S7 in the **Online Supplementary Document**). The poorest quintile in most of the countries is predicted to reach coverage of 80% or more in 2030 except Afghanistan, Angola, Bangladesh, Benin, Central African Republic, Chad, Ethiopia, Guinea, India, Nigeria, Timor-Leste and Togo. For ANC4 (Table S8 in the **Online Supplementary Document**), coverage of 80% or more in 2030 for the richest quintile is predicted in 39 countries compared to 14 countries for the poorest quintile.

In most countries, the inequality in access to ANC services is predicted to decrease substantially from 2000 to 2030, however a few countries such as Afghanistan, Bangladesh, Ethiopia, and Togo demonstrate increasing wealth-based inequality (**Figure 3** and Table S9 in the **Online Supplementary Document**). From 2000 to 2030, predicted reductions in inequalities in ANC1 is highest in Cambodia, Burkina Faso, Cote d’Ivoire, Liberia, Senegal, Togo, and Vietnam; whereas the highest reduction for ANC4 is predicted in Armenia, Chad, and Ghana. The wealth-based inequalities for ANC1 is relatively low compared to ANC4. In 2030, approximately 20 percentage point pro-rich inequality is predicted in 11 countries for ANC1 (Afghanistan, Bangladesh, India, Vietnam, Angola, Ethiopia, Burkina Faso, Central African Republic, Guinea, Nigeria, and Togo) and 31 countries for ANC4. The highest pro-rich inequality for ANC4 (40-50 percentage points) is observed in Afghanistan, Nepal, Pakistan, Myanmar, Papua New Guinea, Ethiopia, Lesotho, Guinea, Liberia, Honduras in 2030; and the lowest inequalities (0-5 percentage points) is observed in Comoros, Maldives, and Laos. No country except Armenia is predicted to achieve zero inequality for ANC4 by 2030, whereas 19 countries (Armenia, Burundi, Cambodia, Cote d’Ivoire, Dominican Republic, Ghana, Kenya, Kyrgyzstan, Lesotho, Liberia, Malawi, Maldives, Rwanda, Sao Tome and Principe, Senegal, Sierra Leone, The Gambia, Uganda, and Vietnam) are predicted to reach zero inequality for ANC1.

### Determinants of ANC visits

In the determinant analysis, approximately 89651 adolescent mothers aged 15-19 years were included to identify the potential factors associated with single and four ANC visits during pregnancy. The estimated odds ratio (OR) with 95% credible interval (CrI) from Bayesian hierarchical model for single and four ANC visits is presented in **Table 2**. Adolescent mothers’ level of education, frequency of access to mass media and household wealth quintile were positively associated with increased odds of ANC1 and ANC4 from skilled health providers. However, though parity was inversely associated with ANC1 and ANC4, the results was not statistically significant. Female household head and those who were aged more than 45 years had increased odds of ANC1 and ANC4 as compared to male household head with age less than 35 years. Adolescent mothers with secondary or higher education had more than double odds to receive ANC1 and ANC4 compared to adolescent mothers with no formal education. Adolescent mothers who had listened to or watched mass-media at least once a week had 1.32 times (95% CrI = 1.29-1.36) higher odds of having ANC1, and 1.44 times (95% CrI = 1.40-1.48) higher odds of ANC4 visits during pregnancy as compared to women who didn’t listen to or watch mass-media at all. Adolescent mothers from the richest households had 1.42 times (95% CrI = 1.36-1.48) higher odds of ANC1, and 1.76 times (95% CrI = 1.67-1.86) higher odds of ANC4 than their poorest counterparts. Adolescent mothers who lived in rural areas had lower odds of ANC1 (12%) and ANC4 (17%) than those who lived in urban areas.

**Table 2:** Determinants of access to professional antenatal care visits among adolescent mothers aged 15-19 years in LMICs

Characteristics	Odds ratio (95% CrI)	
**ANC1**	**ANC4**
HH head age (years):		
<30	1.00	1.00
30-45	1.02 (1.00-1.05)	1.00 (0.97-1.02)
46-60	1.42 (1.37-1.47)	1.20 (1.17-1.23)
>60	1.29 (1.25-1.33)	1.19 (1.17-1.22)
HH head gender:		
Male	1.00	1.00
Female	1.14 (1.10-1.18)	1.05 (1.02-1.07)
Maternal education:		
No education	1.00	1.00
Primary	1.52 (1.46-1.58)	1.66 (1.60-1.72)
Secondary	2.10 (2.04-2.17)	2.28 (2.22-2.35)
Higher	2.22 (2.11-2.33)	2.90 (2.72-3.09)
Parity:		
1	1.00	1.00
2	1.03 (1.00-1.06)	0.99 (0.96-1.03)
≥3	0.98 (0.94-1.01)	0.89 (0.86-0.93)
Mass media:		
None	1.00	1.00
Less than once a week	1.19 (1.16-1.23)	1.16 (1.14-1.19)
At least once a week	1.32 (1.29-1.36)	1.44 (1.40-1.48)
Household wealth quintile:		
Q1 (Poorest)	1.00	1.00
Q2	1.15 (1.12-1.19)	1.29 (1.25-1.33)
Q3	1.26 (1.23-1.31)	1.44 (1.39-1.5)
Q4	1.27 (1.23-1.32)	1.59 (1.53-1.67)
Q5 (Richest)	1.42 (1.36-1.48)	1.76 (1.67-1.86)
Area of residence:		
Urban	1.00	1.00
Rural	0.88 (0.86-0.90)	0.83 (0.81-0.85)

ANC1 – single antenatal care visit, ANC4 – at least four antenatal care visits, CrI – credible intervals, LMIC – low- and middle-income countries

### Sensitivity analysis and model diagnostic

We performed sensitivity analysis by two approaches: exclusion of country-level predictor variables and altering priors for the hyperparameters. Excluding country level predictors, the median absolute differences between the two sets of results were very small: 0.00% (2000) and 0.00% (2030) for single ANC visit posterior means; 0.20% (2000) and 0.10% (2030) for four ANC visits posterior means (Table S10-S11 in the **Online Supplementary Document**). The details of country-year specific mean difference of posterior means between with and without country level predictors are presented in the supplemental appendix (Table S10-S11 in the **Online Supplementary Document**). Though the covariates did not materially alter our inference, we chose to retain them because their inclusion did not impact the model’s DIC (DIC = 1176 (with) vs 1174 (without) for ANC1; DIC = 361.4 (with) vs 356.3 (without) for four ANC visits). After altering prior distribution, there were no significant differences in results (Table S12-S13 in the **Online Supplementary Document**). Altering prior distribution did not change our inference, incorporating half-Cauchy prior did not impact the model’s DIC (DIC = 1175 (flat prior) vs 1176 (weekly prior) for ANC1; DIC = 361.1 (flat prior) vs 362.1 (weekly prior) for ANC4). In case of model diagnostics, the PSRF values indicated that point estimate and upper limit of PSRF for ANC1 and ANC4 visits model are close to 1. The model diagnostics results are presented in the supplemental appendix (Table S14-S15 in the **Online Supplementary Document**).

## DISCUSSION

To our knowledge, this is the first study to provide a comprehensive analysis of the coverage, determinants, and measures of inequality in ANC visits among adolescent mothers, women aged 20-35 years, and those aged 36-49 years, based on nationally representative data from 54 LMICs. The estimates exhibit a steady increase in the proportion of the coverage of ANC visits among all age groups in most countries between 2000 and 2030. Although the rate of ANC4 increases faster than that of ANC1, it fails to reach 80% coverage in most countries by 2030. Maternal education, household head gender, access to mass media, and socio-economic status have been identified as the key determinants for access to ANC services in LMICs. To our knowledge, this is the first attempt to present the coverage of ANC visits among adolescent mothers by countries and different equity strata levels during 2000 to 2030.

Generally, ANC coverage among adolescent mothers has steadily increased between 2000 and 2018, reflecting continuous national efforts to improve reproductive health services in LMICs. By 2030, we anticipate the target ANC1 coverage of 80% will be met by all countries except Angola, Central African Republic and Togo, but only 16 of 54 countries will meet the target for ANC4. The relatively lower coverage of ANC4 among women aged 15-19 years compared to other age groups is reflected in previous studies [13,18-20,29,30]. Qualitative studies with adolescent mothers reflect our findings and propose additional existing barriers such as access to transport, language barriers among ethnic groups, poor navigation of health system, cultural practices, distrust in health providers, and poor satisfaction with the service [24,25,31]. Moreover, focus group study with health providers stated shortage of adolescent-specific guidelines, adolescent-friendly educational materials, and lack of training as the major challenges to providing ANC services [25]. Evidently, interventions building on youth-friendly ANC services and addressing existing barriers will need to be the core elements of health reforms for improving adolescent maternal health.

Our study found the coverage of ANC is reduced with higher number of parities among adolescents, requiring further exploration of reasons influencing the discontinued care. Care seeking behaviour during pregnancy, particularly in adolescent mothers, is complex and shaped by multiple factors of availability, accessibility, and acceptability of ANC services [23]. Perceived usefulness and quality of ANC services are some of the factors which significantly determine women’s determination to return for subsequent visits. Recent analysis using nationally representative surveys in 91 LMICs shows that ANC services is more strongly associated with country’s GDP per capita than with coverage [21]. The ANC ranged from 54% in low-income countries to 93% in upper-middle income countries. While the study includes mothers of all ages, it is possible that the ANC visits among adolescent mothers may be lower due to less bargaining power, lower care expectations, and poorer health literacy. For LMICs to expand ANC services for adolescent mothers, investment of resources to ensuring high-quality care complemented with research to identify key factors promoting retention in maternal health services might be needed to better understand and build on strategic solutions.

In addition to the estimates and trends of ANC coverage among adolescent mothers, we also presented two measures to investigate levels of inequity: socioeconomic and geographical-based coverage. Based on our findings, despite the progress to narrow inequality gap in most countries since 2000, wealth-based and urban-rural inequality gaps will largely continue to exist by 2030 in most LMICs. Consistent with other studies, wide urban-rural disparities for ANC4 coverage was observed in Afghanistan, Nepal, Pakistan, Myanmar, Papua New Guinea, Ethiopia, Lesotho, Guinea, Liberia, and Honduras [19,21,32]. Alternatively, wealth-based inequalities in ANC4 is expected to substantially decrease for Armenia, Cambodia, Dominican Republic, Ghana, Kyrgyzstan, Liberia, Maldives, Sao Tome and Principe, and Vietnam. A large body of work from numerous countries echoes the roles income and geographical location play in utilization of reproductive health services. For adolescent mothers, poverty and rurality not only shape health-related behaviors but also expose them to discrimination, deprivation, or vulnerability in access to ANC services [32-35]. For national strategies aiming to reduce both socioeconomic and geographical inequalities for the poorer adolescent mothers, more efforts need to be exerted on assisting marginalized groups residing in rural areas.

Examples from LMICs’ approach in promoting access and utilization of maternal health services demonstrate possible sustained and targeted strategies. Countries such as Maldives is making an immense progress in this regard, via reforms such as Health Master Plan 2006-2015 and incorporation of social safety net, which is reflected in a relatively low out-of-pocket (OOP) of 21% with high per capita health expenditure (US$1006.9) [36]. Another example is Ghana making tremendous improvements since implementation of the 2004 National Health Insurance Scheme (NHIS), which enables pregnant women to be exempt from premiums with the coverage of maternity care [37]. Additionally for future strategies, institutional practices to reduce unexpected health shocks should be considered such as fees for prescription medications and diagnostics, transportation, and gratuities [22]. Others, like Cambodia, are improving ANC coverage among rural populations despite low per capita health expenditure (US$82.1) [36]. “Outreach Services from the Health Centre” providing ANC services in remote villages as well as reforms shaped by the National Strategic Development Plan (2014) have demonstrated high-impact, cost-effective interventions.

In resource-limited settings, innovation will play a vital role in delivering cost-effective interventions to a wide reach of adolescent mothers. Such innovations are already under way in several African and Asian countries, who are now testing and implementing clinical audits and feedback, community-based interventions, demand side financing, and technology-based approaches to reduce inequalities and promote care-seeking behavior among pregnant mothers [7,38]. Going forward, additional research to build on a reproductive health model tailored for adolescent mothers and reflecting context-appropriate practices will be critical to progress maternal health goals [7,38].

### Strengths and limitations

To our knowledge, our study is the first attempt to describe the trend, projection, and socioeconomic and urban-rural inequality in access to ANC services across LMICs using Bayesian hierarchical approaches. We used the most recent nationally representative household surveys to identify potential determinants of single ANC and four ANC visits among adolescent mothers in LMICs, since they were the primary population of interest in this study. Furthermore, our analysis was based on large data sets with multiple data points, and representative surveys with a high proportion of responses. To assess the magnitude of inequality between the poorest and richest quintiles during 2000 to 2030, we used regression-based equity analysis which considered information of all subgroups in population and offered more complete share of the inequalities. However, some important data limitations in this study must also be highlighted. First, DHS and MICS do not apply unique sampling frame and rather collected information on maternal and child health care services in different recall periods (preceding two for MICS or three years for DHS); therefore, the predicted results could be affected due to utilization of different recall and sampling design. Nonetheless, around 90% of data used in this study were obtained from DHS and few data points from MICS. Second, survey data were not available for all countries and years, and modelling of the outcome was required to fill data gaps for country-years with missing values. These modelled estimates are associated with wide credible interval (CrIs) where there is a single national survey data that complicate result interpretation. However, only one country had single data point and rest of the countries had at least three or more data points; therefore, the predicted CrIs in most countries from 2000 to 2030 were narrow and precise.

## CONCLUSIONS

Our estimates of ANC coverage showed that countries largely achieved high coverage with ANC1 compared to ANC4 over time, but coverage varied greatly by area of residence, socio-economic status, maternal education and access to mass media.

Our findings call for innovative solutions such as technology-based interventions, incorporation of health insurance, and increase in financial investment to health system to promote further access to ANC services, especially in countries where access and equity are still poor.

## Additional material


Online Supplementary Document
