# Supplementary material for: Equity in antenatal care visits among adolescent mothers: An analysis of 54 country levels trend and projection of coverage from 2000 to 2030
Source: J Glob Health. 2022 Mar 19;12:04016. doi: 10.7189/jogh.12.04016 (PMC8932365; doi:10.7189/jogh.12.04016)
Supplement: Online Supplementary Document [file jogh-12-04016-s001.pdf]

Supplementary Document

Equity in antenatal care visits among adolescent mothers: an analysis of 54 country levels trend and projection of coverage from 2000 to 2030

Md. Mizanur Rahman, PhD; Fahima Hossain, MPH; Md. Rashedul Islam, MSc; Jenny Jung, MIPH; Syed Riaz Mahmud, MSc; Masahiro Hashizume, PhD

Table S1: Country specific data Sources

Country	Survey name and year
Afghanistan	DHS 2015; MICS 2010-11
Albania	DHS 2009, 2018
Angola	DHS 2007, 2011
Armenia	DHS 2005, 2010, 2016
Bangladesh	DHS 2004, 2007, 2011, 2014; MICS 2006, 2013
Benin	DHS 2006, 2012, 2018; MICS 2014
Burundi	DHS 2010, 2012, 2017; MICS 2000, 2005
Burkina Faso	DHS 2003, 2010; MICS 2006
Cambodia	DHS 2000, 2005, 2010, 2014
Cameroon	DHS 2004, 2011; MICS 2000, 2006, 2014
Central African Republic	MICS 2000, 2006, 2010
Chad	DHS 2004, 2015; MICS 2000, 2010
Comoros	DHS 2012; MICS 2000
Congo	DHS 2005, 2012; MICS 2015
Congo Democratic Republic	DHS 2007, 2014; MICS 2001, 2010, 2018
Dominican Republic	DHS 2002, 2007, 2013
Ethiopia	DHS 2000, 2005, 2011, 2016
Cote d'Ivoire	MICS 2006, 2016
Ghana	DHS 2003, 2008, 2014; MICS 2006, , 2011
Guinea	DHS 2005, 2012, 2018; MICS 2016
Haiti	DHS 2006, 2012, 2016
Honduras	DHS 2006, 2012
India	DHS 2006, 2016; MICS 2000
Indonesia	DHS 2003, 2007, 2012, 2017
Kenya	DHS 2003, 2009, 2014; MICS 2000
Kyrgyzstan	DHS 2012; MICS 2006, 2014, 2018
Laos	MICS 2000, 2006, 2012, 2017
Lesotho	DHS 2004, 2009, 2014; MICS 2014
Liberia	DHS 2007, 2013, 2016
Madagascar	DHS 2004, 2009; 2011, 2013, 2016; MICS 2000

Malawi	DHS 2000, 2004, 2010, 2016; MICS 2006, 2014
Maldives	DHS 2009, 2017
Mali	DHS 2001, 2006, 2013, 2018; MICS 2010, 2015
Mozambique	DHS 2003, 2011, 2015, 2018; MICS 2008
Myanmar	DHS 2016; MICS 2010
Nepal	DHS 2001, 2006, 2011, 2016; MICS 2014
Niger	DHS 2006, 2012; MICS 2000
Nigeria	DHS 2003, 2008, 2010, 2013, 2015, 2018; MICS 2007, 2011, 2017
Pakistan	DHS 2013, 2018
Papua New Guinea	DHS 2018
Philippines	DHS 2003, 2008, 2013, 2017
Rwanda	DHS 2000, 2005, 2008, 2010, 2015
Sao Tome and Principe	DHS 2009; MICS 2000, 2006, 2014
Senegal	DHS 2005, 2011, 2013, 2014, 2015, 2016, 2017; MICS 2000
Sierra Leone	DHS 2008, 2013; MICS 2000, 2005, 2010, 2017
Tajikistan	DHS 2012, 2017; MICS 2000, 2005
Tanzania	DHS 2005, 2010, 2016, 2017
The Gambia	DHS 2013; MICS 2000, 2006, 2010, 2018
Timor-Leste	DHS 2010, 2016
Togo	DHS 2014, 2017; MICS 2006, 2010
Uganda	DHS 2001, 2006, 2009, 2011, 2015, 2016
Vietnam	DHS 2002, MICS 2000, 2006, 2011, 2014
Zambia	DHS 2002, 2007, 2014, 2018
Zimbabwe	DHS 2006, 2011, 2015; MICS 2009, 2014, 2019

DHS, Demographic and Health Survey; MICS, Multiple Indicator and Cluster Survey

Appendix e-method 1: sensitivity analysis

Both covariates and the model's hierarchical structure influence how data from other countries influence predictions for a given country.[1] We examined the sensitivity of our results by two approaches: (1) the exclusion of country-level covariates (SDI and HRH), and (2) altering priors for the hyperparameters.

Sensitivity analysis: assessing the role of country-level covariates:

Excluding country level predictors, the median absolute differences between the two sets of results and DIC values were compared.

Altering priors for the hyperparameters:

Previous studies reported that to borrow strength and facilitate parameters smoothening from each group, the hyperparameters were shared by all intercept coefficients.[2,3]The key benefit of assigning the hyperparameter is the fact that the resulting model gains the advantages of a complete-pooled model and a no-pooled model. The half-Cauchy is quite heavy tailed and considered as fairly weakly informative. Gelman (2006) advocates for half-t priors (including the half-Cauchy) over the inverse gamma. The hyperparameter priors (hyper-priors) were assigned flat non-informative prior distributions for main analysis and weekly informative hyper-priors for sensitivity analysis. In our proposed model, β_{ij} are conditionally normally distributed on β_k, σ_k^2 while β_k is conditionally normally distributed on $\beta\ and\ \sigma^2$. In the main analysis, $\beta = [\beta_0, \beta_1, \beta_2]$ is the normal distribution was specified in terms of mean zero and the standard deviation of 100, $[\beta_0, \beta_1, \beta_2]$~N(0.0.0001). Since the hyperparameter have some influences on all intercept coefficients, the half-Cauchy distribution (weekly informative prior) applied to perform sensitivity analysis instead of gamma distribution, hyperparameters (τ, σ_k, $and\ \sigma$)~ half-Cauchy (0,25). After altering prior distribution, the median absolute differences between the two sets of results and DIC values were also compared.

Table S2: Coverage of at least single antenatal visits by age group in 54 LMICs, 2000-2030

Country	Predicted coverage, proportion (95% CrI)					
	Aged 20-35 years			Aged 36-49 years		
	**2000**	**2018**	**2030**	**2000**	**2018**	**2030**
**South Asia**						
Afghanistan	21.9 (4.5-56.2)	72.1 (56.6-84.3)	90.5 (62.1-99.2)	17.9 (3.5-51.1)	66.1 (49.6-80.5)	88.0 (56.5-99)
Bangladesh	41.9 (31.1-53.9)	67.6 (57.1-78.0)	80.5 (62.5-92.9)	32.8 (22.8-44.4)	58.5 (46.1-70.1)	73.7 (51.5-89.8)
India	64.0 (46.3-78.1)	80.4 (69.9-87.9)	86.6 (70.0-96.3)	49.2 (30.1-66.4)	68.7 (54.2-80.3)	78.0 (53.8-93.5)
Maldives	99.9 (99.8-100.0)	98.8 (98.1-99.3)	92.1 (78.2-98.1)	99.8 (99.5-99.9)	97.4 (95.7-98.6)	85.3 (63.5-96.3)
Nepal	22.4 (15.8-30.3)	83.8 (77.9-88.6)	97.3 (94.4-98.9)	11.7 (7.4-16.8)	70.3 (60.9-79.0)	94.1 (88.6-97.5)
Pakistan	43.0 (11.7-81.2)	85.4 (79.1-90.2)	94.8 (83.7-99.2)	32.1 (6.9-71.4)	77.1 (67.2-85.5)	91.5 (74.0-98.7)
**East Asia and the Pacific**						
Cambodia	44.7 (35.8-54.2)	98.2 (97.1-99.0)	99.9 (99.7-100.0)	34.0 (26.0-43.8)	97.2 (95.3-98.4)	99.8 (99.6-99.9)
Indonesia	88.6 (82.9-92.9)	97.4 (96.1-98.3)	99.0 (97.8-99.6)	85.7 (78.1-91.4)	96.7 (94.8-98)	98.7 (97.1-99.5)
Laos	23.1 (16.7-30.7)	76.3 (68.3-82.7)	93.9 (87.9-97.2)	16.0 (10.7-22)	67.0 (56.6-76.2)	90.6 (82.0-95.9)
Myanmar	55.0 (4.2-97.9)	86.2 (77.6-92.4)	89.3 (44.1-99.7)	50.0 (3.0-97.3)	81.9 (68.5-91.0)	86.7 (36.0-99.6)
Papua New Guinea	51.8 (2.3-98.4)	77.0 (68.0-84.9)	82.0 (35.0-99.0)	43.4 (1.2-97.5)	65.7 (49.2-78.8)	74.6 (23.2-98.3)
Philippines	86.0 (78.9-91.2)	95.5 (93.5-97)	97.9 (95.3-99.2)	81.4 (71.7-88.8)	93.8 (90.5-96.1)	97.0 (93.4-98.9)
Timor-Leste	84.5 (62.3-96.0)	85.3 (76.6-91.2)	83.0 (52.9-97.0)	79.2 (54.0-94.3)	79.8 (67.8-88.9)	77.6 (43.9-95.9)
Vietnam	76.6 (69.8-82.3)	96.3 (94.3-97.8)	99.0 (97.8-99.6)	78.3 (71.6-84.5)	96.6 (94.6-98.0)	99.1 (98.0-99.7)
**Eastern and Southern Africa**						
Angola	86.7 (76.1-94.0)	79.3 (69.4-86.6)	72.0 (44.1-91.0)	82.5 (68.9-92.0)	73.4 (61.6-82.5)	65.5 (35.8-87.3)
Burundi	83.9 (78.3-88.5)	99.7 (99.5-99.8)	100.0 (100.0-100.0)	78.5 (70.6-84.5)	99.5 (99.3-99.7)	100.0 (99.9-100.0)
Comoros	77.3 (68.6-84.5)	95.8 (92.3-97.9)	98.7 (96.1-99.7)	71.3 (60.3-80.8)	94.3 (89.4-97.2)	98.2 (94.7-99.6)
Ethiopia	23.4 (17.1-30.6)	61.7 (51.7-71.1)	82.7 (68.6-91.9)	16.7 (11.4-23.1)	51.3 (3.09-62.8)	75.8 (58.0-88.2)
Kenya	81.0 (74.7-86.2)	96.3 (94.5-97.6)	98.9 (97.4-99.6)	74.3 (65.5-81.9)	94.7 (91.8-96.8)	98.5 (96.4-99.4)
Lesotho	86.7 (78.2-92.2)	96.2 (93.7-98.1)	98.2 (95.4-99.6)	79.9 (67.3-88.4)	93.8 (89.2-97.1)	97.1 (92.2-99.5)
Madagascar	75.6 (68.5-81.8)	88.6 (84.3-92.0)	93.8 (88.3-97.1)	71.4 (62.6-79.5)	86.2 (80.8-90.8)	92.4 (85.6-96.7)
Malawi	90.8 (87.8-93.3)	97.5 (96.7-98.1)	99.0 (98.2-99.4)	85.9 (80.9-89.8)	96.0 (94.4-97.2)	98.3 (97.1-99.1)
Mozambique	84.8 (77.8-90.2)	93.4 (90.9-95.4)	96.3 (92.5-98.2)	77.6 (68.2-86.2)	89.8 (85.8-93.0)	94.1 (88.7-97.3)
Rwanda	90.4 (86.6-93.3)	99.5 (99.2-99.7)	99.9 (99.8-100.0)	87.2 (81.5-91.4)	99.3 (98.8-99.6)	99.9 (99.8-100.0)
Tanzania	90.7 (84.3-95.0)	98.2 (97.5-98.8)	99.4 (98.6-99.7)	87.8 (79.7-93.7)	97.5 (96.2-98.4)	99.1 (98.0-99.7)
Uganda	92.1 (88.7-94.6)	97.0 (95.8-98.0)	98.5 (97.1-99.4)	88.8 (83.8-92.6)	95.7 (93.9-97.1)	97.8 (95.7-99.0)
Zambia	91.4 (87.6-94.3)	96.7 (95.4-97.7)	98.3 (96.5-99.2)	87.3 (81.0-92.1)	95.0 (92.7-96.9)	97.4 (94.6-98.9)

Zimbabwe	89.4 (81.9-94.9)	93.2 (89.6-95.8)	94.6 (86.0-98.5)	85.7 (76.0-93.0)	90.6 (85.6-94.5)	92.5 (80.7-98.0)
**West and Central Africa**						
Benin	83.5 (73.1-91.2)	84.9 (79.4-89.3)	86.8 (71.7-94.9)	79.3 (66.5-88.9)	80.9 (74.4-86.1)	83.3 (66.2-93.4)
Burkina Faso	61.4 (46.3-74.8)	99.2 (98.0-99.7)	99.9 (99.7-100.0)	53.0 (37.8-67.9)	98.8 (97.0-99.6)	99.9 (99.6-100.0)
Cameroon	80.0 (73.4-85.6)	87.5 (81.0-92.0)	90.9 (79.9-96.3)	75.2 (66.8-82.6)	84.2 (76.1-89.8)	88.4 (74.7-95.3)
Central African Republic	64.0 (53.9-73.5)	75.2 (58.9-87.2)	80.6 (52.3-95.3)	55.2 (43.9-66.5)	67.9 (49.0-82.8)	74.7 (42.6-93.3)
Chad	40.4 (31.7-49.0)	70.0 (59.7-78.9)	83.8 (68.8-93.2)	33.9 (24.9-43.3)	63.8 (51.2-75.1)	79.7 (62.2-91.1)
Congo	78 (65.1-88.3)	96.4 (94.4-97.8)	98.9 (97.2-99.7)	73.2 (57.9-85.5)	95.4 (92.5-97.3)	98.6 (96.2-99.6)
Cote d'Ivoire	76.8 (62.9-87.5)	95.1 (91.8-97.2)	98.3 (95.5-99.5)	70.2 (54.4-83.4)	93.1 (88.3-96.2)	97.5 (93.2-99.4)
Democratic Republic of the Congo	80.0 (73.0-86.4)	88.0 (83.8-91.5)	91.8 (84.7-96.2)	75.1 (66.2-83.1)	84.7 (78.5-89.5)	89.4 (79.3-95.1)
Ghana	90.3 (85.9-93.8)	98.1 (96.9-99.0)	99.3 (98.3-99.8)	85.6 (78.6-91.1)	97.1 (95.2-98.4)	99.0 (97.3-99.7)
Guinea	83.8 (74.4-90.6)	86.4 (82.1-90.0)	87.4 (77.1-94.1)	77.4 (64.5-87.4)	80.7 (73.7-86.4)	82.2 (67.2-91.4)
Liberia	44.7 (25.4-65.4)	98.8 (98.0-99.3)	99.9 (99.8-100.0)	36.1 (17.5-57.3)	98.2 (97.0-99.0)	99.9 (99.7-100.0)
Mali	59.5 (48.2-70.0)	82.2 (77.0-86.7)	91.1 (84.4-95.7)	51.5 (39.0-63.4)	76.8 (69.6-83.3)	88.0 (78.3-94.0)
Niger	38.7 (28.8-48.9)	92.1 (85.9-96.0)	98.7 (96.3-99.7)	33.7 (23.2-46.1)	90.2 (81.6-95.4)	98.3 (95.0-99.6)
Nigeria	54.5 (44.1-64.6)	67.1 (60.0-73.4)	74.5 (60.6-85.3)	52.0 (41.3-62.7)	64.7 (56.9-72.2)	72.5 (56.7-84.5)
Sao Tome and Principe	91.6 (87.7-94.6)	99.5 (99.2-99.7)	99.9 (99.8-100.0)	78.5 (69.2-86.2)	98.5 (97.5-99.2)	99.8 (99.5-99.9)
Senegal	81.3 (75.1-86.1)	97.1 (96.4-97.8)	99.2 (98.8-99.6)	75.9 (68.3-82.3)	96.1 (94.7-97.1)	99.0 (98.2-99.4)
Sierra Leone	68.7 (61.1-76.0)	98.5 (97.9-98.9)	99.8 (99.7-99.9)	60.3 (50.7-69.7)	97.8 (96.7-98.5)	99.8 (99.5-99.9)
The Gambia	96.8 (94.5-98.3)	99.3 (98.9-99.5)	99.7 (99.3-99.9)	96.2 (93.2-98.2)	99.2 (98.7-99.5)	99.7 (99.2-99.9)
Togo	89.5 (81.9-94.6)	63.3 (53.2-72.5)	38.5 (18.5-62.6)	86.5 (76.8-93.3)	56.7 (44.4-68.2)	32.5 (13.6-56.1)
**Latin America and Caribbean**						
Dominican Republic	56.7 (33.1-80.0)	99.9 (99.8-100.0)	100.0 (100.0-100.0)	52.3 (29.3-75.7)	99.9 (99.8-100.0)	100.0 (100.0-100.0)
Haiti	80.8 (67.9-89.9)	92.3 (88.1-95.1)	95.8 (89.2-98.8)	73.3 (56.8-86.1)	88.5 (82.0-93.2)	93.8 (84.7-98.3)
Honduras	84.4 (70.6-93.1)	98.2 (96.4-99.3)	99.5 (98.0-99.9)	78.9 (60.8-90.2)	97.4 (94.2-99.0)	99.2 (96.8-99.9)
**Central and Eastern Europe**						
Albania	99.2 (98.2-99.7)	89.9 (85.6-93.4)	60.6 (35.4-83.4)	98.9 (97.4-99.6)	86.3 (80.1-91.3)	52.7 (25.9-77.8)
Armenia	82.6 (71.8-89.9)	99.9 (99.9-100.0)	100.0 (100.0-100.0)	79.8 (67.8-88.7)	99.9 (99.9-100.0)	100.0 (100.0-100.0)
Kyrgyzstan	81.6 (69.2-90.1)	99.7 (99.5-99.8)	100.0 (100.0-100.0)	78.0 (64.1-88.5)	99.6 (99.3-99.7)	100.0 (99.9-100.0)
Tajikistan	72.2 (63.4-79.3)	90.7 (86.6-93.9)	96.0 (91.9-98.4)	58.2 (47.1-68.7)	83.9 (76.6-89.6)	92.7 (85.3-97.1)

LMICs, low-and middle-income countries; CrI, credible interval

Table S3: Coverage of at least four antenatal visits by age group in 54 LMICs, 2000-2030

Country	Predicted coverage, proportion (95% CrI)					
	Aged 20-35 years			Aged 36-49 years		
	**2000**	**2018**	**2030**	**2000**	**2018**	**2030**
**South Asia**						
Afghanistan	5.6 (1.7-12.9)	24.8 (17.2-33.8)	53.4 (23.8-82.5)	5.1 (1.5-11.4)	23 (15.8-32.3)	51.1 (21.5-81.3)
Bangladesh	13.7 (9.7-18.3)	29.1 (22.7-35.3)	44.3 (27.0-60.8)	7.5 (5.1-10.3)	17.3 (12.8-22.6)	29.1 (16.4-44.3)
India	22.3 (14.9-31.4)	54.1 (44.7-62.7)	74.8 (58.3-86.9)	12.7 (7.7-19.1)	37.4 (29.0-47.0)	60.4 (42.2-77.0)
Maldives	85.8 (76.7-92.4)	85.2 (80.7-88.9)	84.1 (70.7-92.7)	82.3 (71.7-90.5)	81.5 (74.8-86.7)	80.2 (63.5-90.9)
Nepal	8.7 (6.5-11.1)	68.2 (62.5-73.5)	94.6 (91.5-96.6)	3.5 (2.6-4.6)	44.9 (38.0-52.0)	86.9 (80.3-92.0)
Pakistan	15.5 (5.3-34.9)	49.6 (42.4-57.1)	75.3 (55.3-89.6)	11.3 (3.5-25.9)	40.3 (32.1-48.6)	68.0 (44.6-86.3)
**East Asia and the Pacific**						
Cambodia	11.6 (9.1-14.6)	89.2 (85.6-92.1)	99.2 (98.6-99.6)	7.3 (5.5-9.3)	83.0 (77.2-87.8)	98.7 (97.7-99.4)
Indonesia	74.4 (67.9-80.3)	90.4 (87.5-92.7)	95.3 (92.1-97.5)	69.3 (61.2-76.3)	87.9 (84.4-91.0)	94.0 (90.2-96.8)
Laos	8.9 (3.1-18.0)	60.5 (52.2-69.1)	90.3 (78.8-96.9)	6.8 (2.3-15.3)	53.1 (43.4-62.4)	87.4 (73.3-96)
Myanmar	38.5 (6.9-81.6)	60.0 (49.7-69.6)	71.3 (29.7-94.6)	37.3 (6.2-81.1)	58.4 (46.1-69.2)	70.1 (29.0-94.1)
Papua New Guinea	34.8 (5.0-78.4)	49.2 (41.3-57.1)	60.6 (26.6-88.0)	28.1 (3.4-72.6)	39.7 (29.8-49.5)	52.1 (19.5-83.3)
Philippines	63.7 (55.7-71.0)	86.4 (83.0-89.3)	93.7 (90.1-96.3)	56.0 (47.7-64.0)	82.2 (77.4-86.2)	91.5 (86.4-95.0)
Timor-Leste	26.1 (13.2-43.5)	80.1 (72.9-86.0)	95.2 (88.2-98.5)	21.2 (10.2-36.3)	75.2 (66.5-82.9)	93.7 (85.0-98.2)
Vietnam	26.4 (19.8-33.3)	75.4 (68.6-81.6)	92.6 (86.7-96.5)	28.9 (21.9-37.0)	77.6 (70.2-84.3)	93.3 (87.6-96.9)
**Eastern and Southern Africa**						
Angola	33.6 (5.1-76.6)	65.5 (56.0-74.4)	80.6 (47.0-97.3)	29.6 (4.0-73.3)	60.2 (49.5-70.8)	77.3 (38.4-96.8)
Burundi	18.9 (9.4-31.6)	55.2 (47.4-63.2)	78.8 (62.3-90.8)	15.9 (7.5-27.1)	49.9 (40.7-59.2)	75.1 (56.2-89.3)
Comoros	32.2 (7.8-65.9)	60.0 (41.0-77.5)	73.6 (28.5-97.0)	29.7 (7.4-66.6)	57.0 (37.5-75.9)	71.4 (26.7-97.0)
Ethiopia	8.8 (6.9-11.1)	32.1 (26.3-38.5)	57.9 (45.2-69.9)	5.9 (4.4-7.7)	23.7 (18.7-29.4)	47.5 (34.9-60.0)
Kenya	44.9 (36.0-53.6)	61.0 (53.7-67.9)	71.7 (56.9-83.6)	39.5 (30.8-48.5)	55.6 (47.3-63.7)	67 (51.5-80.9)
Lesotho	66.2 (56.4-74.7)	76 (68.8-82.5)	81.2 (67.2-90.7)	62.9 (52.5-72.8)	73.3 (64.5-80.4)	79.0 (63.6-89.3)
Madagascar	34.8 (23.7-46.4)	59.4 (39.8-76.7)	73.1 (39.3-93.8)	31.6 (21.4-42.9)	55.9 (35.8-74.9)	70.3 (35.0-93.2)
Malawi	54.4 (48.3-60.5)	45.3 (39.2-51.4)	39.8 (28.7-50.5)	49.7 (43.4-55.9)	40.6 (34.0-47.0)	35.4 (24.4-46.0)
Mozambique	55.0 (46.5-63.5)	52.3 (45.9-58.3)	50.8 (37.7-62.8)	49.2 (40.2-58.9)	46.5 (39.8-53.4)	45.1 (32.3-57.8)
Rwanda	9.8 (7.7-12.3)	58.4 (50.6-65.7)	88.8 (81.3-93.7)	8.4 (6.4-10.8)	54.0 (45.9-62.1)	86.9 (78.1-92.8)
Tanzania	52.3 (43.1-61.7)	53.9 (47.6-59.7)	55.1 (41.6-67.5)	46.9 (37.1-57.1)	48.4 (41.2-55.8)	49.7 (36.2-63.4)
Uganda	39.6 (32.8-46.4)	61.3 (54.3-68.2)	74.0 (62.9-83.5)	33.7 (27.2-40.8)	55.0 (46.8-63.4)	68.8 (55.7-80.0)
Zambia	66.0 (58.9-72.6)	59.0 (52.7-65.0)	54.3 (42.2-65.9)	63.7 (56.1-70.9)	56.5 (49.2-63.4)	51.8 (39.0-63.9)
Zimbabwe	55.8 (44.2-65.8)	74.0 (68.3-79.7)	83.0 (71.9-91.4)	52.1 (40.1-62.3)	71.0 (64.1-77.5)	80.8 (68.3-90.5)

**West and Central Africa**						
Benin	62.0 (51.3-71.3)	52.1 (45.6-58.3)	47.2 (32.1-61.8)	57.1 (45.6-67.5)	46.9 (40.6-53.5)	42.2 (28.4-57.0)
Burkina Faso	14.3 (10.0-19.8)	55.5 (41.4-69.3)	81.6 (61.9-94.0)	11.7 (7.6-16.8)	49.8 (35.6-64.2)	78.0 (56.5-92.5)
Cameroon	61.3 (51.8-70.2)	60.6 (51.4-68.8)	60.3 (41.8-76.4)	57.3 (46.9-66.5)	56.5 (46.1-65.4)	56.3 (36.8-73.2)
Central African Republic	32.2 (25.9-39.6)	39.9 (28.3-52.1)	46.0 (24.6-67.1)	28.0 (21.5-35.5)	35.2 (23.5-48.6)	41.2 (20.6-63.5)
Chad	12.9 (9.1-17.6)	38.9 (30.4-47.3)	62.6 (44.7-77.9)	10.9 (7.4-15.6)	34.5 (26.2-43.8)	58.1 (39.9-75.0)
Congo	66.6 (55.9-75.9)	83.9 (78.5-88.3)	90.7 (82.9-95.7)	63.2 (51.4-73.5)	81.7 (75.3-86.9)	89.3 (80.6-95.1)
Cote d'Ivoire	34.2 (4.5-78.4)	55.5 (45.9-65.6)	68.6 (26.7-94.7)	30.4 (3.6-74.7)	50.3 (38.6-61.4)	64.6 (22.3-93.7)
Democratic Republic of the Congo	45.1 (34.9-55.5)	46.9 (40.3-53.2)	49.1 (34.2-63.0)	40.2 (30.3-51.4)	42.0 (35.4-48.8)	44.2 (29.5-58.9)
Ghana	63.3 (55.7-70.4)	89.7 (86.2-92.7)	96.1 (92.9-98.2)	60.5 (52.4-68.3)	88.5 (84.2-91.9)	95.6 (91.8-98.0)
Guinea	54.5 (44.3-64.1)	47.9 (41.6-54.1)	43.8 (31.8-56.9)	50.6 (40.4-60.3)	44.0 (37.5-50.3)	40.0 (27.6-53.2)
Liberia	45.6 (33.3-59.7)	83.4 (78.4-87.5)	94.0 (88.6-97.3)	42.5 (29.5-56.2)	81.5 (75.4-86.4)	93.3 (86.4-96.9)
Mali	30.4 (24.1-37.4)	44.1 (38.8-49.4)	54.6 (43.5-65.1)	26.9 (20.5-33.9)	39.8 (33.9-45.5)	50.2 (39.0-60.7)
Niger	8.6 (4.6-14.3)	52.4 (39.2-65.7)	83.9 (64.0-95.0)	8.1 (4.3-13.6)	50.7 (35.8-65.1)	82.9 (60.8-95.0)
Nigeria	44.5 (37.7-51.4)	51.5 (46.1-56.4)	56.5 (45.7-65.9)	43.5 (36.4-50.5)	50.5 (44.9-56.6)	55.5 (44.0-66.4)
Sao Tome and Principe	58.2 (37.3-76.1)	86.7 (79.8-91.4)	94.1 (83.5-98.6)	50.7 (31.4-70.9)	82.6 (74.0-89.4)	92.1 (78.1-98.1)
Senegal	34.3 (26.3-44.1)	54.8 (49.7-59.7)	68.1 (56.2-77.7)	29.3 (21.6-38.8)	48.9 (43.6-54.4)	62.8 (51.1-73.4)
Sierra Leone	45.8 (33.4-59.3)	81.2 (75.8-85.3)	92.5 (85.7-96.4)	41.1 (28.9-54.3)	78.0 (71.9-83.5)	91.0 (83.0-95.9)
The Gambia	69.6 (59.7-77.8)	78.0 (73.1-82.2)	82.5 (72.4-89.7)	69.1 (59.5-78.0)	77.6 (72.0-82.3)	82.1 (71.6-89.7)
Togo	34.5 (16.2-57.3)	50.8 (38.1-63.1)	61.5 (28.2-87.2)	29.8 (12.8-52.0)	45.2 (32.5-58.7)	56.5 (24.1-84.2)
**Latin America and Caribbean**						
Dominican Republic	82.3 (71.7-89.9)	96.4 (94.3-98.0)	98.8 (96.6-99.7)	77.8 (64.4-87.3)	95.3 (92.3-97.3)	98.3 (95.5-99.6)
Haiti	46.3 (35.0-57.6)	69.7 (62.8-75.8)	81.9 (69.3-90.6)	42.1 (30.9-53.7)	66.0 (57.6-73.7)	79.2 (64.2-89.1)
Honduras	93.7 (89.3-96.4)	85.5 (76.5-91.5)	74.8 (46.1-92.2)	91.8 (86.1-95.5)	81.5 (70.9-89.8)	69.3 (38.3-90.2)
**Central and Eastern Europe**						
Albania	63.7 (49.9-78.2)	77.9 (72.4-82.4)	84.7 (72.8-92.1)	57.8 (42.4-73.2)	73.2 (66.1-79.3)	81.2 (67.1-90.5)
Armenia	61.9 (51.8-71.4)	97.8 (96.9-98.4)	99.8 (99.5-99.9)	66.3 (55.7-75.7)	98.2 (97.4-98.8)	99.8 (99.6-99.9)
Kyrgyzstan	82.1 (66.5-92.1)	93.0 (90.8-94.7)	96.1 (91.4-98.5)	78.0 (61.5-89.8)	91.1 (88.1-93.6)	95.1 (89.3-98.2)
Tajikistan	29.4 (11.9-52.3)	68.1 (60.3-75.3)	86.0 (69.4-95.5)	21.6 (7.9-39.6)	58.0 (47.4-67.4)	80.1 (58.6-93.2)

LMICs, low-and middle-income countries; CrI, credible interval

Table S4: Coverage of at least single ANC visit among adolescent mothers according area of residence in 54 LMICs, 2000-2030

Country	Urban (predicted coverage, proportion with 95% CrI)			Rural (predicted coverage, proportion with 95% CrI)		
	**2000**	**2018**	**2030**	2000	2018	2030
**South Asia**						
Afghanistan	23.6 (8.9-44.7)	86.2 (72.1-94.2)	97.5 (90.2-99.7)	8.9 (3.0-20.2)	66.6 (46.4-81.6)	92.8 (73.4-99.0)
Bangladesh	62.0 (41.5-78.0)	81.2 (65.9-91.1)	87.7 (62.8-97.9)	40.0 (23.7-58.7)	63.8 (45.8-79.1)	75.5 (42.4-94.3)
India	82.5 (59.9-94.8)	90.3 (79.2-96.4)	91.8 (69.1-99.1)	66.1 (38.5-87.0)	78.9 (60.7-91.0)	82.6 (45.5-97.6)
Maldives	100.0 (99.9-100.0)	99.8 (99.5-99.9)	98.6 (94.2-99.9)	99.9 (99.6-100.0)	99.3 (98.4-99.7)	95.9 (83.6-99.7)
Nepal	62.2 (45.2-78.2)	94.0 (89.3-97.1)	98.5 (95.6-99.6)	31.2 (17.0-47.0)	80.9 (69.5-89.8)	94.5 (85.7-98.6)
Pakistan	56.0 (25.8-83.2)	89.7 (81.1-95.5)	96.1 (86.2-99.4)	39.7 (15.5-69.4)	81.1 (67.0-90.2)	92.6 (75.5-98.8)
**East Asia and the Pacific**						
Cambodia	76.9 (62.8-88.0)	99.7 (99.3-99.9)	100.0 (99.9-100.0)	40.4 (24.8-56.4)	98.4 (96.6-99.3)	99.9 (99.7-100.0)
Indonesia	94.0 (88.1-97.6)	97.8 (95.8-99.0)	98.7 (96.1-99.7)	83.6 (71.0-92.4)	93.5 (88.2-96.9)	96.1 (88.5-99.2)
Laos	47.1 (29.0-64.4)	90.3 (82.1-95.5)	97.6 (93.2-99.5)	17.9 (9.2-29.0)	69.3 (52.5-82.7)	90.8 (76.5-97.8)
Myanmar	89.3 (61.3-98.8)	93.7 (85.5-97.9)	92.2 (64.1-99.6)	69.8 (26.1-95.3)	78.2 (58.6-90.7)	77.4 (31.6-98.3)
Papua New Guinea	66.4 (5.0-99.3)	89.4 (77.0-95.9)	90.8 (54.1-99.7)	51.2 (1.9-98.2)	75.7 (55.9-89.6)	81.2 (30.6-99.1)
Philippines	91.3 (82.9-96.5)	96.3 (93.2-98.3)	97.6 (93.0-99.5)	82.9 (68.8-92.3)	92.2 (85.2-96.3)	94.8 (84.4-99.0)
Timor-Leste	85.8 (58.9-98.0)	94.2 (87.0-97.9)	94.5 (74.3-99.7)	67.2 (26.5-94.0)	83.1 (66.3-93.6)	85.7 (44.9-99.1)
Vietnam	99.3 (98.7-99.7)	99.9 (99.7-99.9)	99.9 (99.8-100.0)	64.3 (48.7-77.4)	89.6 (80.8-95.4)	95.6 (86.8-99.1)
**Eastern and Southern Africa**						
Angola	98.8 (96.6-99.7)	92.5 (84.4-97.2)	73.8 (34.8-96.5)	91.7 (79.0-97.7)	61.9 (41.9-78.8)	32.8 (5.8-74.0)
Burundi	81.5 (69.2-90.3)	99.2 (98.4-99.6)	99.9 (99.7-100.0)	81.1 (70.5-89.1)	99.1 (98.5-99.5)	99.9 (99.7-100.0)
Comoros	80.9 (64.6-91.4)	95.2 (88.2-98.6)	97.9 (91.2-99.8)	70.2 (53.8-83.0)	91.7 (81.4-97.2)	96.2 (85.6-99.6)
Ethiopia	53.3 (36.2-70.8)	92.3 (84.7-96.6)	98.1 (93.8-99.6)	16.7 (9.3-27.6)	67.8 (51.0-81.6)	90.1 (73.5-97.5)
Kenya	79.1 (67.1-88.1)	96.2 (92.9-98.2)	98.8 (96.6-99.8)	73.4 (60.9-84.6)	94.9 (90.5-97.5)	98.4 (95.5-99.7)
Lesotho	91.3 (80.6-96.9)	98.5 (96.2-99.5)	99.3 (97.1-99.9)	82.8 (65.6-94.1)	96.7 (92.1-98.9)	98.6 (93.8-99.9)
Madagascar	83.3 (73.5-90.9)	94.4 (90.0-97.1)	97.2 (92.4-99.2)	63.6 (49.3-77.0)	85.3 (75.4-91.6)	92.5 (80.5-97.7)
Malawi	96.0 (93.4-97.8)	99.2 (98.6-99.5)	99.7 (99.3-99.9)	90.3 (84.8-94.4)	97.8 (96.5-98.7)	99.2 (98.1-99.7)
Mozambique	95.6 (91.3-98.2)	96.7 (94.3-98.3)	96.8 (91.6-99.2)	87.9 (77.6-94.3)	90.5 (84.6-94.9)	91.1 (77.7-97.6)
Rwanda	84.3 (73.2-91.5)	99.6 (99.3-99.8)	100.0 (99.9-100.0)	86.3 (77.1-92.7)	99.7 (99.4-99.9)	100.0 (99.9-100.0)
Tanzania	96.5 (92.0-98.8)	98.9 (98.0-99.5)	99.4 (98.2-99.9)	91.0 (80.9-96.6)	97.1 (94.7-98.7)	98.4 (95.3-99.7)
Uganda	94.4 (90.1-97.4)	96.7 (94.2-98.3)	97.4 (93.5-99.3)	93.0 (87.5-96.7)	95.8 (92.7-97.8)	96.7 (91.3-99.1)
Zambia	96.7 (93.3-98.6)	98.7 (97.6-99.4)	99.2 (98.0-99.8)	89.5 (80.8-95.3)	95.7 (92.5-98.0)	97.4 (93.5-99.3)
Zimbabwe	88.1 (74.9-96.0)	92.4 (85.7-96.7)	93.0 (75.8-99.1)	87.5 (73.9-95.7)	92.0 (84.5-96.5)	92.6 (74.4-99.0)
**West and Central Africa**						

Benin	84.7 (67.2-94.5)	89.2 (81.0-94.5)	90.6 (73.6-97.8)	71.8 (48.3-88.3)	78.8 (67.2-87.2)	81.9 (54.3-95.3)
Burkina Faso	92.8 (85.1-97.3)	99.5 (98.4-99.9)	99.9 (99.3-100.0)	70.6 (51.0-85.0)	97.5 (92.3-99.4)	99.3 (96.3-100.0)
Cameroon	88.2 (80.3-94.2)	96.3 (92.2-98.5)	98.2 (93.6-99.6)	63.7 (49.0-77.2)	86.0 (73.9-93.4)	92.7 (78.3-98.5)
Central African Republic	84.6 (72.7-92.7)	90.3 (77.2-97.4)	91.6 (67.0-99.3)	54.9 (38.4-71.3)	68.5 (43.0-88.4)	74.1 (32.2-96.8)
Chad	62.8 (45.9-78.1)	86.8 (75.4-94.2)	93.6 (82.0-98.7)	32.0 (19.1-46.7)	65.0 (46.7-80.2)	81.0 (55.0-95.0)
Congo	88.3 (75.2-96.5)	96.7 (92.8-98.8)	98.2 (92.3-99.8)	73.7 (52.6-89.4)	91.3 (83.2-96.3)	95.1 (82.8-99.4)
Cote d'Ivoire	89.3 (74.3-96.8)	97.0 (92.8-98.9)	98.4 (93.7-99.8)	75.2 (48.9-90.9)	92.1 (84.1-96.9)	95.7 (85.0-99.4)
Democratic Republic of the Congo	88.5 (79.6-94.4)	92.3 (86.7-96.1)	93.7 (84.0-98.4)	75.2 (60.8-86.3)	82.5 (72.6-89.5)	85.7 (67.9-95.3)
Ghana	96.8 (93.7-98.6)	99.4 (98.7-99.8)	99.7 (99.0-100.0)	90.1 (81.0-95.3)	97.8 (95.4-99.1)	99.1 (96.7-99.9)
Guinea	94.5 (86.7-98.1)	95.1 (91.0-97.6)	94.7 (83.9-98.9)	81.3 (61.3-92.9)	82.8 (73.3-90.0)	82.1 (60.4-95.0)
Liberia	67.6 (36.0-89.9)	99.4 (98.8-99.8)	100.0 (99.8-100.0)	44.8 (17.1-75.7)	98.4 (96.8-99.4)	99.9 (99.5-100.0)
Mali	80.9 (67.2-90.4)	93.9 (90.0-96.6)	97.1 (93.1-99.2)	51.3 (32.7-69.3)	78.9 (67.4-87.9)	89.2 (74.7-96.6)
Niger	75.6 (58.7-87.3)	97.8 (94.8-99.3)	99.5 (98.1-99.9)	34.7 (19.7-51.7)	88.0 (73.2-95.8)	97.2 (89.3-99.7)
Nigeria	65.6 (50.5-79.0)	76.9 (65.8-85.4)	82.1 (62.5-93.4)	36.1 (22.4-51.4)	49.4 (36.8-62.4)	58.4 (33.4-79.6)
Sao Tome and Principe	91.9 (83.8-96.6)	100.0 (100.0-100.0)	100.0 (100.0-100.0)	81.6 (68.5-91.6)	100.0 (99.9-100.0)	100.0 (100.0-100.0)
Senegal	92.4 (87.1-96.0)	99.0 (98.3-99.4)	99.7 (99.4-99.9)	75.0 (62.3-85.3)	95.8 (93.5-97.6)	98.8 (97.4-99.6)
Sierra Leone	79.4 (67.5-88.5)	99.3 (98.6-99.6)	99.9 (99.8-100.0)	62.6 (46.7-76.6)	98.3 (97.0-99.2)	99.8 (99.5-100.0)
The Gambia	97.8 (94.5-99.3)	99.4 (98.9-99.7)	99.7 (99.1-99.9)	96.0 (90.2-98.8)	98.9 (97.9-99.5)	99.5 (98.3-99.9)
Togo	94.1 (86.3-98.0)	87.9 (78.3-94.0)	79.1 (49.2-94.9)	72.3 (48.4-88.3)	53.8 (36.7-70.3)	41.2 (13.7-74.9)
**Latin America and Caribbean**						
Dominican Republic	71.2 (35.9-92.8)	99.9 (99.6-100.0)	100.0 (100.0-100.0)	65.1 (29.8-89.0)	99.8 (99.5-100.0)	100.0 (100.0-100.0)
Haiti	82.0 (61.0-94.0)	93.3 (86.9-97.4)	95.9 (86.2-99.5)	75.8 (51.7-91.1)	90.5 (82.1-95.7)	94.2 (79.6-99.1)
Honduras	87.7 (68.3-97.0)	98.4 (95.0-99.7)	99.2 (95.4-100.0)	80.9 (56.3-94.6)	97.3 (92.0-99.4)	98.8 (92.3-100.0)
**Central and Eastern Europe**						
Albania	96.0 (87.9-99.3)	89.9 (80.3-95.8)	78.4 (41.3-96.5)	96.0 (87.5-99.3)	90.0 (81.9-95.0)	78.5 (42.4-95.9)
Armenia	86.5 (70.1-95.4)	99.9 (99.9-100.0)	100.0 (100.0-100.0)	83.5 (66.1-93.6)	99.9 (99.8-100.0)	100.0 (100.0-100.0)
Kyrgyzstan	96.5 (91.4-99.1)	99.9 (99.8-100.0)	100.0 (100.0-100.0)	83.9 (65.3-94.6)	99.4 (98.9-99.7)	99.9 (99.7-100.0)
Tajikistan	84.2 (73.3-92.0)	95.5 (91.5-98.0)	98.0 (94.3-99.5)	72.7 (58.4-84.5)	91.2 (83.2-95.9)	95.9 (88.4-99.0)

LMICs, low-and middle-income countries; CrI, credible interval

Table S5: Coverage of at least four ANC visits among adolescent mothers according area of residence in 54 LMICs, 2000-2030

Country	Urban (predicted coverage, proportion with 95% CrI)			Rural (predicted coverage, proportion with 95% CrI)		
	2000	2018	2030	2000	2018	2030
**South Asia**						
Afghanistan	9.0 (2.3-21.4)	39.1 (25.1-55.6)	70.1 (38.1-93.0)	3.1 (0.8-7.9)	17.2 (10.5-26.0)	46.0 (16.9-80.7)
Bangladesh	26.3 (17.8-36.2)	42.7 (32.1-54.0)	55.2 (33.4-75.6)	11.9 (7.6-17.8)	22.0 (15.7-30.2)	32.6 (16.6-53.8)
India	43.7 (29.2-60.9)	69.7 (58.3-80.5)	82.1 (66.4-92.9)	23.9 (14.4-37.2)	48.2 (35.7-60.9)	65.4 (44.1-83.5)
Maldives	99.9 (99.7-99.9)	99.9 (99.7-99.9)	99.8 (99.5-100.0)	85.9 (73.1-94.0)	85.2 (77.2-91.3)	83.5 (63.8-94.6)
Nepal	30.2 (22.1-39.2)	79.2 (71.9-85.2)	94.2 (89.4-97.2)	13.3 (9.2-18.2)	57.4 (47.5-67.0)	85.3 (74.6-92.3)
Pakistan	28.0 (15.5-44.4)	56.7 (45.9-67.3)	74.3 (53.7-88.7)	13.9 (6.5-24.7)	34.8 (24.6-45.4)	55.0 (31.9-76.1)
**East Asia and the Pacific**						
Cambodia	30.1 (22.2-38.6)	93.9 (90.4-96.4)	99.4 (98.6-99.8)	12.1 (8.3-16.2)	83.1 (75.5-88.6)	98.1 (95.9-99.2)
Indonesia	78.8 (69.6-86.2)	89.7 (85.3-93.3)	93.7 (88.1-97.2)	65.3 (54.2-75.1)	81.4 (74.3-87.4)	88.2 (78.3-94.7)
Laos	25.5 (8.4-53.8)	70.2 (58.8-79.9)	88.9 (72.1-97.2)	9.6 (2.6-25.2)	41.1 (29.3-53.5)	71.5 (42.2-90.7)
Myanmar	51.6 (13.8-87.6)	72.2 (58.3-83.4)	80.2 (48.7-96.1)	22.8 (3.0-63.2)	37.1 (23.9-52.9)	51.7 (16.9-84.3)
Papua New Guinea	40.1 (7.1-81.7)	53.6 (37.5-68.3)	63.2 (30.1-89.3)	35.5 (5.7-80.0)	47.9 (32.4-62.6)	58.2 (26.2-87.4)
Philippines	69.2 (57.5-78.8)	84.2 (77.7-89.2)	90.2 (81.9-95.6)	59.8 (48.0-71.5)	77.8 (70.0-84.7)	85.8 (75.3-93.6)
Timor-Leste	34.0 (14.1-58.0)	82.3 (72.5-89.7)	95.0 (85.8-99.0)	24.8 (9.6-46.9)	74.5 (62.9-84.1)	92.3 (77.7-98.4)
Vietnam	45.6 (33.3-59.3)	75.4 (64.9-83.7)	87.4 (74.6-94.8)	18.9 (11.8-27.6)	46.0 (33.2-58.7)	66.5 (43.6-83.8)
**Eastern and Southern Africa**						
Angola	63.2 (43.3-80.3)	78.0 (66.4-86.4)	84.5 (65.6-95.1)	26.7 (13.2-44.7)	41.9 (30.3-53.9)	54.6 (30.2-78.2)
Burundi	24.7 (10.3-44.1)	54.4 (41.0-67.1)	73.6 (49.2-91.0)	19.5 (8.2-36.2)	46.6 (36.2-58.4)	67.6 (43.0-87.3)
Comoros	28.6 (8.0-59.0)	50.2 (28.7-72.1)	64.4 (21.4-94.1)	24.0 (6.8-51.9)	44.1 (25.1-63.4)	59.5 (18.4-92.6)
Ethiopia	31.5 (23.1-40.2)	66.7 (55.7-76.3)	84.0 (71.2-92.2)	6.5 (4.3-9.2)	23.3 (16.0-31.7)	45.2 (27.6-64.0)
Kenya	42.3 (29.9-54.2)	56.8 (46.0-66.9)	67.7 (46.8-83.6)	35.2 (24.9-47.2)	49.4 (38.5-60.2)	61.0 (39.8-79.4)
Lesotho	79.1 (68.3-86.9)	77.8 (66.2-86.1)	76.6 (54.3-91.1)	65.4 (52.6-76.5)	63.6 (51.1-75.1)	62.7 (38.6-83.5)
Madagascar	44.0 (28.0-60.9)	70.9 (48.8-86.7)	82.4 (49.7-97.3)	26.9 (16.6-41.4)	53.8 (32.3-75.1)	70.3 (32.1-94.3)
Malawi	63.2 (54.7-71.2)	48.4 (39.7-57.2)	39.4 (26.2-55.3)	53.4 (44.6-61.9)	38.6 (29.7-47.3)	30.4 (18.0-44.1)
Mozambique	64.3 (52.6-75.2)	61.9 (51.6-71.3)	60.5 (43.8-75.5)	46.9 (35.2-59.0)	44.2 (35.2-53.1)	43.1 (27.1-58.8)
Rwanda	10.8 (7.5-15.0)	57.2 (45.8-67.2)	87.3 (76.8-94.0)	7.8 (5.2-10.9)	48.3 (37.3-58.8)	82.8 (69.5-91.5)
Tanzania	68 (55.1-79.4)	61.9 (52.3-71.0)	57.8 (39.3-74.5)	51.8 (37.8-65.0)	45.0 (35.6-55.2)	41.2 (23.9-60.4)
Uganda	49.9 (39.9-60.3)	65.0 (54.4-75.3)	73.7 (57.0-86.8)	40.1 (30.9-50.4)	55.6 (43.9-66.9)	65.5 (46.3-81.3)
Zambia	60.8 (50.0-71.3)	51.8 (41.4-62.4)	46.2 (29.9-63.5)	62.0 (50.8-72.4)	53.2 (42.9-62.9)	47.6 (31.0-63.9)
Zimbabwe	56.8 (41.2-71.0)	70.4 (61.1-78.8)	77.9 (60.0-90.4)	54.8 (41.0-68.5)	68.6 (59.2-77.0)	76.4 (57.7-89.5)

**West and Central Africa**						
Benin	64.1 (49.3-76.3)	54.8 (45.3-64.1)	51.3 (32.8-70.1)	51.7 (37.8-64.9)	42.0 (34.0-49.9)	38.9 (22.8-57.4)
Burkina Faso	23.8 (14.4-34.7)	62.3 (43.6-78.9)	82.1 (58.0-95.6)	12.3 (7.3-18.5)	43.1 (26.7-61.4)	68.6 (38.8-90.5)
Cameroon	70.2 (56.8-81.9)	68.5 (56.0-79.2)	67.3 (42.5-85.5)	46.9 (33.1-61.3)	44.8 (33.2-56.3)	44.4 (23.0-67.4)
Central African Republic	43.5 (31.3-55.2)	51.6 (33.1-68.8)	57.4 (28.2-82.1)	20.2 (13.6-28.6)	26.4 (14.8-40.7)	32.4 (12.2-58.7)
Chad	24.9 (15.8-36.0)	61.4 (47.9-73.3)	81.4 (63.6-92.6)	8.5 (4.9-13.4)	31.0 (21.7-41.9)	56.3 (33.7-77.8)
Congo	75.4 (63.5-85.4)	83.8 (76.3-90.2)	87.7 (74.4-95.9)	59.0 (43.7-72.2)	70.8 (60.0-79.7)	77.4 (57.7-91.0)
Cote d'Ivoire	40.0 (8.7-81.3)	58.9 (42.6-74.9)	69.8 (33.8-93.3)	27.5 (4.7-68.6)	43.0 (30.7-56.3)	56.7 (21.8-87.0)
Democratic Republic of the Congo	44.5 (30.4-59.3)	55.3 (44.9-64.2)	63.3 (44.6-79.1)	32.3 (21.2-45.0)	42.3 (33.3-51.8)	51.0 (32.5-70.6)
Ghana	72.0 (62.3-80.5)	87.6 (80.3-92.4)	92.9 (84.3-97.3)	56.6 (45.4-66.5)	78.1 (68.7-86.1)	87.0 (73.2-95.1)
Guinea	72.8 (59.0-82.8)	62.1 (53.1-71.0)	54.0 (36.6-71.7)	48.9 (34.6-62.9)	36.5 (28.5-45.8)	29.7 (16.5-46.6)
Liberia	57.3 (38.4-75.3)	86.8 (80.6-91.3)	94.7 (87.6-98.2)	41.3 (24.4-60.1)	77.2 (67.7-84.9)	90.3 (78.5-96.6)
Mali	47.9 (37.2-58.5)	62.9 (54.5-70.5)	72.4 (59.1-83.0)	22.8 (15.8-30.7)	35.1 (27.6-42.9)	45.9 (31.7-60.3)
Niger	17.9 (8.7-30.6)	60.8 (42.3-77.4)	83.8 (58.7-96.4)	8.2 (3.8-15.0)	39.1 (22.9-55.9)	69.4 (35.7-91.5)
Nigeria	55.4 (45.9-65.5)	62.8 (55.8-70.2)	67.7 (54.4-79.4)	25.4 (18.6-32.9)	31.5 (24.9-38.3)	36.8 (24.6-51.0)
Sao Tome and Principe	66.9 (43.4-84.9)	86.3 (77.3-92.7)	91.9 (76.8-98.6)	60.1 (35.5-80.7)	82.2 (71.7-90.3)	89.5 (70.6-97.9)
Senegal	42.2 (29.5-55.8)	61.2 (53.7-68.1)	72.5 (57.6-84.2)	27.7 (17.4-39.6)	45.1 (37.5-53.4)	58.2 (41.8-74.1)
Sierra Leone	52.8 (35.5-69.6)	82.7 (75.5-88.4)	92.2 (83.1-97.4)	46.8 (28.8-63.7)	78.9 (70.3-85.4)	90.3 (79.4-96.4)
The Gambia	59.5 (45.3-73.0)	74.1 (65.3-81.0)	81.5 (66.1-91.1)	61.8 (47.4-74.8)	75.9 (68.1-82.8)	82.8 (68.6-91.9)
Togo	51.9 (27.3-76.8)	74.9 (61.0-85.4)	83.8 (55.8-96.5)	14.7 (4.9-32.1)	31.1 (17.6-46.3)	48.0 (14.9-80.2)
**Latin America and Caribbean**						
Dominican Republic	84.4 (71.1-92.9)	94.8 (90.0-97.5)	97.3 (91.4-99.5)	82.8 (69.0-92.2)	94.2 (89.2-97.2)	97.0 (89.9-99.5)
Haiti	46.5 (30.0-62.0)	62.8 (51.3-73.8)	73.2 (51.9-88.6)	39.0 (24.3-54.8)	55.3 (43.4-66.3)	67.0 (44.4-84.3)
Honduras	84.4 (71.7-92.0)	87.3 (77.6-94.0)	87.7 (66.8-97.5)	78.2 (64.5-88.1)	82.0 (68.9-91.3)	82.8 (56.7-96.6)
**Central and Eastern Europe**						
Albania	67.2 (48.3-83.0)	70.9 (59.5-80.1)	73.5 (52.3-87.9)	67.2 (49.1-82.4)	71.0 (61.4-78.6)	73.7 (53.9-87.4)
Armenia	81.3 (71.0-89.2)	97.2 (95.7-98.4)	99.3 (98.3-99.8)	61.7 (47.6-74.3)	92.8 (89.5-95.3)	98.1 (95.8-99.3)
Kyrgyzstan	98.5 (96.1-99.5)	99.7 (99.5-99.8)	99.9 (99.6-100.0)	65.4 (38.9-85.3)	88.9 (84.3-92.4)	95.2 (88.1-98.6)
Tajikistan	61.8 (34.1-84.7)	79.9 (71.1-87.4)	87.1 (71.7-96.5)	42.3 (18.6-68.9)	63.1 (50.2-74.5)	74.9 (49.4-91.7)

LMICs, low-and middle-income countries; CrI, credible interval

Table S6: Rate of changes for coverage of single and four ANC visits between 2000 and 2030

Country	Rate of change for ANC1			Rate of change for ANC4		
	**National**	**Urban**	Rural	**National**	**Urban**	Rural
**South Asia**						
Afghanistan	77.43	75.80	79.66	90.41	89.51	90.02
Bangladesh	49.06	47.95	55.50	70.12	69.07	74.23
India	23.81	26.10	36.92	68.63	70.19	78.97
Maldives	-6.16	-8.47	-17.00	-2.02	-2.02	-2.62
Nepal	71.50	76.98	87.57	89.20	90.80	95.97
Pakistan	53.37	54.64	64.92	80.91	79.42	83.38
**East Asia and the Pacific**						
Cambodia	56.06	55.26	65.93	89.11	88.31	92.60
Indonesia	13.37	10.51	13.17	28.16	21.93	26.28
Laos	75.88	75.40	82.34	91.93	90.14	92.22
Myanmar	40.25	38.41	42.33	50.38	46.00	46.79
Papua New Guinea	35.50	36.83	41.82	42.76	42.57	46.07
Philippines	13.32	12.16	16.08	36.04	32.02	38.80
Timor-Leste	-1.71	-1.81	-2.06	75.40	72.58	77.37
Vietnam	35.78	22.63	20.99	80.89	71.49	69.02
**Eastern and Southern Africa**						
Angola	-22.14	-20.42	-25.95	61.31	58.31	61.71
Burundi	19.30	16.10	21.50	78.29	76.02	78.83
Comoros	24.16	21.68	27.39	60.40	56.25	58.40
Ethiopia	71.79	71.70	77.97	85.48	84.80	87.58
Kenya	20.55	18.10	24.57	42.10	37.38	41.04
Lesotho	11.09	11.71	17.71	21.55	18.47	20.38
Madagascar	23.59	19.40	22.73	56.75	52.39	55.05
Malawi	7.68	8.28	12.61	-38.71	-36.68	-40.40
Mozambique	11.41	11.94	17.53	-8.84	-8.27	-9.09
Rwanda	10.71	9.51	12.71	90.73	88.96	90.33
Tanzania	9.97	8.75	11.40	5.53	5.08	5.63
Uganda	6.92	6.50	9.20	47.86	46.49	51.02
Zambia	7.12	7.02	10.37	-24.85	-21.55	-22.97
Zimbabwe	5.84	5.50	7.35	36.28	32.77	35.52
**West and Central Africa**						
Benin	4.24	3.80	4.80	-35.22	-31.36	-35.31
Burkina Faso	40.44	38.54	46.95	84.32	82.48	85.00
Cameroon	13.63	11.99	14.93	-1.82	-1.66	-1.78
Central African Republic	21.48	20.60	26.10	32.04	30.00	32.04
Chad	53.88	51.79	57.47	80.81	79.39	81.24
Congo	24.09	21.13	25.76	30.59	26.57	29.23
Cote d'Ivoire	23.14	21.87	28.00	52.78	50.15	52.94
DRC	14.19	12.85	16.00	8.97	8.15	9.05
Ghana	8.85	9.06	13.54	42.18	34.13	36.72
Guinea	4.24	4.12	5.84	-26.37	-24.43	-26.50
Liberia	55.86	55.26	63.86	55.16	51.49	54.45
Mali	36.02	34.69	41.48	46.08	44.32	46.41
Niger	65.04	60.79	65.72	91.42	89.75	90.23
Nigeria	32.88	26.85	28.28	25.84	21.24	21.62
Sao Tome and Principe	4.80	8.31	21.34	40.84	38.15	44.95
Senegal	19.98	18.04	23.33	52.49	49.63	53.34
Sierra Leone	31.96	31.16	39.58	53.06	50.49	54.84
The Gambia	3.61	2.91	3.51	18.97	15.64	15.83
Togo	-152.01	-132.47	-166.15	47.17	43.90	47.26
**Latin America and Caribbean**						
Dominican Republic	47.30	43.30	47.70	20.02	16.70	20.85
Haiti	16.09	15.66	21.86	49.54	43.47	46.84
Honduras	16.10	15.18	20.46	-29.59	-25.27	-32.47
**Central and Eastern Europe**						
Albania	-73.25	-63.70	-87.67	27.70	24.79	28.82

Armenia	18.50	17.40	20.20	43.73	37.98	33.57
Kyrgyzstan	21.50	18.40	22.00	18.90	14.57	17.98
Tajikistan	21.30	24.79	37.22	64.40	65.81	73.03

ANC, antenatal care; ANC1, single antenatal visit; ANC4, four antenatal visits

Table S7: Coverage of at least single antenatal visit among adolescent mothers according to wealth quintile in LMICs, 2000-2030

Country	Predicted coverage, proportion (95% CrI)					
	2000		2018		2030	
	Q1	Q5	Q1	Q5	Q1	Q5
**South Asia**						
Afghanistan	10.9 (0.8-40.9)	43.1 (4.9-87.0)	45.0 (20.8-69.8)	85.8 (64.3-96.3)	73.1 (19.4-98.7)	93.6 (62.6-99.9)
Bangladesh	22.1 (9.3-40.0)	74.6 (53.6-90.2)	49.0 (28.7-69.5)	91.0 (79.6-97)	66.7 (25.6-93.0)	94.7 (78.2-99.4)
India	56.3 (23.7-84.7)	89.3 (69.0-97.9)	73.8 (50.8-89.7)	95.2 (86.7-98.8)	79.7 (40.4-97.8)	96.0 (82.2-99.7)
Maldives	99.4 (97.3-99.9)	99.9 (99.6-100.0)	99.6 (98.9-99.9)	99.9 (99.7-100.0)	99.3 (96.4-100.0)	99.9 (99.2-100.0)
Nepal	16.3 (7.1-30.9)	57.9 (34.0-78.9)	74.4 (57.4-87.9)	95.5 (90.3-98.4)	94.1 (82.2-98.9)	99.2 (97.2-99.9)
Pakistan	32.6 (10.0-65.6)	70.2 (36.5-93.8)	75.5 (56.3-89.0)	94.4 (86.8-98.2)	89.9 (65.9-98.6)	97.9 (92.3-99.8)
**East Asia and the Pacific**						
Cambodia	22.8 (11.2-39.2)	84.4 (69.3-94.1)	98.0 (95.4-99.3)	99.9 (99.7-100.0)	99.9 (99.7-100.0)	100.0 (100.0-100.0)
Indonesia	85.9 (70.9-95.1)	98.9 (97.1-99.7)	89.4 (78.7-96.0)	99.2 (97.8-99.7)	89.7 (67.6-98.3)	99.2 (96.3-99.9)
Laos	10.1 (4.2-19.4)	45.8 (23.4-69.4)	65.3 (44.8-82.6)	93.3 (85.6-97.7)	91.7 (77.2-98.4)	98.8 (96.0-99.8)
Myanmar	42.6 (2.1-94.6)	90.2 (41.0-99.9)	77.1 (54.4-92.3)	99.1 (97.3-99.9)	85.5 (37.6-99.6)	99.3 (95.2-100.0)
Papua New Guinea	39.7 (1.2-95.2)	61.9 (3.8-99.0)	70.0 (46.0-88.6)	89.5 (69.7-97.7)	81.5 (32.1-99.1)	93.4 (65.1-99.8)
Philippines	85.7 (69.2-95.0)	99.7 (99.1-99.9)	84.3 (69.9-93.4)	99.6 (99.2-99.9)	81.6 (50.9-96.4)	99.5 (98.2-99.9)
Timor-Leste	63.1 (19.8-94.5)	87.0 (52.6-99.1)	77.0 (54.0-92.4)	94.1 (84.2-98.5)	79.1 (27.9-99.0)	93.7 (67.7-99.8)
Vietnam	42.8 (24.3-62.5)	99.4 (98.6-99.8)	95.4 (89.5-98.5)	100.0 (99.9-100.0)	99.4 (97.8-99.9)	100.0 (100.0-100.0)
**Eastern and Southern Africa**						
Angola	81.2 (57.1-95.4)	96.3 (87.5-99.4)	70.7 (48.6-86.5)	93.8 (83.4-98.2)	60.1 (15.9-93.2)	88.2 (53.4-99.0)
Burundi	79.6 (64.5-89.8)	87.2 (73.9-95.0)	98.8 (97.5-99.5)	99.3 (98.3-99.8)	99.8 (99.4-100.0)	99.9 (99.6-100.0)
Comoros	57.3 (34.2-78.1)	87.1 (71.4-95.9)	88.1 (70.6-97.1)	97.3 (90.8-99.6)	94.5 (74.0-99.7)	98.7 (92.1-99.9)
Ethiopia	10.1 (4.2-19.2)	45.2 (23.1-68.7)	50.1 (28.9-70.1)	87.5 (72.2-95.7)	79.4 (48.9-95.3)	96.2 (86.3-99.5)
Kenya	61.9 (42.7-78.9)	85.1 (72.1-93.2)	95.7 (91.6-98.3)	98.7 (97.0-99.6)	99.2 (97.4-99.9)	99.8 (99.1-100.0)
Lesotho	74.3 (48.6-91.2)	88.1 (70.4-97.1)	96.5 (90.9-99.0)	98.7 (96.2-99.7)	98.8 (93.7-99.9)	99.6 (97.8-100.0)
Madagascar	55.5 (35.3-74.5)	90.8 (81.4-96.5)	77.4 (60.9-89.2)	96.5 (92.8-98.6)	86.5 (64.3-97.0)	98.0 (93.7-99.7)
Malawi	82.4 (70.4-90.7)	94.9 (90.4-97.8)	97.5 (95.5-98.7)	99.4 (98.7-99.7)	99.3 (98.1-99.8)	99.8 (99.5-100.0)
Mozambique	80.1 (61.7-92.5)	96.8 (92.3-99.1)	86.8 (75.3-94.0)	98.1 (95.7-99.3)	88.9 (70.4-97.7)	98.4 (94.2-99.7)
Rwanda	86.9 (74.6-94.3)	82.9 (66.7-93.4)	99.8 (99.5-99.9)	99.7 (99.3-99.9)	100.0 (99.9-100.0)	100.0 (99.9-100.0)
Tanzania	86.5 (68.5-95.8)	94.9 (86.2-98.7)	97.0 (94.3-98.7)	99.0 (97.6-99.7)	98.7 (95.6-99.8)	99.5 (98.2-99.9)
Uganda	93.4 (86.7-97.3)	93.0 (85.8-97.3)	95.9 (92.0-98.3)	95.7 (91.2-98.3)	96.6 (89.6-99.3)	96.4 (89.0-99.3)
Zambia	83.4 (66.7-93.6)	98.4 (95.7-99.5)	93.3 (86.2-97.2)	99.4 (98.7-99.8)	95.9 (87.3-99.3)	99.7 (98.8-99.9)
Zimbabwe	80.9 (57.4-94.7)	90.7 (75.9-97.8)	91.8 (83.3-96.8)	96.3 (91.6-98.8)	94.0 (76.0-99.5)	97.3 (88.0-99.8)
**West and Central Africa**						
Benin	64.0 (36.6-86.5)	95.1 (87.5-99.0)	67.7 (50.9-81.8)	96.1 (91.2-98.5)	70.0 (33.3-93.5)	95.9 (85.1-99.5)
Burkina Faso	46.5 (23.9-72.1)	92.8 (82.3-98.1)	97.8 (92.4-99.6)	99.9 (99.5-100.0)	99.7 (97.8-100.0)	100.0 (99.9-100.0)

Cameroon	54.7 (35.8-73.3)	94.3 (87.5-98.0)	82.9 (67.4-92.7)	98.5 (96.1-99.6)	91.4 (72.4-98.6)	99.3 (97.0-99.9)
Central African Republic	40.8 (21.3-61.4)	87.1 (72.9-95.2)	63.2 (30.8-88.6)	93.9 (81.5-98.9)	73.0 (22.9-97.8)	95.2 (74.2-99.8)
Chad	16.5 (7.7-29.6)	58.7 (36.1-79.2)	49.9 (27.4-71.5)	87.4 (73.6-95.8)	72.8 (34.8-93.5)	94.5 (81.1-99.2)
Congo	59.7 (30.6-84.2)	94.4 (82.7-98.9)	92.4 (84.2-97.4)	99.4 (98.2-99.8)	97.4 (89.6-99.8)	99.8 (99.0-100.0)
Cote d'Ivoire	47.8 (16.5-78.0)	92.7 (78.4-98.5)	95.4 (88.8-98.4)	99.7 (99.1-99.9)	99.2 (96.5-99.9)	99.9 (99.8-100.0)
Democratic Republic of the Congo	62.7 (42.0-81.3)	91.9 (83.2-96.9)	76.5 (59.6-87.5)	95.7 (90.6-98.4)	82.7 (57.9-95.6)	96.9 (89.3-99.4)
Ghana	82.6 (66.6-93.0)	98.8 (97.0-99.6)	98.7 (96.7-99.6)	99.9 (99.8-100.0)	99.7 (98.7-100.0)	100.0 (99.9-100.0)
Guinea	71.7 (45.9-89.5)	95.8 (89.1-99.0)	74.2 (57.7-86.6)	96.5 (92.3-98.7)	73.9 (41.9-93.7)	96.0 (86.9-99.4)
Liberia	23.7 (4.4-57.8)	76.6 (41.1-95.8)	97.2 (93.1-99.2)	99.8 (99.5-100.0)	99.8 (99.1-100.0)	100.0 (100.0-100.0)
Mali	38.6 (19.4-62.5)	79.4 (60.2-92.3)	69.3 (51.8-83.3)	93.6 (87.7-97.3)	83.6 (60.0-95.5)	97.0 (90.9-99.3)
Niger	21.4 (8.9-41.4)	64.6 (39.9-84.4)	81.0 (59.1-94.3)	96.6 (89.6-99.3)	95.4 (78.5-99.7)	99.2 (96.0-100.0)
Nigeria	21.5 (10.2-37.5)	78.3 (59.3-90.3)	38.6 (23.2-55.1)	89.5 (81.2-95.1)	52.7 (23.5-79.6)	93.2 (82.3-98.4)
Sao Tome and Principe	94.3 (86.9-98.0)	99.7 (99.1-99.9)	99.7 (99.3-99.9)	100.0 (100.0-100.0)	100.0 (99.8-100.0)	100.0 (100.0-100.0)
Senegal	53.4 (34.1-71.0)	96.8 (93.5-98.7)	95.3 (91.8-97.5)	99.8 (99.7-99.9)	99.2 (98.1-99.8)	100.0 (99.9-100.0)
Sierra Leone	55.2 (35.4-75.4)	78.6 (60.0-89.9)	97.5 (94.8-99.0)	99.2 (98.3-99.7)	99.7 (99.0-100.0)	99.9 (99.7-100.0)
The Gambia	92.1 (80.7-97.9)	98.8 (96.3-99.7)	99.0 (97.9-99.6)	99.9 (99.7-100.0)	99.7 (98.8-100.0)	100.0 (99.8-100.0)
Togo	76.1 (48.7-92.8)	99.5 (98.5-99.9)	35.7 (18.0-56.3)	97.3 (93.6-99.1)	16.7 (2.5-48.8)	89.7 (63.8-98.7)
**Latin America and Caribbean**						
Dominican Republic	60.6 (22.8-91.0)	92.5 (74.3-99.2)	99.7 (99.0-99.9)	100.0 (99.9-100.0)	100.0 (99.9-100.0)	100.0 (100.0-100.0)
Haiti	59.9 (28.4-86.2)	87.6 (65.8-97.5)	88.3 (75.6-95.4)	97.5 (93.9-99.3)	94.6 (79.8-99.5)	98.9 (94.9-99.9)
Honduras	73.8 (40.3-94.0)	93.4 (79.0-98.9)	95.7 (86.0-99.2)	99.2 (96.9-99.9)	97.7 (86.2-100.0)	99.5 (96.9-100.0)
**Central and Eastern Europe**						
Albania	98.6 (95.1-99.8)	99.9 (99.7-100.0)	94.6 (88.4-98.0)	99.7 (99.3-99.9)	83.5 (47.6-98.0)	98.8 (94.3-99.9)
Armenia	95.6 (87.8-98.9)	99.5 (98.5-99.9)	99.7 (99.4-99.9)	100.0 (99.9-100.0)	99.9 (99.8-100.0)	100.0 (100.0-100.0)
Kyrgyzstan	94.5 (85.2-98.7)	98.8 (96.2-99.7)	99.4 (98.8-99.8)	99.9 (99.7-100.0)	99.8 (99.4-100.0)	100.0 (99.9-100.0)
Tajikistan	67.1 (44.6-83.3)	89.2 (76.8-96.1)	84.6 (71.4-93.7)	95.8 (89.8-98.6)	90.7 (72.6-98.1)	97.5 (91.6-99.6)

Q1, Poorest wealth quintile; Q5, Richest wealth quintile; CrI, credible intervals

Table S8: Coverage of at least four antenatal visits among adolescent mothers according to wealth quintile in LMICs, 2000-2030

Country	Predicted coverage, proportion (95% CrI)					
	2000		2018		2030	
	Q1	Q5	Q1	Q5	Q1	Q5
**South Asia**						
Afghanistan	2.1 (0.1-9.6)	10.1 (1.0-36.5)	11.9 (3.9-25.3)	43.4 (22.9-65.7)	39.9 (4.2-87.0)	72.4 (22.3-98.0)
Bangladesh	6.0 (2.3-12.4)	28.3 (14.0-46.3)	15.4 (7.1-27.9)	52.7 (35.1-69.5)	28.5 (7.2-61.9)	67.8 (34.2-90.7)
India	15.7 (4.3-35.0)	52.3 (24.8-78.1)	37.8 (17.9-61.6)	78.9 (60.9-90.8)	57.1 (19.1-88.5)	87.8 (62.1-98.1)
Maldives	69.6 (35.3-92.2)	99.5 (98.3-99.9)	79.7 (60.4-91.7)	99.7 (99.3-99.9)	81.8 (46.1-97.4)	99.7 (98.7-100.0)
Nepal	5.0 (2.2-9.8)	25.7 (14.0-40.9)	43.4 (27.1-60.2)	83.3 (72.4-91.2)	81.2 (58.0-93.6)	96.5 (90.6-99.1)
Pakistan	7.4 (1.9-18.3)	40.4 (17.3-68.3)	24.8 (12.5-41.8)	74.5 (58.3-86.5)	47.0 (17.3-78.9)	87.4 (67.2-97.5)
**East Asia and the Pacific**						
Cambodia	5.2 (2.3-9.6)	28.3 (16.5-42.8)	77.4 (60.0-89.7)	96.1 (91.9-98.5)	97.9 (93.4-99.7)	99.7 (99.0-100.0)
Indonesia	54.3 (34.5-73.4)	88.8 (78.5-95.1)	73.8 (57.0-86.7)	95.1 (90.8-97.9)	82.2 (57.4-95.8)	96.9 (91.1-99.3)
Laos	4.0 (0.2-17.2)	21.5 (2.4-61.9)	30.4 (14.0-51.5)	76.6 (58.8-89.5)	69.1 (24.8-96.2)	93.2 (72.7-99.5)
Myanmar	18.6 (0.7-73.5)	48.5 (4.4-94.3)	33.7 (13.3-57.1)	75.2 (51.7-89.6)	52.3 (7.7-94.5)	82.7 (36.0-99.0)
Papua New Guinea	22.3 (0.7-82.3)	41.4 (2.4-94.1)	38.0 (17.4-62.1)	66.9 (42.5-84.5)	56.3 (10.1-92.5)	78.2 (32.0-97.8)
Philippines	45.0 (25.8-64.6)	88.7 (78.0-95.1)	66.9 (47.1-81.8)	95.3 (90.8-97.8)	77.5 (47.2-93.9)	97.0 (91.2-99.3)
Timor-Leste	19.2 (3.2-51.7)	41.6 (11.4-79.4)	66.7 (43.4-85.1)	87.2 (74.6-94.8)	87.5 (56.7-98.9)	95.7 (81.6-99.7)
Vietnam	3.5 (1.1-7.9)	83.1 (67.3-93.1)	60.7 (37.1-80.6)	99.6 (98.9-99.9)	93.9 (79.5-99.2)	100.0 (99.8-100.0)
**Eastern and Southern Africa**						
Angola	33.6 (9.5-65.2)	73.4 (40.6-93.1)	44.4 (23.3-67.4)	83.4 (71.1-92.0)	52.4 (14.6-87.8)	85.4 (57.0-97.8)
Burundi	12.8 (2.0-37.8)	30.2 (6.8-63.4)	41.8 (22.2-64.4)	69.9 (49.7-84.3)	67.4 (25.7-94.8)	85.6 (54.9-98.1)
Comoros	13.7 (0.9-50.5)	36.6 (5.1-80.8)	33.5 (9.3-66.0)	67.5 (36.6-90.1)	53.8 (5.2-97.3)	77.7 (18.2-99.4)
Ethiopia	2.4 (1.1-5.0)	18.1 (9.6-31.3)	15.1 (7.2-27.2)	60.3 (41.7-78.3)	39.9 (14.4-70.3)	83.5 (61.5-95.9)
Kenya	29.0 (13.6-48.7)	47.1 (27.3-67.5)	44.5 (25.6-63.7)	63.8 (45.0-80.7)	55.4 (21.4-86.7)	71.9 (36.3-93.6)
Lesotho	50.6 (27.9-75.4)	73.6 (55.0-87.9)	65.0 (42.1-83.9)	83.4 (66.1-94.1)	71.5 (31.8-94.7)	86.0 (53.9-98.3)
Madagascar	19.1 (6.2-43.2)	50.1 (25.9-74.3)	45.2 (14.1-78.9)	76.4 (41.6-94.9)	62.0 (10.4-97.0)	83.6 (32.9-99.4)
Malawi	46.8 (31.4-63.1)	63.1 (48.4-76.7)	36.6 (21.4-52.6)	52.8 (36.2-69.3)	31.3 (11.6-55.8)	46.0 (20.4-73.2)
Mozambique	36.3 (17.8-57.5)	69.1 (48.9-84.9)	36.2 (21.0-52.8)	69.6 (53.5-82.4)	37.0 (14.2-65.6)	68.8 (39.1-89.3)
Rwanda	5.9 (2.6-10.5)	10.9 (5.6-18.7)	43.8 (25.0-63.8)	60.0 (40.5-77.1)	79.0 (49.1-94.9)	87.6 (66.6-97.2)
Tanzania	44.2 (22.1-66.8)	66.9 (45.7-84.7)	42.0 (25.1-59.8)	65.4 (47.3-79.9)	41.2 (15.7-71.1)	63.3 (32.1-86.7)
Uganda	33.4 (18.1-51.4)	48.4 (31.2-65.4)	54.5 (35.0-73.1)	69.1 (51.8-83.6)	67.3 (36.6-89.5)	78.9 (52.4-94.3)
Zambia	58.4 (38.9-76.8)	65.4 (46.9-81.5)	50.0 (30.4-67.9)	57.4 (39.1-72.8)	44.7 (17.5-72.8)	51.7 (23.3-77.1)
Zimbabwe	47.6 (22.7-71.8)	65.0 (39.8-84.1)	64.7 (45.7-80.8)	79.4 (64.8-89.8)	73.0 (38.2-94.0)	84.2 (58.8-97.0)
**West and Central Africa**						
Benin	40.7 (17.7-66.9)	78.5 (60.0-91.7)	31.0 (17.8-47.8)	71.8 (58.3-82.9)	27.6 (7.9-59.4)	65.3 (33.7-89.5)
Burkina Faso	7.1 (2.4-16.0)	28.3 (12.3-48.0)	36.1 (11.7-68.3)	72.7 (43.4-91.7)	63.8 (13.9-96.0)	87.4 (49.5-99.3)

Cameroon	35 (16.4-58.4)	77.4 (58.0-90.3)	38.8 (20.3-61.8)	80.3 (64.4-91.1)	42.4 (11.0-79.9)	80.1 (47.7-96.3)
Central African Republic	15.2 (6.1-28.3)	48.7 (29.5-67.9)	25.1 (7.6-53.1)	62.3 (32.5-86.2)	34.9 (5.5-79.9)	68.6 (24.7-95.6)
Chad	0.2 (0.1-0.7)	6.9 (2.4-15.8)	15.1 (5.3-31.1)	83.1 (69.7-93.0)	71.8 (32.7-94.9)	98.5 (94.8-99.8)
Congo	43.9 (20.4-69.1)	83.4 (66.0-93.7)	69.0 (49.1-85.7)	93.8 (87.5-97.3)	79.8 (48.0-96.5)	96.1 (85.3-99.4)
Cote d'Ivoire	19.2 (0.9-75.2)	45.9 (4.3-94.0)	37.5 (16.3-63.1)	75.4 (56.0-89.1)	57.3 (9.0-95.8)	83.7 (37.3-99.0)
Democratic Republic of the Congo	24.2 (9.2-46.3)	52.5 (28.2-76.2)	37.7 (22.2-55.2)	68.6 (52.9-81.6)	49.0 (19.2-80.5)	76.0 (45.6-94.1)
Ghana	30.5 (14.8-48.4)	94.8 (89.8-97.7)	84.9 (71.1-93.9)	99.6 (99.1-99.9)	96.3 (87.7-99.5)	99.9 (99.7-100.0)
Guinea	35.9 (15.8-60.5)	76.5 (57.5-90.9)	26.6 (14.5-41.4)	69.0 (54.2-81.5)	22.8 (6.9-48.6)	62.1 (32.8-86.1)
Liberia	28.2 (8.1-56.5)	59.2 (26.9-83.8)	72.7 (52.9-87.5)	91.5 (82.4-96.5)	88.8 (65.9-98.5)	96.8 (87.2-99.6)
Mali	16.1 (7.5-29.0)	47.9 (30.9-65.7)	25.8 (15.2-40.2)	62.9 (46.9-76.6)	35.2 (15.3-60.7)	71.2 (46.3-88.7)
Niger	5.6 (1.2-15.2)	16.6 (4.9-36.6)	32.7 (10.6-60.0)	61.1 (27.3-86.5)	63.1 (13.5-96.2)	81.9 (31.4-99.0)
Nigeria	12.8 (6.2-22.4)	66.7 (49.9-80.2)	20.0 (11.7-30.9)	77.6 (65.8-86.4)	27.3 (10.5-49.9)	82.4 (63.4-93.5)
Sao Tome and Principe	42.0 (10.6-77.3)	78.0 (43.9-95.4)	79.6 (55.5-93.3)	95.8 (89.8-98.9)	89.5 (55.8-99.5)	97.9 (88.8-99.9)
Senegal	18.5 (7.7-35.9)	51.7 (29.4-74.1)	37.4 (25.4-51.4)	74.7 (62.4-84.4)	53.7 (27.8-79.5)	84.2 (63.6-95.2)
Sierra Leone	39.3 (16.1-68.5)	57.6 (30.6-81.3)	74.0 (57.1-86.7)	86.2 (73.8-93.7)	86.6 (61.0-97.7)	93.2 (74.7-99.0)
The Gambia	52.3 (27.2-76.0)	68.4 (44.3-86.3)	71.1 (53.8-85.1)	83.4 (72.7-91.2)	79.4 (52.5-95.0)	88.4 (70.1-97.4)
Togo	0.1 (0.0-0.4)	15.5 (1.7-50.0)	6.6 (1.4-18.4)	91.9 (80.5-97.9)	54.0 (6.5-96.2)	99.1 (94.7-100.0)
**Latin America and Caribbean**						
Dominican Republic	55.6 (22.1-84.3)	93.9 (81.2-98.8)	93.8 (83.8-98.4)	99.6 (98.8-99.9)	98.0 (89.2-99.9)	99.9 (99.3-100.0)
Haiti	28.6 (10.2-54.3)	58.9 (30.1-81.6)	47.6 (27.7-68.5)	77.3 (62.0-89.3)	60.7 (25.1-89.8)	84.0 (56.8-97.2)
Honduras	54.3 (23.3-82.6)	87.1 (69.5-96.2)	79.4 (55.4-93.9)	95.8 (88.8-99.0)	86.3 (45.7-99.2)	97.0 (84.6-99.9)
**Central and Eastern Europe**						
Albania	62.0 (29.4-87.1)	90.7 (74.8-97.9)	58.4 (36.0-77.5)	90.2 (81.7-95.4)	55.1 (18.3-87.5)	87.1 (60.5-97.6)
Armenia	29.9 (11.0-55.3)	87.1 (72.2-95.4)	98.0 (95.3-99.3)	99.9 (99.8-100.0)	99.9 (99.6-100.0)	100.0 (100.0-100.0)
Kyrgyzstan	61.2 (21.7-90.2)	95.7 (83.9-99.5)	81.9 (68.5-91.2)	98.8 (97.7-99.5)	87.7 (61.8-98.3)	99.2 (96.9-99.9)
Tajikistan	27.7 (3.9-67.8)	62.6 (19.0-92.7)	49.8 (28.2-72.2)	84.4 (70.3-93.3)	65.0 (21.6-95.2)	89.6 (62.0-99.0)

Q1, Poorest wealth quintile; Q5, Richest wealth quintile; CrI, credible intervals

Table S9: Changes in the magnitude of socio-economic inequality in access to antenatal care, 2000-2030

Country	Slope index of inequality (95% CrI)					
	ANC1			ANC4		
	**2000**	**2018**	**2030**	**2000**	**2018**	**2030**
**South Asia**						
Afghanistan	41.7 (31.5-51.8)	53.7 (41.5-65.8)	27.7 (19.2-36.1)	10.7(2.8-18.5)	36.1(15.9-56.2)	40.2(30.2-50.1)
Bangladesh	66.0 (50.8-81.2)	53.6(47.3-59.9)	36.4(29-43.7)	32.4(22.6-42.1)	36.5(25.2-47.7)	36.9(28.2-45.5)
India	41.3 (35.9-46.6)	27(19.4-34.6)	20.6(15.4-25.8)	47.2(39.8-54.5)	46.7(43.1-50.3)	39.1(35.6-42.6)
Maldives	.6 (.04-1.2)	.4 (.2-.6)	.8(.2-1.4)	66(37.2-94.8)	2(-.4-4.4)	.1(-.1-.3)
Nepal	51.8 (35.3-68.3)	26.4(18.4-34.3)	6.3(3.5-9.1)	26(7.3-44.7)	43.9(20.5-67.2)	46.5(30.4-62.5)
Pakistan	49.3 (44.1-54.5)	24.1(14.7-33.4)	9.6(5.1-14.6)	45.7(40.1-51.2)	48.9(38.7-59.1)	45.5(34.1-56.9)
**East Asia and the Pacific**						
Cambodia	77.0 (57.3-96.6)	2.3(1-3.6)	.1(-.1-.3)	24.6(9.5-39.7)	43.5(34.6-52.4)	28.4(25.8-30.9)
Indonesia	16.5 (6.5-26.4)	12.2(4.3-20.1)	11.7(4.5-18.8)	20.6(7.6-33.6)	33.9(20.7-47)	22.4(17.4-27.4)
Laos	46.2 (32.2-60.2)	35.4(21.6-49.1)	8.6(3.7-13.5)	27.5(7.5-47.4)	23(16-30.1)	2.2(1.1-3.4)
Myanmar	59.4 (42.1-76.8	27.7(16.4-39)	16.7(11.5-21.9)	50.9(46.3-55.6)	50.1(46.2-54)	44.6(40.8-48.4)
Papua New Guinea	28.6 (25.1-32.1)	25.3(19.3-31.3)	15.1(11.2-19.1)	39.5(19.4-59.5)	44(27.7-60.2)	40.3(29.1-51.6)
Philippines	17.4 (5.7-29.1)	18.3(6.1-30.5)	21.3(8.3-34.4)	7.5(1.2-13.8)	78.1(45.9-110.2)	27.8(-7.1-62.6)
Timor-Leste	31.3 (29.6-33)	23.1(19.1-27.1)	20.1(18.1-22.1)	25.4(15.6-35.2)	39.1(31-47.1)	27.1(21.3-32.8)
Vietnam	71.3 (29.8-112.7)	5.6(-.5-11.7)	.8(-.2-1.8)	46.6(41.3-51.8)	29(21.3-36.7)	18.6(13-24.2)
**Eastern and Southern Africa**						
Angola	19.2 (5.8-32.5)	29.6(10.4-48.8)	37.1(17.1-57.1)	32.6(21.4-43.7)	46.9(40.1-53.7)	32.4(28.5-36.2)
Burundi	11.5 (-12.9-35.8)	.8(-.7-2.2)	.1(-.2-.5)	33.5(16.4-50.6)	36.8(22.9-50.8)	32.1(22.7-41.5)
Comoros	37.6 (28.5-46.6)	11.4(6.6-16.2)	4.9(2.9-6.9)	44.4(31.3-57.4)	6.7(2.1-11.3)	2.1(.8-3.4)
Ethiopia	41.3 (12.5-70.1)	46.2(40.3-52.1)	21.5(18.9-24.1)	18.9(1.8-35.9)	53.9(26.5-81.3)	53.6(44.9-62.4)
Kenya	31.7 (17-46.3)	4.3(1.8-6.7)	.7(.2-1.3)	81.3(62.5-100)	18(2.9-33)	4.4(.3-8.4)
Lesotho	18.1 (6.7-29.4)	2.7(.7-4.6)	.9(.4-1.4)	48.4(42.3-54.5)	50.3(40.4-60.2)	46.5(35.1-57.8)
Madagascar	43.3 (41.1-45.5)	23.3(19.6-27)	14.3(11.5-17.1)	36.5(23.4-49.5)	35.6(29.3-42)	27(23.5-30.4)
Malawi	16.6 (8.2-25.5)	2.5(1.1-3.9)	.7(.2-1.1)	40(36.4-43.6)	19.8(14.1-25.2)	12.9(9.7-16.1)
Mozambique	21.1 (15.8-26.40	13.8(9.7-17.9)	11.4(8.2-14.6)	43.9(29-58.8)	49.3(41.5-57)	36.9(27.2-46.7)
Rwanda	-2.9 (-21.6-15.7)	-.1(-.5-.4)	0	40.8(23.6-58)	24.4(9.5-39.2)	16.3(5.6-26.9)
Tanzania	10.3 (8-12.5)	2.3(1.8-2.8)	1.1(.8-1.3)	20.7(10.4-31)	22.2(13.3-31)	18.7(12.3-25)
Uganda	-.3 (-2.4-1.8)	-.3(-1.4-.8)	-.4(-1.3-.6)	41(31.4-50.5)	20.2(9.8-30.5)	13.9(7-20.8)
Zambia	18.5 (8-28.9)	7.3(2.7-11.8)	4.4(1.7-7.1)	21.6(12.5-30.6)	53.6(42.6-64.5)	27.6(13.5-41.7)
Zimbabwe	11.8 (9.7-13.9)	5.6(4.8-6.4)	4.3(3.4-5.1)	28.1(14.7-41.4)	22.9(14.4-31.4)	18.2(11.3-25.1)
**West and Central Africa**						
Benin	40.0 (31.5-48.5)	35.2(25.2-45.2)	32.4(24.6-40.2)	37.5(28.8-46.2)	23.3(19.9-26.6)	10.2(7.9-12.4)
Burkina Faso	58.5 (56.6-60.5)	2.3(1.3-3.3)	.4(.2-.5)	36.8(20.1-53.5)	37(30.4-43..6)	25(21.4-28.6)

Cameroon	49.9 (37.7-62)	19.3(10.2-28.3)	9.8(4.8-14.8)	19.7(10.6-28.7)	19.4(9.3-29.5)	17.8(8.1-27.4)
Central African Republic	57.8 (55.6-60)	36.2(28.8-43.6)	25.2(19.9-30.4)	35.2(3.4-67)	22.8(-.8-46.4)	20.4(.4-40.4)
Chad	52.4 (36.4-68.3)	47(41.9-52)	27.5(20.8-34.2)	38.2(19.3-57.1)	45(28.9-61.1)	44(33-55)
Congo	44.8 (35.7-53.8)	8.7(4.3-13.1)	3.2(1.4-4.9)	40.6(31.3-49.8)	41.2(31.9-50.50	39.1(30.4-47.8)
Cote d'Ivoire	57.8 (43.9-71.7)	5.3(1.7-9)	1(.1-1.8)	34.7(22.1-47.3)	50.3(42.3-58.2)	35.8(30.6-40.9)
Democratic Republic of the Congo	36.9 (29-44.7)	23.5(15.9-31.1)	17.3(11.2-23.3)	24.4(10.7-38)	48.7(40.3-57)	18(9.2-26.7)
Ghana	19.6 (9.1-30)	1.5(.5-2.4)	.4(.01-.7)	12.4(2.5-22.2)	33.9(19.1-48.7)	21.9(16.6-27.1)
Guinea	29.6 (20.2-39)	26.7(16.8-36.5)	26.7(18.3-35.1)	64.8(47.4-82.2)	69.8(62.5-77.2)	66.7(57.7-75.6)
Liberia	66.8 (58.2-75.4)	2.9(.9-4.9)	.1(.04-.3)	40.6(16.1-65)	61.2(49.2-73.2)	49.1(42.7-55.4)
Mali	49.7 (43-563)	29.4(25-33.8)	16.6(12.9-20.2)	22.1(14.3-29.9)	34.7(26.7-42.7)	26.3(21.4-31.2)
Niger	49.8 (-1.6-11.4)	17.4(16-18.9)	4.3(3.8-4.7)	52.6(39.6-65.6)	34.2(17.9-50.6)	23.6(11-36.1)
Nigeria	72.5 (62.3-82.8)	64.9(54.8-75.1)	51.1(37.5-64.7)	5.6(2.2-9.1)	18.6(11.4-25.8)	10(6.4-13.5)
Sao Tome and Principe	6.8 (1.5-12)	.4(.01-.7)	0	45.1(33-57.2)	21.4(19.3-23.5)	11.6(10.3-12.8)
Senegal	55.8 (28.4-83.2)	5.5(.8-10.2)	1(.3-1.6)	40.2(28-52.3)	44.9(39-50.8)	36.7(30.3-43)
Sierra Leone	30.7 (18.9-42.5)	2.1(.9-3.3)	.3(.1-.4)	22.4(11.1-33.7)	15.4(11-19.7)	8.3(6.2-10.5)
The Gambia	8.5 (6-11)	1.2(.8-1.6)	.5(.2-.7)	42.2(30.3-54)	42.5(37.2-47.2)	28.7(23.8-33.6)
Togo	27.4 (15.6-39.2)	76(71.9-80.1)	86.6(36.3-136.9)	27.5(15.7-39.2)	28.4(15.6-41.1)	26.8(14.3-39.2)
**Latin America and Caribbean**						
Dominican Republic	41.2 (28.1-54.4)	.49 (.2-.5)	0	18.8(6.9-30.6)	14.4(6.9-21.8)	10.6(5.3-15.8)
Haiti	36.7 (25.8-47.5)	11.7 (5.8-17.5)	5.4(2.5-8.2)	27.4(12.9-41.9)	24.7(21.2-28.2)	10(8.7-11.3)
Honduras	25.3 (17.1-33.4)	4.2 (2.4-6)	2.1(1.2-3)	18.8(.2-37.4)	104(64.2-143.8)	50(-15.7-115.7)
**Central and Eastern Europe**						
Albania	1.6 (0.7-2.5)	6.1 (2.8-9.4)	19.3 (11.9-26.7)	17.6(3.6-31.5)	17.4(5.9-28.8)	14(5.5-22.4)
Armenia	5.0 (1.7-8.2)	.4 (.01-.7)	.1(-.1-.3)	100.5(62.6-138.3)	43.4(-4.8-91.6)	6.2(-1.9-14.3)
Kyrgyzstan	5.7 (1.8-9.5)	.7 (.2-1.1)	.2(.1-.4)	8.2(-.717.1)	8.6(-1.1-18.2)	8.2(-1.4-17.8)
Tajikistan	28.7 (16.3-41.1)	14.2 (7-21.3)	8.8(4.3-13.3)	22.6(8.6-36.6)	19.1(9.3-28.9)	14.7(7.3-22)

ANC1, at least single antenatal visit; ANC4, at four antenatal visits from skilled health provider; CrI, Credible Intervals

Table S10: Posterior mean difference by considering with and without country level predictors for single ANC visit

Country	Mean difference with and without country level predictors						
	2000	2005	2010	2015	2020	2025	2030
Afghanistan	-0.80	-0.80	-0.10	1.00	1.50	1.30	0.90
Albania	0.30	0.40	0.50	0.50	0.70	0.60	0.50
Angola	0.20	0.40	0.40	0.50	0.40	0.10	-0.30
Armenia	0.30	0.10	0.00	0.00	0.00	0.00	0.00
Bangladesh	-0.50	0.00	0.50	0.90	1.20	1.40	1.50
Benin	0.50	0.60	0.70	0.80	0.70	0.70	0.50
Burkina Faso	1.20	0.20	-0.20	-0.20	-0.20	-0.10	-0.10
Burundi	0.60	0.20	0.00	-0.10	0.00	-0.10	0.00
Cambodia	0.00	0.20	0.20	0.10	0.10	0.00	0.00
Cameroon	-0.10	-0.10	0.00	0.10	0.10	0.20	0.10
Central African Republic	0.60	0.50	0.20	0.10	0.00	-0.10	-0.20
Chad	-0.10	0.10	0.30	0.50	0.70	0.70	0.70
Comoros	0.30	0.20	0.00	-0.10	-0.10	-0.20	-0.20
Congo	0.20	0.20	0.20	0.20	0.20	0.20	0.30
Cote d'Ivoire	0.30	0.20	0.20	0.20	0.10	0.10	0.10
Democratic Republic of the Congo	-0.30	-0.20	-0.10	-0.20	-0.10	-0.30	-0.40
Dominican Republic	-0.40	-0.10	0.00	0.00	0.00	0.00	0.00
Ethiopia	0.10	0.20	0.50	0.60	0.60	0.50	0.40
Ghana	-0.30	-0.10	0.00	0.00	0.00	0.00	0.10
Guinea	-0.10	-0.10	-0.20	-0.20	-0.10	-0.20	-0.20
Haiti	0.50	0.20	-0.10	-0.30	-0.50	-0.60	-0.70
Honduras	-0.50	-0.30	-0.10	-0.10	0.00	0.00	-0.10
India	-0.40	-0.50	-0.40	-0.40	-0.40	-0.50	-0.60
Indonesia	-0.10	-0.20	-0.20	-0.20	-0.30	-0.20	-0.20
Kenya	-0.10	0.00	-0.10	0.00	-0.10	-0.10	-0.10
Kyrgyzstan	0.30	0.00	-0.10	-0.10	0.00	0.00	0.00
Laos	0.10	0.10	0.20	0.20	0.20	0.10	0.00
Lesotho	0.30	0.10	0.00	-0.10	-0.10	-0.10	-0.10
Liberia	-1.80	-0.90	-0.10	0.10	0.00	0.10	0.00
Madagascar	0.90	0.40	0.00	-0.20	-0.40	-0.50	-0.60
Malawi	-0.10	-0.10	0.00	0.00	0.00	0.00	0.00
Maldives	0.00	0.00	0.00	0.00	0.00	0.10	0.30
Mali	-0.40	-0.20	0.00	0.20	0.20	0.30	0.30
Mozambique	0.10	0.20	0.10	0.10	0.20	0.10	0.10
Myanmar	0.70	0.40	0.20	0.00	-0.20	-0.60	-1.00
Nepal	0.00	0.10	0.30	0.20	0.20	0.10	0.10
Niger	1.50	0.90	0.10	-0.40	-0.50	-0.40	-0.40
Nigeria	-0.30	-0.30	-0.40	-0.60	-0.60	-0.70	-0.90
Pakistan	-0.10	-0.30	-0.50	-0.60	-0.50	-0.40	-0.30
Papua New Guinea	5.00	4.10	2.40	0.40	-1.10	-2.00	-2.50
Philippines	-0.10	-0.10	-0.10	0.00	0.00	0.00	0.00
Rwanda	-0.10	0.00	-0.10	0.00	0.00	0.00	0.00
Sao Tome and Principe	0.00	0.00	0.00	0.00	0.00	0.00	0.00
Senegal	0.10	0.10	0.00	0.00	-0.10	0.00	0.00
Sierra Leone	0.00	0.10	0.10	0.10	0.00	0.10	0.00

Tajikistan	-0.40	-0.10	-0.10	0.00	0.00	-0.10	-0.10
Tanzania	0.10	0.10	0.00	-0.10	0.00	0.00	0.00
The Gambia	-0.10	0.00	0.00	0.00	-0.10	0.00	0.00
Timor-Leste	-1.00	-0.40	0.00	0.30	0.60	0.50	0.50
Togo	-0.30	-0.30	-0.20	-0.10	0.10	0.10	0.20
Uganda	0.00	0.00	0.00	0.00	0.00	0.10	0.00
Vietnam	-0.40	-0.30	-0.20	-0.10	0.00	-0.10	0.00
Zambia	-0.10	0.00	0.00	0.00	0.00	0.00	0.00
Zimbabwe	0.00	0.00	-0.20	-0.30	-0.40	-0.60	-0.60
All countries (median)	0.00	0.00	0.00	0.00	0.00	0.00	0.00
All countries (mean)	0.12	0.11	0.08	0.04	0.02	-0.02	-0.06

ANC visit, antenatal care visit

Table S11: Posterior mean difference by considering with and without country level predictors for at least four ANC visits

**Country**	Mean difference with and without country level predictors						
	**2000**	**2005**	**2010**	**2015**	**2020**	**2025**	**2030**
Afghanistan	-1.10	-1.00	-0.70	0.40	2.80	6.60	10.70
Albania	1.50	1.00	0.60	0.30	0.20	0.30	0.50
Angola	0.10	0.10	0.10	0.30	0.60	1.00	1.40
Armenia	0.30	0.00	0.00	0.10	0.10	0.10	0.10
Bangladesh	-0.30	-0.20	0.00	0.40	0.90	1.50	2.20
Benin	-0.70	-0.70	-0.60	-0.30	0.20	1.10	2.30
Burkina Faso	0.30	-0.20	-1.10	-2.30	-3.10	-3.60	-3.50
Burundi	1.30	0.90	0.40	-0.20	-0.80	-1.30	-1.60
Cambodia	0.00	-0.20	-0.50	-0.60	-0.40	-0.20	-0.10
Cameroon	0.20	0.00	-0.20	-0.30	-0.30	-0.30	-0.20
Central African Republic	0.20	-0.30	-0.70	-1.00	-1.40	-1.70	-1.80
Chad	0.00	0.00	-0.10	-0.10	-0.10	-0.10	0.00
Comoros	0.40	0.40	0.40	0.40	0.60	0.80	1.20
Congo	-0.80	-0.60	-0.30	0.10	0.50	1.00	1.50
Cote d'Ivoire	1.20	0.90	0.50	0.00	-0.60	-1.00	-1.30
Democratic Republic of the Congo	0.90	0.50	0.10	-0.10	-0.30	-0.30	-0.10
Dominican Republic	1.50	0.50	-0.10	-0.30	-0.40	-0.40	-0.40
Ethiopia	-0.20	-0.20	0.00	0.10	0.50	1.10	1.70
Ghana	-0.10	-0.20	-0.20	-0.20	-0.20	-0.20	-0.10
Guinea	0.10	0.10	0.10	0.00	0.00	0.00	0.10
Haiti	1.70	0.70	-0.20	-0.90	-1.40	-1.70	-1.60
Honduras	1.00	0.40	-0.30	-0.80	-1.30	-1.80	-2.20
India	-0.40	-0.30	-0.20	0.10	0.30	0.50	0.70
Indonesia	-0.60	-0.50	-0.40	-0.10	0.00	0.20	0.30
Kenya	0.00	-0.40	-0.40	0.00	0.50	1.30	2.00
Kyrgyzstan	4.30	2.30	0.90	-0.10	-0.50	-0.60	-0.60
Laos	0.40	0.30	0.10	-0.50	-1.20	-1.70	-1.80
Lesotho	0.10	-0.10	-0.20	-0.20	-0.20	-0.10	0.10
Liberia	1.60	1.00	0.40	-0.30	-0.70	-0.80	-0.80
Madagascar	3.30	1.10	-1.90	-4.80	-7.00	-8.20	-8.60
Malawi	0.30	0.00	-0.10	-0.20	-0.30	-0.20	-0.10
Maldives	-1.00	-0.60	-0.20	0.30	0.80	1.50	2.00
Mali	0.40	0.40	0.30	0.30	0.30	0.50	0.80
Mozambique	-0.80	-0.70	-0.50	-0.30	-0.10	0.30	0.60
Myanmar	0.60	0.40	0.00	-0.40	-0.80	-1.20	-1.50
Nepal	-0.30	-0.30	-0.20	0.20	0.60	1.00	0.90
Niger	1.10	1.00	0.30	-1.00	-2.50	-3.70	-4.10
Nigeria	-0.10	-0.20	-0.20	-0.10	-0.10	0.10	0.40
Pakistan	0.40	0.20	-0.20	-0.60	-1.10	-1.50	-1.90
Papua New Guinea	4.70	3.80	2.40	0.80	-0.70	-2.20	-3.40
Philippines	0.20	0.10	-0.10	-0.20	-0.30	-0.30	-0.20
Rwanda	-0.10	-0.20	-0.20	0.00	0.50	1.10	1.30

Sao Tome and Principe	0.40	0.50	0.50	0.50	0.50	0.60	0.60
Senegal	0.10	0.00	0.10	0.00	0.00	0.20	0.20
Sierra Leone	1.80	1.10	0.40	-0.10	-0.50	-0.70	-0.80
Tajikistan	3.90	2.70	1.20	-0.10	-1.40	-2.10	-2.60
Tanzania	-0.10	-0.10	0.00	0.00	0.20	0.40	0.60
The Gambia	0.20	0.20	0.20	0.30	0.50	0.70	0.80
Timor-Leste	-2.40	-2.20	-1.40	-0.20	0.80	1.20	1.20
Togo	0.80	0.60	0.30	-0.30	-0.80	-1.30	-1.60
Uganda	0.00	0.00	0.00	0.20	0.30	0.50	0.70
Vietnam	-0.30	-0.30	-0.30	-0.20	-0.10	0.00	0.10
Zambia	-0.10	-0.20	-0.30	-0.20	-0.20	0.00	0.10
Zimbabwe	2.20	1.10	0.20	-0.60	-1.20	-1.60	-1.80
All countries (median)	0.20	0.00	-0.10	-0.10	-0.15	-0.05	0.10
All countries (mean)	0.52	0.24	-0.03	-0.24	-0.38	-0.39	-0.32

ANC visit, antenatal care visit

Table S12: Posterior mean difference by altering prior distribution on hyperparameters for single ANC visit

Country	Mean difference with and without country level predictors						
	2000	2005	2010	2015	2020	2025	2030
Afghanistan	0.10	-0.10	-0.40	-0.70	-0.60	-0.30	-0.10
Albania	0.10	0.10	0.00	0.00	0.20	0.50	0.90
Angola	0.30	0.20	0.10	-0.20	-0.40	-0.60	-0.50
Armenia	0.50	0.00	0.00	0.00	0.00	0.00	0.00
Bangladesh	0.70	0.50	0.20	0.10	0.00	-0.10	0.00
Benin	0.90	0.50	0.10	-0.10	0.00	0.40	0.90
Burkina Faso	0.20	0.10	0.00	0.00	0.00	-0.10	0.00
Burundi	0.20	0.10	0.00	0.00	0.00	0.00	0.00
Cambodia	0.10	0.10	0.00	0.10	0.00	0.00	0.00
Cameroon	-0.30	-0.40	-0.40	-0.30	-0.10	0.00	0.20
Central African Republic	0.90	0.50	0.20	0.10	0.00	0.00	0.00
Chad	0.00	0.00	0.00	0.10	0.30	0.40	0.50
Comoros	0.90	0.40	0.10	0.00	-0.20	-0.10	-0.10
Congo	-0.40	-0.20	0.10	0.30	0.50	0.60	0.60
Cote d'Ivoire	-0.60	-0.30	0.00	0.20	0.40	0.50	0.50
Democratic Republic of the Congo	-0.20	-0.20	-0.10	0.00	0.20	0.50	0.80
Dominican Republic	0.40	-0.10	0.00	0.00	0.00	0.00	0.00
Ethiopia	0.10	0.10	0.10	0.10	0.10	0.00	0.10
Ghana	0.10	0.10	0.00	0.00	-0.10	0.00	-0.10
Guinea	0.40	0.20	0.10	0.00	0.00	0.00	0.20
Haiti	0.40	0.10	-0.20	-0.20	-0.10	0.10	0.20
Honduras	0.50	0.10	0.00	0.00	-0.10	0.00	0.00
India	0.20	0.10	0.10	0.10	0.10	0.10	0.20
Indonesia	-0.10	-0.10	-0.10	-0.10	0.00	-0.10	0.00
Kenya	-0.20	-0.40	-0.20	0.00	0.20	0.20	0.30
Kyrgyzstan	-0.50	-0.20	0.00	0.00	0.00	0.00	0.00
Laos	0.00	0.00	0.10	0.20	0.30	0.20	0.20
Lesotho	-0.10	-0.20	-0.10	-0.20	-0.10	0.00	-0.10
Liberia	1.10	0.30	0.00	-0.10	0.00	-0.10	0.00
Madagascar	0.30	-0.10	-0.40	-0.40	-0.30	-0.30	-0.10
Malawi	0.10	0.00	0.00	0.00	0.00	0.00	0.00
Maldives	0.00	0.00	0.00	0.00	0.10	0.10	0.30
Mali	0.30	0.10	-0.10	-0.10	-0.10	0.10	0.20
Mozambique	0.40	0.20	0.10	-0.10	-0.10	-0.20	-0.30
Myanmar	0.60	0.50	0.40	0.20	-0.10	-0.10	-0.30
Nepal	-0.40	-0.40	-0.20	0.00	0.10	0.30	0.20
Niger	-0.10	-0.30	-0.30	-0.20	-0.10	0.00	0.00
Nigeria	0.80	0.50	0.20	0.10	-0.10	-0.20	-0.20
Pakistan	-0.90	-0.80	-0.60	-0.30	0.00	0.20	0.30

Papua New Guinea	-1.00	-0.60	-0.20	0.30	0.80	1.20	1.40
Philippines	-0.70	-0.50	-0.30	-0.10	0.10	0.10	0.30
Rwanda	0.10	0.00	0.00	0.00	0.00	0.00	0.00
Sao Tome and Principe	0.50	0.10	0.00	0.00	0.00	0.00	0.00
Senegal	0.20	0.10	0.00	0.00	0.00	0.00	0.00
Sierra Leone	0.60	0.10	0.00	0.00	0.00	-0.10	0.00
Tajikistan	0.20	0.10	0.20	0.20	0.20	0.30	0.20
Tanzania	0.40	0.20	0.10	0.10	0.00	0.00	-0.10
The Gambia	0.00	0.00	0.00	0.10	0.00	0.00	0.00
Timor-Leste	1.80	1.20	0.70	0.40	0.10	0.00	-0.10
Togo	0.20	0.30	0.30	0.40	0.60	0.80	1.00
Uganda	0.00	0.00	0.00	0.00	0.00	0.00	0.00
Vietnam	-0.20	-0.20	-0.10	0.00	0.10	0.10	0.20
Zambia	0.20	0.10	0.00	0.00	0.00	0.00	0.10
Zimbabwe	0.00	-0.10	-0.10	-0.10	0.00	0.20	0.30
All countries (median)	0.15	0.10	0.00	0.00	0.00	0.00	0.00
All countries (mean)	0.17	0.04	0.00	0.01	0.05	0.09	0.15

ANC visit, antenatal care visit

Table S13: Posterior mean difference by altering prior distribution on hyperparameters for at least four ANC visits

Country	Mean difference with and without country level predictors						
	**2000**	**2005**	**2010**	**2015**	**2020**	**2025**	**2030**
Afghanistan	0.20	0.20	0.10	-0.20	-1.00	-2.10	-3.10
Albania	-0.10	-0.20	-0.20	-0.30	-0.40	-0.60	-0.70
Angola	-0.10	0.00	-0.10	-0.10	-0.30	-0.40	-0.50
Armenia	-0.20	-0.20	-0.10	-0.10	-0.10	0.00	0.00
Bangladesh	-0.20	-0.30	-0.30	-0.30	-0.40	-0.50	-0.50
Benin	-0.50	-0.30	-0.20	-0.20	-0.10	-0.20	-0.40
Burkina Faso	-0.10	0.00	0.10	0.30	0.40	0.40	0.50
Burundi	0.60	0.50	0.30	0.10	-0.10	-0.40	-0.70
Cambodia	0.00	0.00	-0.10	0.00	0.00	0.00	0.00
Cameroon	-0.20	0.00	0.20	0.40	0.40	0.50	0.60
Central African Republic	0.00	-0.10	-0.20	-0.30	-0.30	-0.40	-0.60
Chad	-0.20	-0.20	-0.10	0.00	0.20	0.40	0.70
Comoros	-1.20	-0.80	-0.40	0.20	0.60	1.10	1.30
Congo	0.10	0.10	0.10	0.10	0.10	0.00	0.00
Cote d'Ivoire	0.60	0.50	0.30	0.00	-0.30	-0.60	-0.70
Democratic Republic of the Congo	-0.20	-0.10	0.10	0.20	0.30	0.30	0.40
Dominican Republic	0.20	0.10	-0.10	0.00	-0.10	-0.10	-0.10
Ethiopia	0.10	0.00	0.00	0.00	-0.20	-0.40	-0.60
Ghana	0.20	0.20	0.10	0.10	0.10	0.00	0.00
Guinea	0.10	0.10	0.10	0.10	0.10	0.10	0.10
Haiti	-0.20	-0.10	0.10	0.20	0.30	0.30	0.20
Honduras	0.00	-0.10	-0.10	-0.10	-0.20	-0.30	-0.40
India	0.20	0.20	0.20	0.30	0.20	0.20	0.20
Indonesia	0.00	0.10	0.10	0.10	0.20	0.20	0.10
Kenya	-0.10	0.00	0.00	0.10	0.10	0.00	0.00
Kyrgyzstan	-1.70	-1.00	-0.40	0.00	0.20	0.30	0.30
Laos	-0.30	-0.30	-0.30	-0.20	-0.20	0.10	0.30
Lesotho	0.20	0.20	0.10	0.00	0.00	-0.10	-0.30
Liberia	-0.70	-0.50	-0.20	0.00	0.00	0.00	0.00
Madagascar	-0.20	0.10	0.40	0.80	1.10	1.20	1.20
Malawi	0.10	-0.10	0.00	-0.20	-0.20	-0.30	-0.30
Maldives	-0.10	-0.10	-0.10	-0.20	-0.20	-0.40	-0.40
Mali	-0.20	-0.30	-0.20	-0.20	-0.20	-0.30	-0.40
Mozambique	-0.30	-0.10	0.20	0.40	0.60	0.80	0.90
Myanmar	-1.00	-0.80	-0.50	-0.10	0.40	0.90	1.40
Nepal	-0.30	-0.30	-0.30	-0.20	-0.10	0.00	0.10
Niger	-0.10	-0.10	0.00	0.20	0.50	0.60	0.70
Nigeria	-0.10	-0.10	-0.10	-0.10	0.00	0.00	-0.10
Pakistan	0.20	0.20	0.20	0.20	0.10	0.00	-0.20

Papua New Guinea	0.00	-0.10	-0.20	-0.20	-0.20	-0.30	-0.40
Philippines	0.40	0.20	0.00	-0.10	-0.10	-0.20	-0.30
Rwanda	-0.10	0.00	0.00	0.00	0.00	-0.10	-0.10
Sao Tome and Principe	0.20	0.20	0.10	0.00	0.00	0.00	0.00
Senegal	0.30	0.20	0.20	0.00	0.00	-0.20	-0.30
Sierra Leone	-0.10	0.00	0.00	0.00	0.00	0.10	0.00
Tajikistan	-0.10	-0.10	-0.20	-0.30	-0.40	-0.40	-0.40
Tanzania	-0.60	-0.50	-0.50	-0.40	-0.40	-0.30	-0.30
The Gambia	0.10	0.00	-0.10	-0.20	-0.30	-0.40	-0.50
Timor-Leste	-0.10	0.00	0.00	0.00	0.10	0.00	-0.10
Togo	0.20	0.10	0.00	-0.20	-0.50	-0.80	-1.10
Uganda	-0.30	-0.10	0.10	0.20	0.30	0.50	0.50
Vietnam	-0.20	-0.10	0.00	0.20	0.30	0.50	0.60
Zambia	-0.20	-0.10	0.10	0.30	0.40	0.50	0.60
Zimbabwe	-0.40	-0.30	-0.20	-0.10	-0.10	-0.10	-0.20
All countries (median)	-0.10	-0.10	0.00	0.00	0.00	0.00	-0.05
All countries (mean)	-0.12	-0.08	-0.04	0.01	0.03	0.03	0.01

ANC visit, antenatal care visit

Table S14: National level estimate of Gelman Rubin Potential scale reduction factors (PSRF) for single ANC visit

**Country**	Potential scale reduction factors (Point estimate; Upper C.I)						
	**2000**	**2005**	**2010**	**2015**	**2020**	**2025**	**2030**
Afghanistan	(1.008;1.028)	(1.008;1.028)	(1.008;1.028)	(1.008;1.028)	(1.008;1.028)	(1.008;1.028)	(1.008;1.028)
Albania	(1.008;1.028)	(1.008;1.028)	(1.008;1.028)	(1.008;1.028)	(1.008;1.028)	(1.008;1.028)	(1.008;1.028)
Angola	(1.008;1.028)	(1.008;1.028)	(1.008;1.028)	(1.008;1.028)	(1.008;1.028)	(1.008;1.028)	(1.008;1.028)
Armenia	(1.008;1.028)	(1.008;1.028)	(1.008;1.028)	(1.008;1.028)	(1.008;1.028)	(1.008;1.028)	(1.008;1.028)
Bangladesh	(1.008;1.028)	(1.008;1.028)	(1.008;1.028)	(1.008;1.028)	(1.008;1.028)	(1.008;1.028)	(1.008;1.028)
Benin	(1.008;1.028)	(1.008;1.028)	(1.008;1.028)	(1.008;1.028)	(1.008;1.028)	(1.008;1.028)	(1.008;1.028)
Burkina Faso	(1.008;1.028)	(1.008;1.028)	(1.008;1.028)	(1.008;1.028)	(1.008;1.028)	(1.008;1.028)	(1.008;1.028)
Burundi	(1.008;1.028)	(1.008;1.028)	(1.008;1.028)	(1.008;1.028)	(1.008;1.028)	(1.008;1.028)	(1.008;1.028)
Cambodia	(1.008;1.028)	(1.008;1.028)	(1.008;1.028)	(1.008;1.028)	(1.008;1.028)	(1.008;1.028)	(1.008;1.028)
Cameroon	(1.008;1.028)	(1.008;1.028)	(1.008;1.028)	(1.008;1.028)	(1.008;1.028)	(1.008;1.028)	(1.008;1.028)
Central African Republic	(1.008;1.028)	(1.008;1.028)	(1.008;1.028)	(1.008;1.028)	(1.008;1.028)	(1.008;1.028)	(1.008;1.028)
Chad	(1.008;1.028)	(1.008;1.028)	(1.008;1.028)	(1.008;1.028)	(1.008;1.028)	(1.008;1.028)	(1.008;1.028)
Comoros	(1.008;1.028)	(1.008;1.028)	(1.008;1.028)	(1.008;1.028)	(1.008;1.028)	(1.008;1.028)	(1.008;1.028)
Congo	(1.008;1.028)	(1.008;1.028)	(1.008;1.028)	(1.008;1.028)	(1.008;1.028)	(1.008;1.028)	(1.008;1.028)
Cote d'Ivoire	(1.008;1.028)	(1.008;1.028)	(1.008;1.028)	(1.008;1.028)	(1.008;1.028)	(1.008;1.028)	(1.008;1.028)
Democratic Republic of the Congo	(1.008;1.028)	(1.008;1.028)	(1.008;1.028)	(1.008;1.028)	(1.008;1.028)	(1.008;1.028)	(1.008;1.028)
Dominican Republic	(1.008;1.028)	(1.008;1.028)	(1.008;1.028)	(1.008;1.028)	(1.008;1.028)	(1.008;1.028)	(1.008;1.028)
Ethiopia	(1.008;1.028)	(1.008;1.028)	(1.008;1.028)	(1.008;1.028)	(1.008;1.028)	(1.008;1.028)	(1.008;1.028)
Ghana	(1.008;1.028)	(1.008;1.028)	(1.008;1.028)	(1.008;1.028)	(1.008;1.028)	(1.008;1.028)	(1.008;1.028)
Guinea	(1.008;1.028)	(1.008;1.028)	(1.008;1.028)	(1.008;1.028)	(1.008;1.028)	(1.008;1.028)	(1.008;1.028)
Haiti	(1.008;1.028)	(1.008;1.028)	(1.008;1.028)	(1.008;1.028)	(1.008;1.028)	(1.008;1.028)	(1.008;1.028)
Honduras	(1.008;1.028)	(1.008;1.028)	(1.008;1.028)	(1.008;1.028)	(1.008;1.028)	(1.008;1.028)	(1.008;1.028)
India	(1.008;1.028)	(1.008;1.028)	(1.008;1.028)	(1.008;1.028)	(1.008;1.028)	(1.008;1.028)	(1.008;1.028)
Indonesia	(1.008;1.028)	(1.008;1.028)	(1.008;1.028)	(1.008;1.028)	(1.008;1.028)	(1.008;1.028)	(1.008;1.028)
Kenya	(1.008;1.028)	(1.008;1.028)	(1.008;1.028)	(1.008;1.028)	(1.008;1.028)	(1.008;1.028)	(1.008;1.028)
Kyrgyzstan	(1.008;1.028)	(1.008;1.028)	(1.008;1.028)	(1.008;1.028)	(1.008;1.028)	(1.008;1.028)	(1.008;1.028)
Laos	(1.008;1.028)	(1.008;1.028)	(1.008;1.028)	(1.008;1.028)	(1.008;1.028)	(1.008;1.028)	(1.008;1.028)
Lesotho	(1.008;1.028)	(1.008;1.028)	(1.008;1.028)	(1.008;1.028)	(1.008;1.028)	(1.008;1.028)	(1.008;1.028)

Liberia	(1.008;1.028)	(1.008;1.028)	(1.008;1.028)	(1.008;1.028)	(1.008;1.028)	(1.008;1.028)	(1.008;1.028)
Madagascar	(1.008;1.028)	(1.008;1.028)	(1.008;1.028)	(1.008;1.028)	(1.008;1.028)	(1.008;1.028)	(1.008;1.028)
Malawi	(1.008;1.028)	(1.008;1.028)	(1.008;1.028)	(1.008;1.028)	(1.008;1.028)	(1.008;1.028)	(1.008;1.028)
Maldives	(1.008;1.028)	(1.008;1.028)	(1.008;1.028)	(1.008;1.028)	(1.008;1.028)	(1.008;1.028)	(1.008;1.028)
Mali	(1.008;1.028)	(1.008;1.028)	(1.008;1.028)	(1.008;1.028)	(1.008;1.028)	(1.008;1.028)	(1.008;1.028)
Mozambique	(1.008;1.028)	(1.008;1.028)	(1.008;1.028)	(1.008;1.028)	(1.008;1.028)	(1.008;1.028)	(1.008;1.028)
Myanmar	(1.008;1.028)	(1.008;1.028)	(1.008;1.028)	(1.008;1.028)	(1.008;1.028)	(1.008;1.028)	(1.008;1.028)
Nepal	(1.008;1.028)	(1.008;1.028)	(1.008;1.028)	(1.008;1.028)	(1.008;1.028)	(1.008;1.028)	(1.008;1.028)
Niger	(1.008;1.028)	(1.008;1.028)	(1.008;1.028)	(1.008;1.028)	(1.008;1.028)	(1.008;1.028)	(1.008;1.028)
Nigeria	(1.008;1.028)	(1.008;1.028)	(1.008;1.028)	(1.008;1.028)	(1.008;1.028)	(1.008;1.028)	(1.008;1.028)
Pakistan	(1.008;1.028)	(1.008;1.028)	(1.008;1.028)	(1.008;1.028)	(1.008;1.028)	(1.008;1.028)	(1.008;1.028)
Papua New Guinea	(1.008;1.028)	(1.008;1.028)	(1.008;1.028)	(1.008;1.028)	(1.008;1.028)	(1.008;1.028)	(1.008;1.028)
Philippines	(1.008;1.028)	(1.008;1.028)	(1.008;1.028)	(1.008;1.028)	(1.008;1.028)	(1.008;1.028)	(1.008;1.028)
Rwanda	(1.008;1.028)	(1.008;1.028)	(1.008;1.028)	(1.008;1.028)	(1.008;1.028)	(1.008;1.028)	(1.008;1.028)
Sao Tome and Principe	(1.008;1.028)	(1.008;1.028)	(1.008;1.028)	(1.008;1.028)	(1.008;1.028)	(1.008;1.028)	(1.008;1.028)
Senegal	(1.008;1.028)	(1.008;1.028)	(1.008;1.028)	(1.008;1.028)	(1.008;1.028)	(1.008;1.028)	(1.008;1.028)
Sierra Leone	(1.008;1.028)	(1.008;1.028)	(1.008;1.028)	(1.008;1.028)	(1.008;1.028)	(1.008;1.028)	(1.008;1.028)
Tajikistan	(1.008;1.028)	(1.008;1.028)	(1.008;1.028)	(1.008;1.028)	(1.008;1.028)	(1.008;1.028)	(1.008;1.028)
Tanzania	(1.008;1.028)	(1.008;1.028)	(1.008;1.028)	(1.008;1.028)	(1.008;1.028)	(1.008;1.028)	(1.008;1.028)
The Gambia	(1.008;1.028)	(1.008;1.028)	(1.008;1.028)	(1.008;1.028)	(1.008;1.028)	(1.008;1.028)	(1.008;1.028)
Timor-Leste	(1.008;1.028)	(1.008;1.028)	(1.008;1.028)	(1.008;1.028)	(1.008;1.028)	(1.008;1.028)	(1.008;1.028)
Togo	(1.008;1.028)	(1.008;1.028)	(1.008;1.028)	(1.008;1.028)	(1.008;1.028)	(1.008;1.028)	(1.008;1.028)
Uganda	(1.008;1.028)	(1.008;1.028)	(1.008;1.028)	(1.008;1.028)	(1.008;1.028)	(1.008;1.028)	(1.008;1.028)
Vietnam	(1.008;1.028)	(1.008;1.028)	(1.008;1.028)	(1.008;1.028)	(1.008;1.028)	(1.008;1.028)	(1.008;1.028)
Zambia	(1.008;1.028)	(1.008;1.028)	(1.008;1.028)	(1.008;1.028)	(1.008;1.028)	(1.008;1.028)	(1.008;1.028)
Zimbabwe	(1.008;1.028)	(1.008;1.028)	(1.008;1.028)	(1.008;1.028)	(1.008;1.028)	(1.008;1.028)	(1.008;1.028)

ANC visit, antenatal care visit; C.I, confidence interval

Table S15: National level estimate of Gelman Rubin Potential scale reduction factors (PSRF) for at least four ANC visits

**Country**	Potential scale reduction factors (Point estimate; Upper C.I)						
	**2000**	**2005**	**2010**	**2015**	**2020**	**2025**	**2030**
Afghanistan	(1.01;1.02)	(1.01;1.02)	(1.01;1.02)	(1.01;1.02)	(1.01;1.02)	(1.01;1.02)	(1.01;1.02)
Albania	(1.01;1.02)	(1.01;1.02)	(1.01;1.02)	(1.01;1.02)	(1.01;1.02)	(1.01;1.02)	(1.01;1.02)
Angola	(1.01;1.02)	(1.01;1.02)	(1.01;1.02)	(1.01;1.02)	(1.01;1.02)	(1.01;1.02)	(1.01;1.02)
Armenia	(1.01;1.02)	(1.01;1.02)	(1.01;1.02)	(1.01;1.02)	(1.01;1.02)	(1.01;1.02)	(1.01;1.02)
Bangladesh	(1.01;1.02)	(1.01;1.02)	(1.01;1.02)	(1.01;1.02)	(1.01;1.02)	(1.01;1.02)	(1.01;1.02)
Benin	(1.01;1.02)	(1.01;1.02)	(1.01;1.02)	(1.01;1.02)	(1.01;1.02)	(1.01;1.02)	(1.01;1.02)
Burkina Faso	(1.01;1.02)	(1.01;1.02)	(1.01;1.02)	(1.01;1.02)	(1.01;1.02)	(1.01;1.02)	(1.01;1.02)
Burundi	(1.01;1.02)	(1.01;1.02)	(1.01;1.02)	(1.01;1.02)	(1.01;1.02)	(1.01;1.02)	(1.01;1.02)
Cambodia	(1.01;1.02)	(1.01;1.02)	(1.01;1.02)	(1.01;1.02)	(1.01;1.02)	(1.01;1.02)	(1.01;1.02)
Cameroon	(1.01;1.02)	(1.01;1.02)	(1.01;1.02)	(1.01;1.02)	(1.01;1.02)	(1.01;1.02)	(1.01;1.02)
Central African Republic	(1.01;1.02)	(1.01;1.02)	(1.01;1.02)	(1.01;1.02)	(1.01;1.02)	(1.01;1.02)	(1.01;1.02)
Chad	(1.01;1.02)	(1.01;1.02)	(1.01;1.02)	(1.01;1.02)	(1.01;1.02)	(1.01;1.02)	(1.01;1.02)
Comoros	(1.01;1.02)	(1.01;1.02)	(1.01;1.02)	(1.01;1.02)	(1.01;1.02)	(1.01;1.02)	(1.01;1.02)
Congo	(1.01;1.02)	(1.01;1.02)	(1.01;1.02)	(1.01;1.02)	(1.01;1.02)	(1.01;1.02)	(1.01;1.02)
Cote d'Ivoire	(1.01;1.02)	(1.01;1.02)	(1.01;1.02)	(1.01;1.02)	(1.01;1.02)	(1.01;1.02)	(1.01;1.02)
Democratic Republic of the Congo	(1.01;1.02)	(1.01;1.02)	(1.01;1.02)	(1.01;1.02)	(1.01;1.02)	(1.01;1.02)	(1.01;1.02)
Dominican Republic	(1.01;1.02)	(1.01;1.02)	(1.01;1.02)	(1.01;1.02)	(1.01;1.02)	(1.01;1.02)	(1.01;1.02)
Ethiopia	(1.01;1.02)	(1.01;1.02)	(1.01;1.02)	(1.01;1.02)	(1.01;1.02)	(1.01;1.02)	(1.01;1.02)
Ghana	(1.01;1.02)	(1.01;1.02)	(1.01;1.02)	(1.01;1.02)	(1.01;1.02)	(1.01;1.02)	(1.01;1.02)
Guinea	(1.01;1.02)	(1.01;1.02)	(1.01;1.02)	(1.01;1.02)	(1.01;1.02)	(1.01;1.02)	(1.01;1.02)
Haiti	(1.01;1.02)	(1.01;1.02)	(1.01;1.02)	(1.01;1.02)	(1.01;1.02)	(1.01;1.02)	(1.01;1.02)
Honduras	(1.01;1.02)	(1.01;1.02)	(1.01;1.02)	(1.01;1.02)	(1.01;1.02)	(1.01;1.02)	(1.01;1.02)
India	(1.01;1.02)	(1.01;1.02)	(1.01;1.02)	(1.01;1.02)	(1.01;1.02)	(1.01;1.02)	(1.01;1.02)
Indonesia	(1.01;1.02)	(1.01;1.02)	(1.01;1.02)	(1.01;1.02)	(1.01;1.02)	(1.01;1.02)	(1.01;1.02)
Kenya	(1.01;1.02)	(1.01;1.02)	(1.01;1.02)	(1.01;1.02)	(1.01;1.02)	(1.01;1.02)	(1.01;1.02)
Kyrgyzstan	(1.01;1.02)	(1.01;1.02)	(1.01;1.02)	(1.01;1.02)	(1.01;1.02)	(1.01;1.02)	(1.01;1.02)
Laos	(1.01;1.02)	(1.01;1.02)	(1.01;1.02)	(1.01;1.02)	(1.01;1.02)	(1.01;1.02)	(1.01;1.02)
Lesotho	(1.01;1.02)	(1.01;1.02)	(1.01;1.02)	(1.01;1.02)	(1.01;1.02)	(1.01;1.02)	(1.01;1.02)

Liberia	(1.01;1.02)	(1.01;1.02)	(1.01;1.02)	(1.01;1.02)	(1.01;1.02)	(1.01;1.02)	(1.01;1.02)
Madagascar	(1.01;1.02)	(1.01;1.02)	(1.01;1.02)	(1.01;1.02)	(1.01;1.02)	(1.01;1.02)	(1.01;1.02)
Malawi	(1.01;1.02)	(1.01;1.02)	(1.01;1.02)	(1.01;1.02)	(1.01;1.02)	(1.01;1.02)	(1.01;1.02)
Maldives	(1.01;1.02)	(1.01;1.02)	(1.01;1.02)	(1.01;1.02)	(1.01;1.02)	(1.01;1.02)	(1.01;1.02)
Mali	(1.01;1.02)	(1.01;1.02)	(1.01;1.02)	(1.01;1.02)	(1.01;1.02)	(1.01;1.02)	(1.01;1.02)
Mozambique	(1.01;1.02)	(1.01;1.02)	(1.01;1.02)	(1.01;1.02)	(1.01;1.02)	(1.01;1.02)	(1.01;1.02)
Myanmar	(1.01;1.02)	(1.01;1.02)	(1.01;1.02)	(1.01;1.02)	(1.01;1.02)	(1.01;1.02)	(1.01;1.02)
Nepal	(1.01;1.02)	(1.01;1.02)	(1.01;1.02)	(1.01;1.02)	(1.01;1.02)	(1.01;1.02)	(1.01;1.02)
Niger	(1.01;1.02)	(1.01;1.02)	(1.01;1.02)	(1.01;1.02)	(1.01;1.02)	(1.01;1.02)	(1.01;1.02)
Nigeria	(1.01;1.02)	(1.01;1.02)	(1.01;1.02)	(1.01;1.02)	(1.01;1.02)	(1.01;1.02)	(1.01;1.02)
Pakistan	(1.01;1.02)	(1.01;1.02)	(1.01;1.02)	(1.01;1.02)	(1.01;1.02)	(1.01;1.02)	(1.01;1.02)
Papua New Guinea	(1.01;1.02)	(1.01;1.02)	(1.01;1.02)	(1.01;1.02)	(1.01;1.02)	(1.01;1.02)	(1.01;1.02)
Philippines	(1.01;1.02)	(1.01;1.02)	(1.01;1.02)	(1.01;1.02)	(1.01;1.02)	(1.01;1.02)	(1.01;1.02)
Rwanda	(1.01;1.02)	(1.01;1.02)	(1.01;1.02)	(1.01;1.02)	(1.01;1.02)	(1.01;1.02)	(1.01;1.02)
Sao Tome and Principe	(1.01;1.02)	(1.01;1.02)	(1.01;1.02)	(1.01;1.02)	(1.01;1.02)	(1.01;1.02)	(1.01;1.02)
Senegal	(1.01;1.02)	(1.01;1.02)	(1.01;1.02)	(1.01;1.02)	(1.01;1.02)	(1.01;1.02)	(1.01;1.02)
Sierra Leone	(1.01;1.02)	(1.01;1.02)	(1.01;1.02)	(1.01;1.02)	(1.01;1.02)	(1.01;1.02)	(1.01;1.02)
Tajikistan	(1.01;1.02)	(1.01;1.02)	(1.01;1.02)	(1.01;1.02)	(1.01;1.02)	(1.01;1.02)	(1.01;1.02)
Tanzania	(1.01;1.02)	(1.01;1.02)	(1.01;1.02)	(1.01;1.02)	(1.01;1.02)	(1.01;1.02)	(1.01;1.02)
The Gambia	(1.01;1.02)	(1.01;1.02)	(1.01;1.02)	(1.01;1.02)	(1.01;1.02)	(1.01;1.02)	(1.01;1.02)
Timor-Leste	(1.01;1.02)	(1.01;1.02)	(1.01;1.02)	(1.01;1.02)	(1.01;1.02)	(1.01;1.02)	(1.01;1.02)
Togo	(1.01;1.02)	(1.01;1.02)	(1.01;1.02)	(1.01;1.02)	(1.01;1.02)	(1.01;1.02)	(1.01;1.02)
Uganda	(1.01;1.02)	(1.01;1.02)	(1.01;1.02)	(1.01;1.02)	(1.01;1.02)	(1.01;1.02)	(1.01;1.02)
Vietnam	(1.01;1.02)	(1.01;1.02)	(1.01;1.02)	(1.01;1.02)	(1.01;1.02)	(1.01;1.02)	(1.01;1.02)
Zambia	(1.01;1.02)	(1.01;1.02)	(1.01;1.02)	(1.01;1.02)	(1.01;1.02)	(1.01;1.02)	(1.01;1.02)
Zimbabwe	(1.01;1.02)	(1.01;1.02)	(1.01;1.02)	(1.01;1.02)	(1.01;1.02)	(1.01;1.02)	(1.01;1.02)

ANC visit, antenatal care visit; C.I, confidence interval

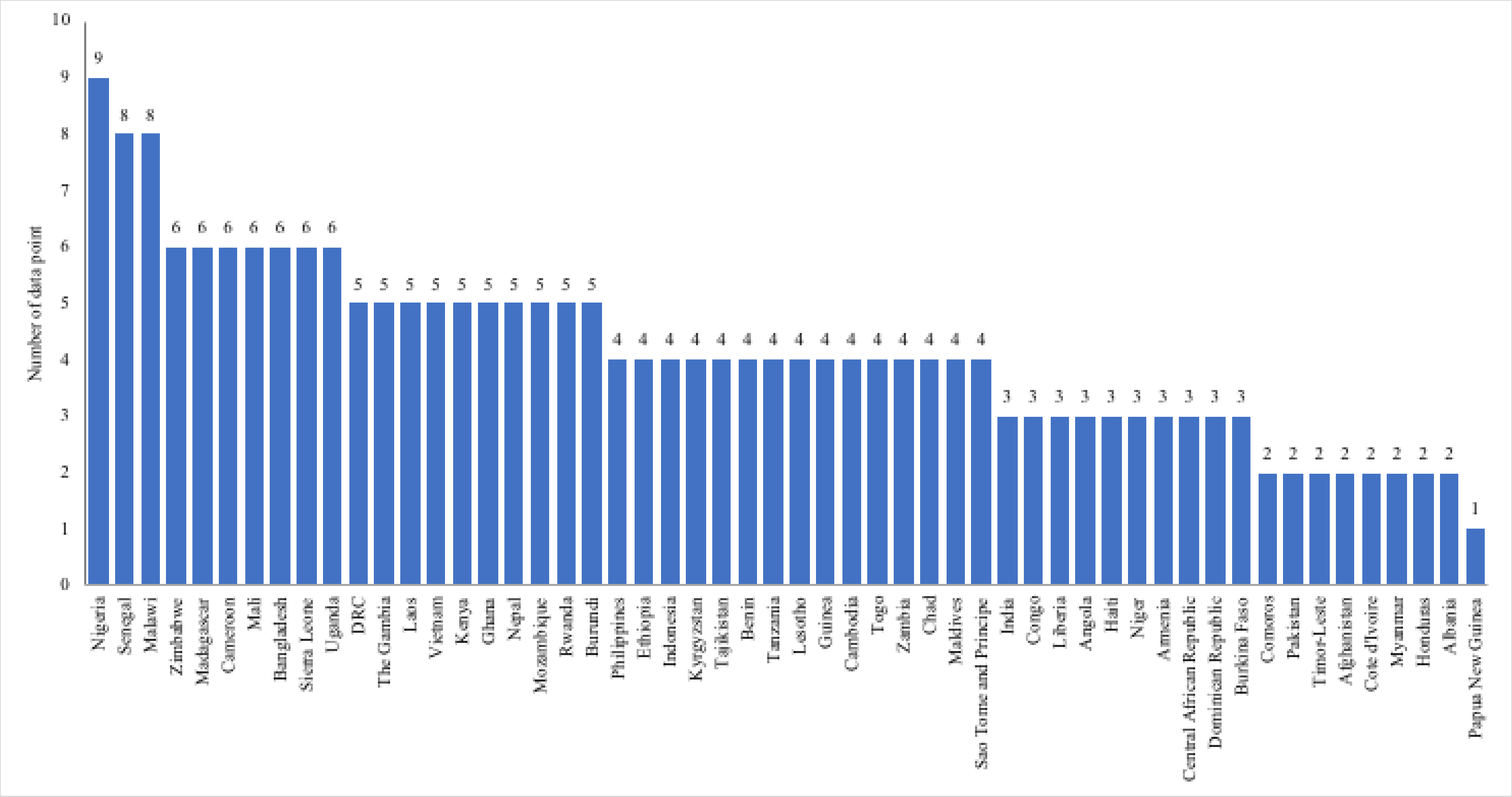

Figure S1: Number of data points by country

Figure S2: Detail years-specific observed and predicted coverage of single ANC visit by area of residence (national, urban, and rural)

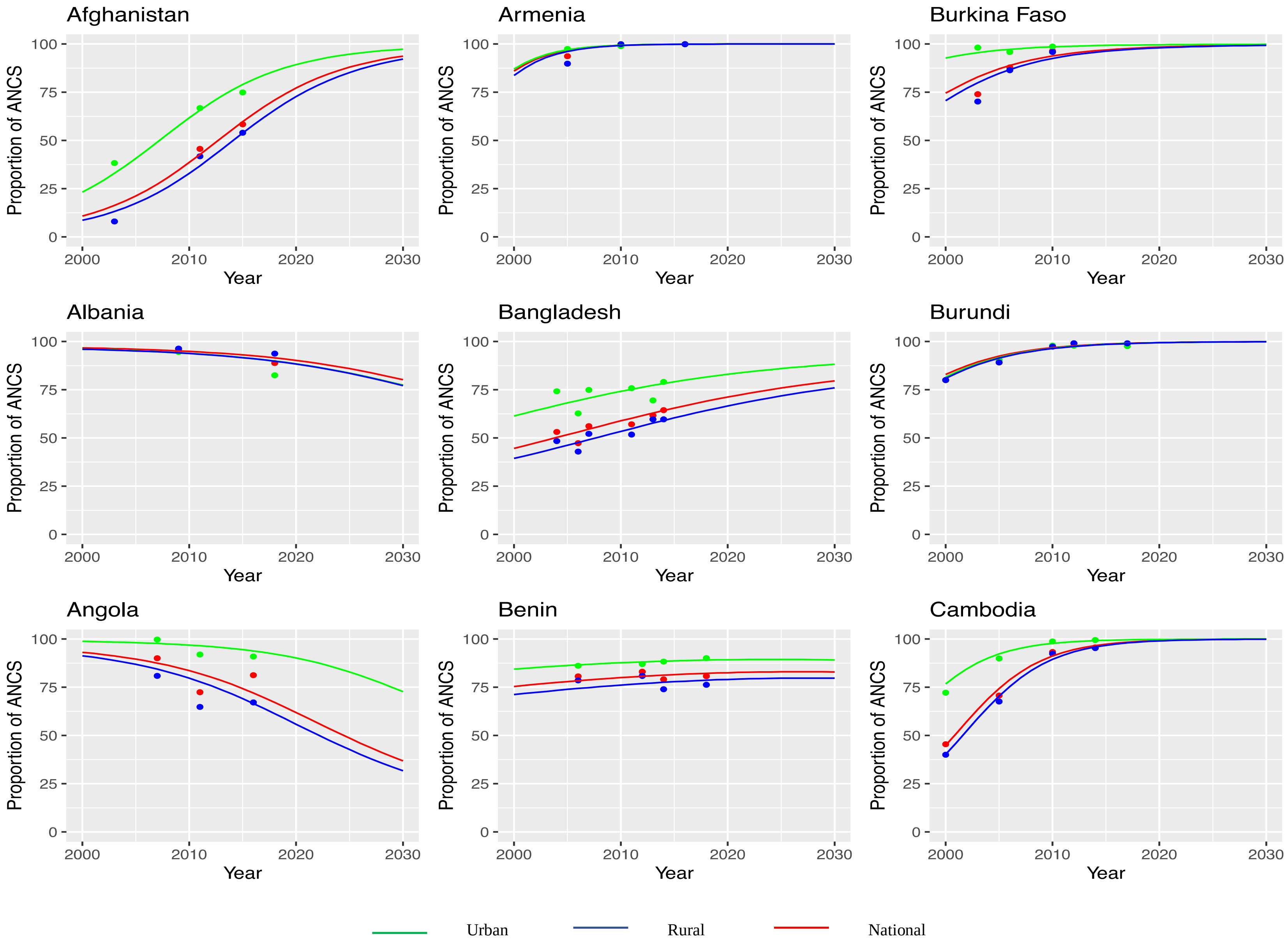

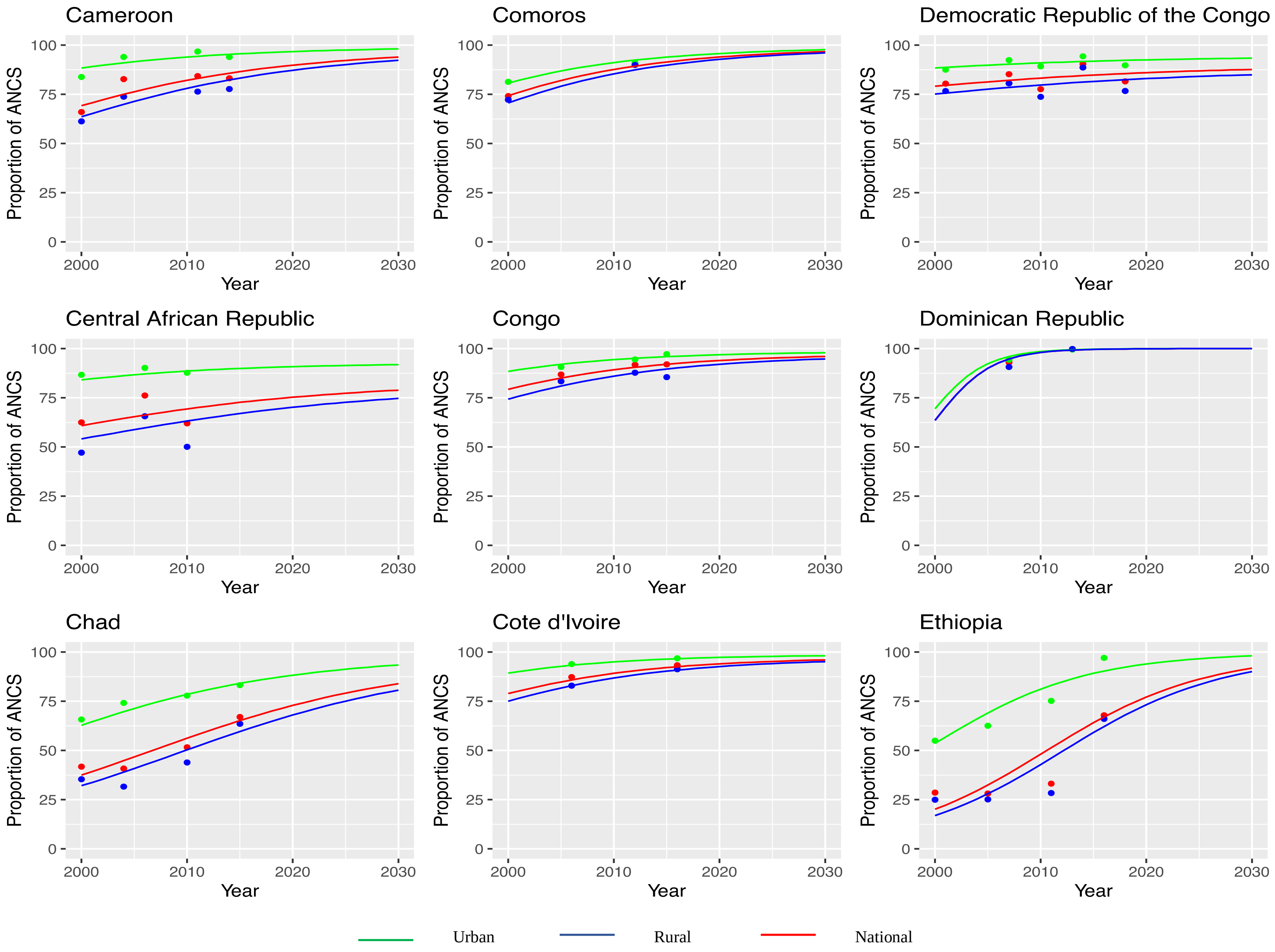
Cameroon
Comoros
Democratic Republic of the Congo
Central African Republic
Congo
Dominican Republic
Chad
Cote d'Ivoire
Ethiopia
Proportion of ANCS
Year
0
25
50
75
100
2000
2010
2020
2030
Urban
Rural
National

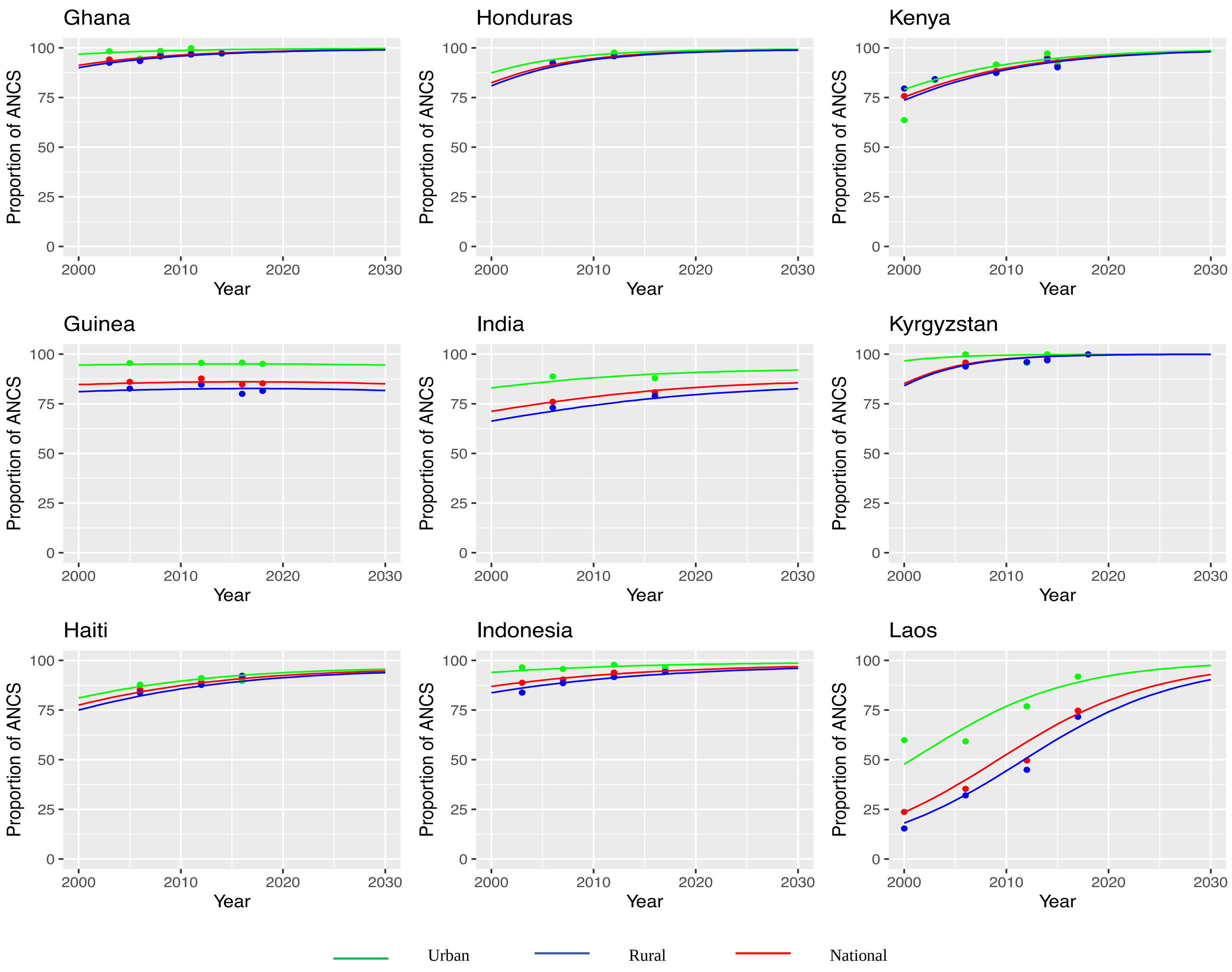
Ghana
Honduras
Kenya
Guinea
India
Kyrgyzstan
Haiti
Indonesia
Laos
Proportion of ANCS
Year
0
25
50
75
100
2000
2010
2020
2030
Urban
Rural
National

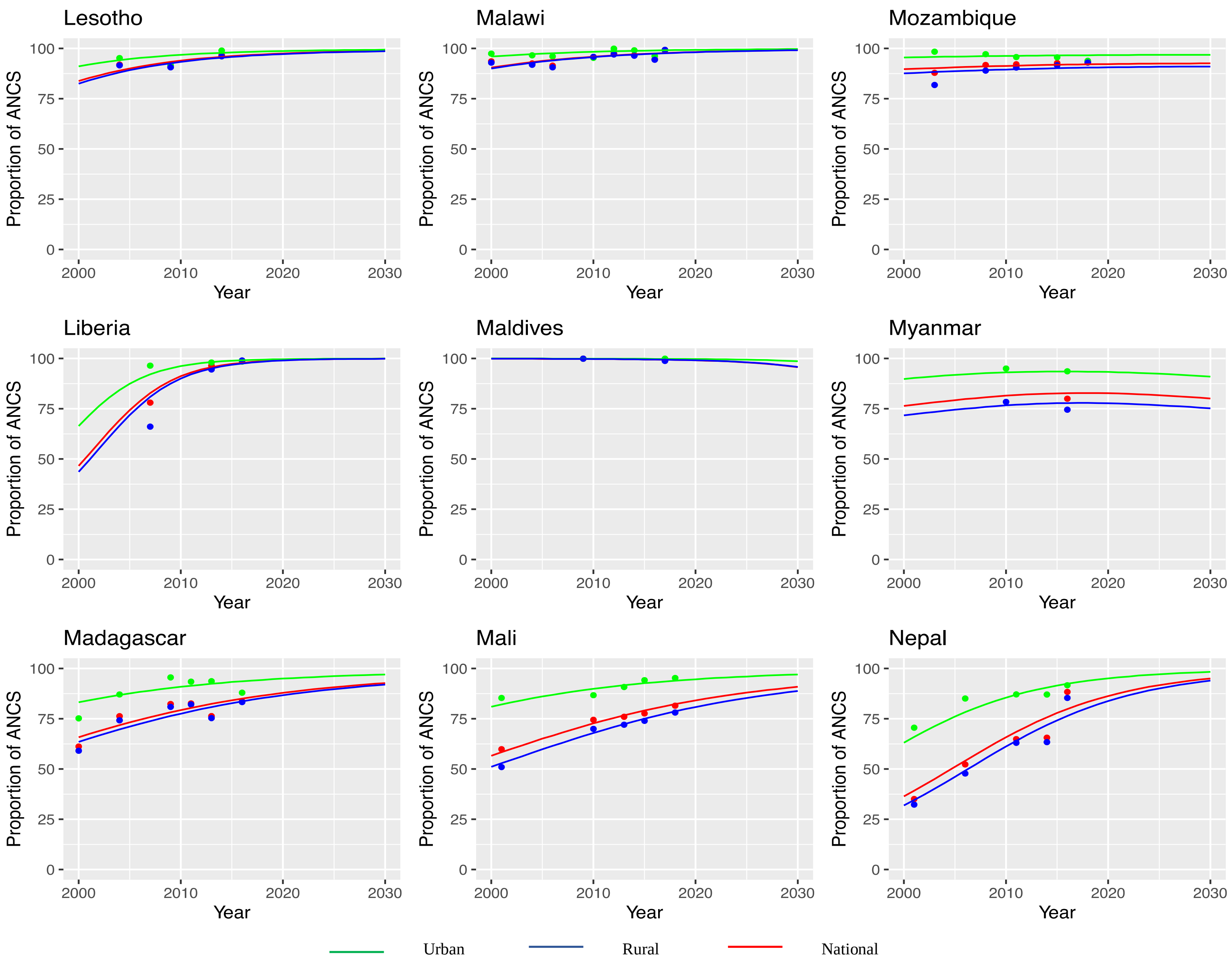

Lesotho
Malawi
Mozambique
Liberia
Maldives
Myanmar
Madagascar
Mali
Nepal
Proportion of ANCS
Year
Urban
Rural
National

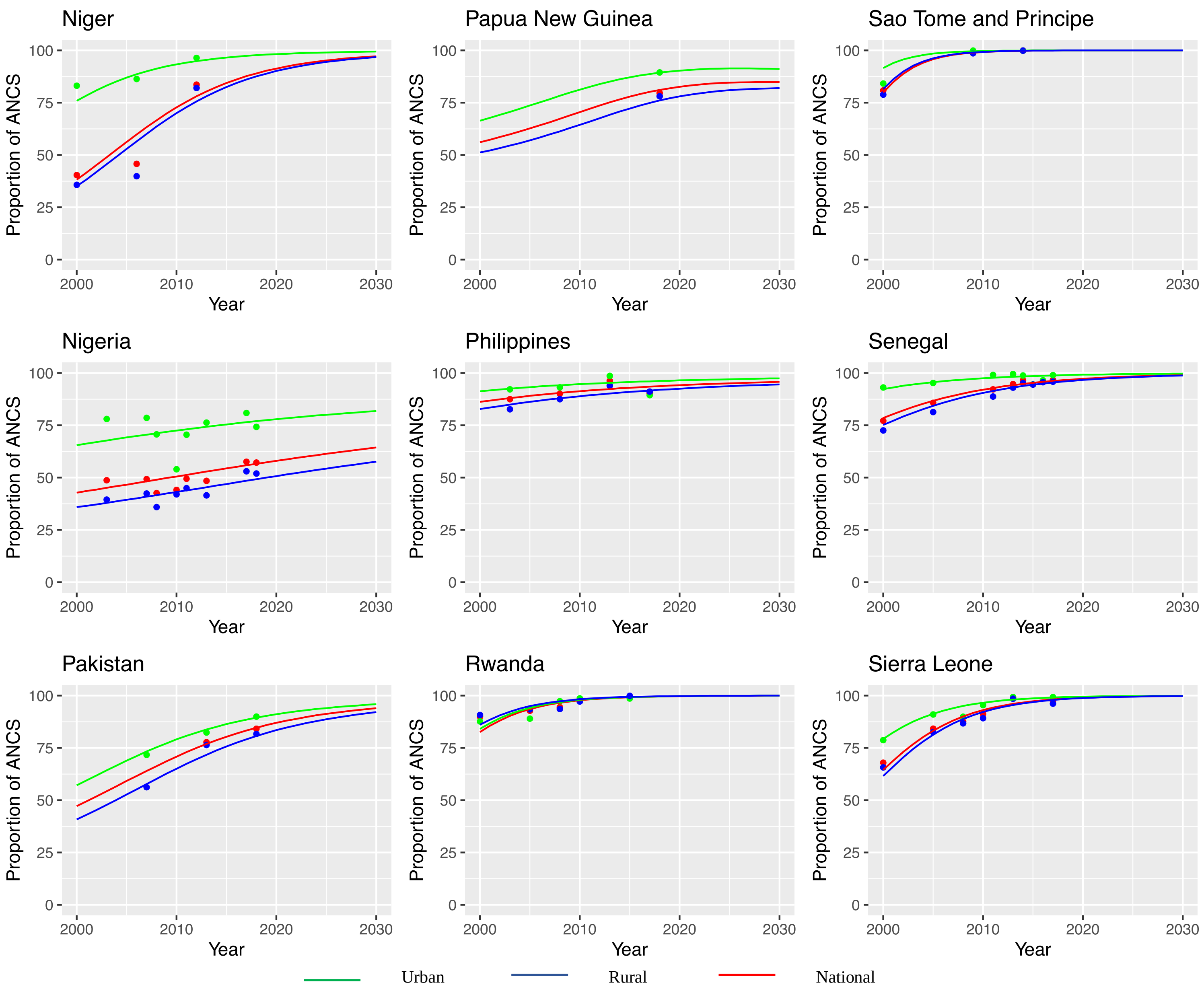
Niger
Papua New Guinea
Sao Tome and Principe
Nigeria
Philippines
Senegal
Pakistan
Rwanda
Sierra Leone
Proportion of ANCS
Year
0
25
50
75
100
2000
2010
2020
2030
Urban
Rural
National

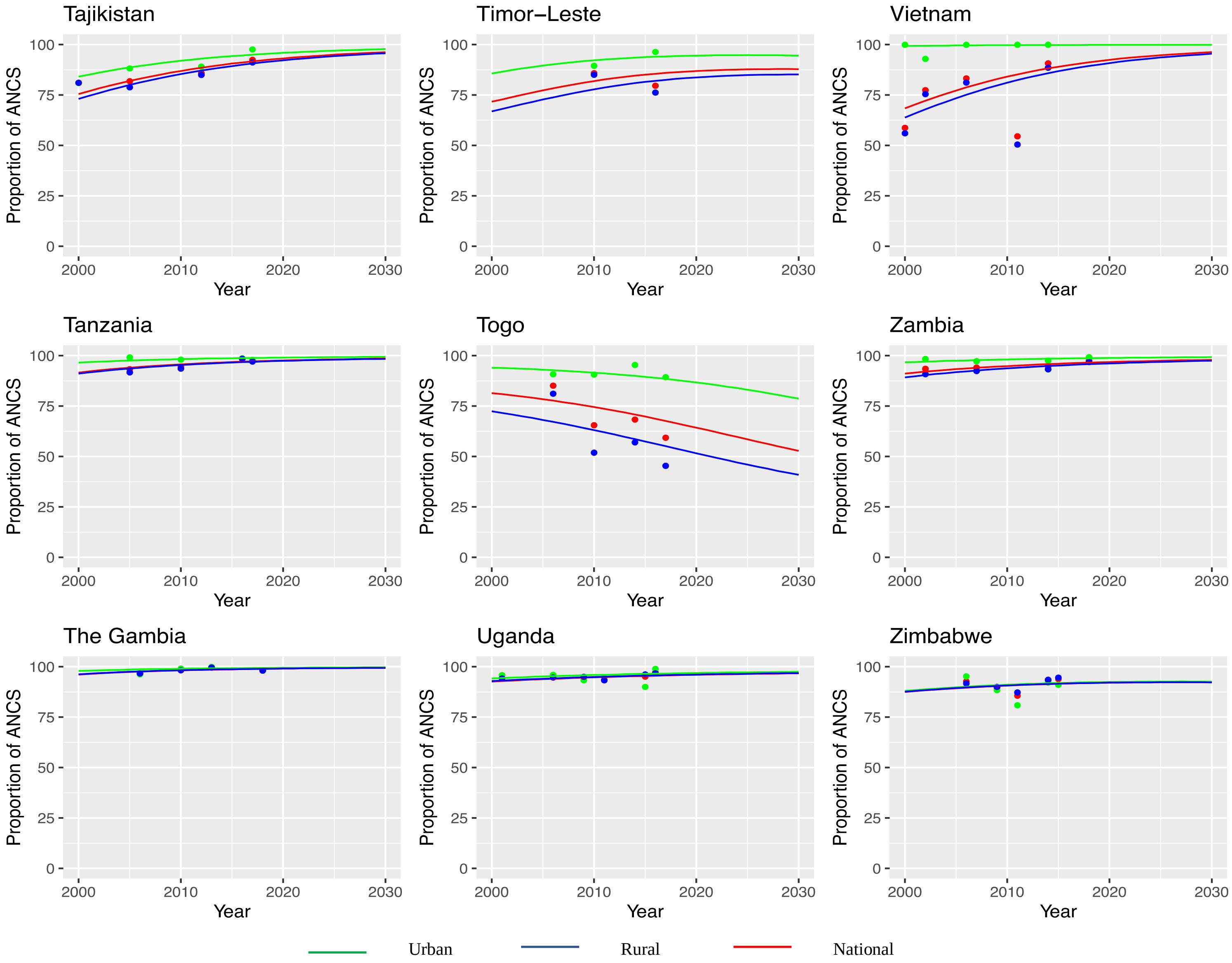
Tajikistan
Timor−Leste
Vietnam
Tanzania
Togo
Zambia
The Gambia
Uganda
Zimbabwe
Proportion of ANCS
Year
0
25
50
75
100
2000
2010
2020
2030
Urban
Rural
National

Figure S3: Detail years-specific observed and predicted coverage of four ANC visits by area of residence (national, urban, and rural)

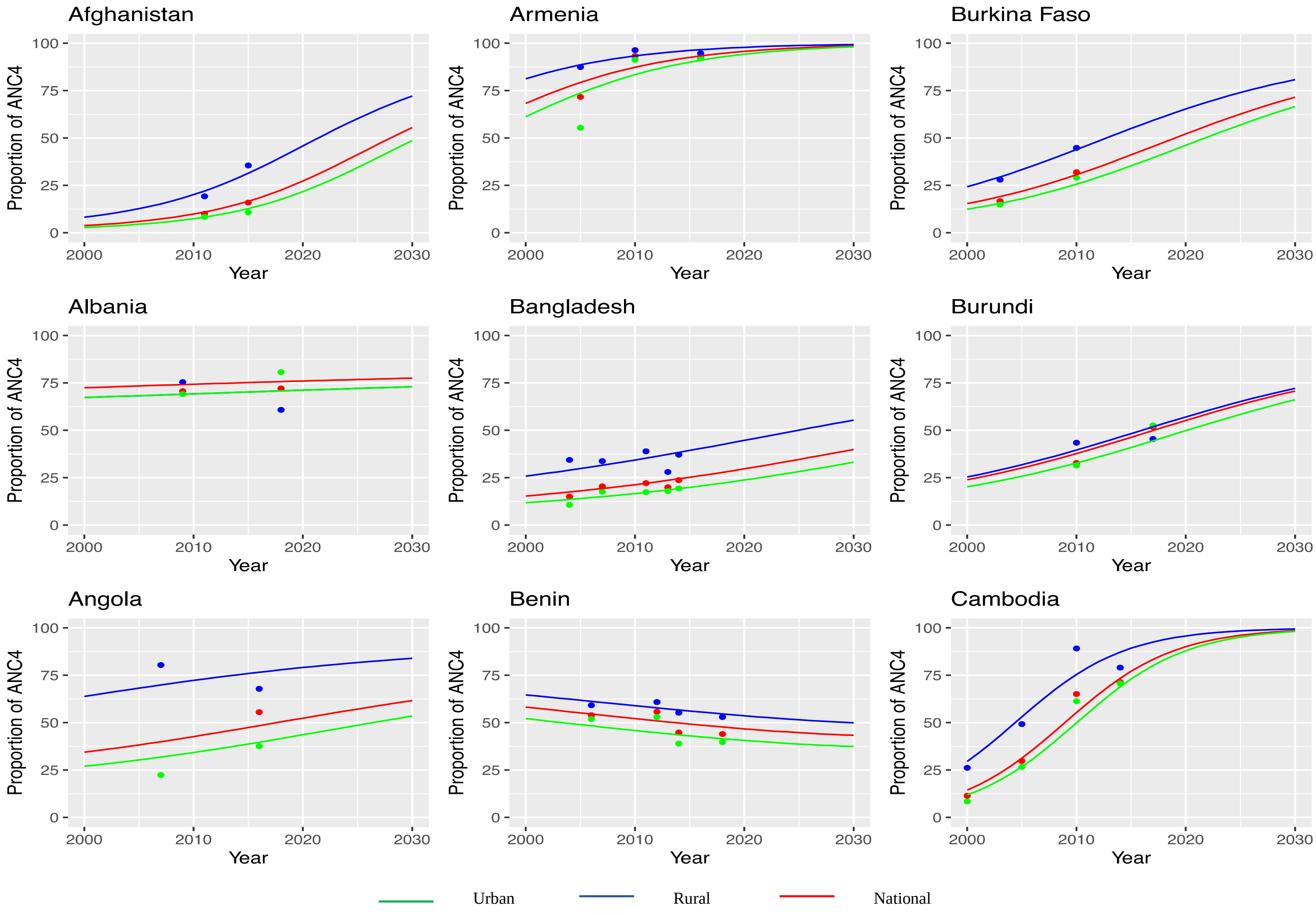

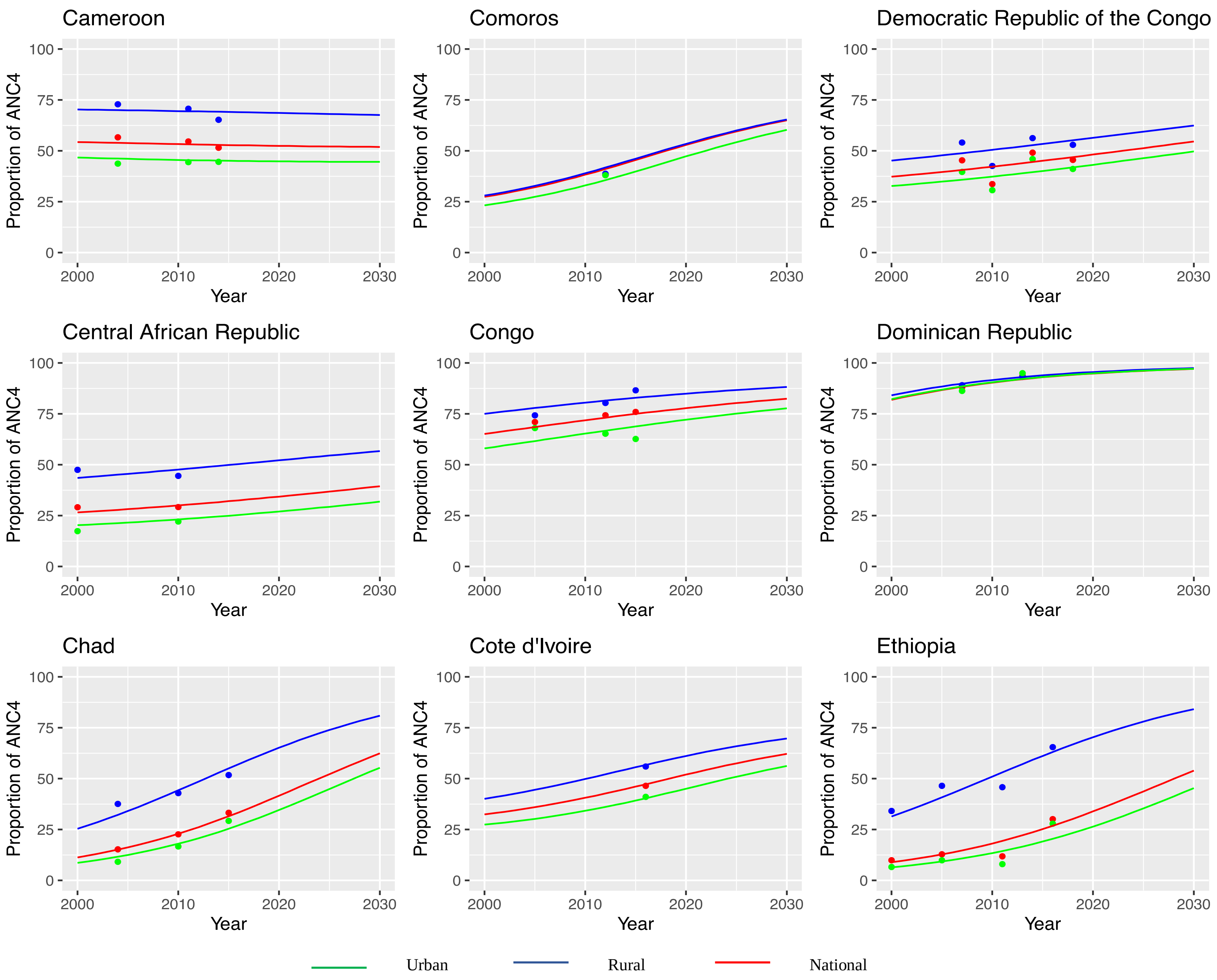

Cameroon
Comoros
Democratic Republic of the Congo
Central African Republic
Congo
Dominican Republic
Chad
Cote d'Ivoire
Ethiopia
Proportion of ANC4
Year
0
25
50
75
100
2000
2010
2020
2030
Urban
Rural
National

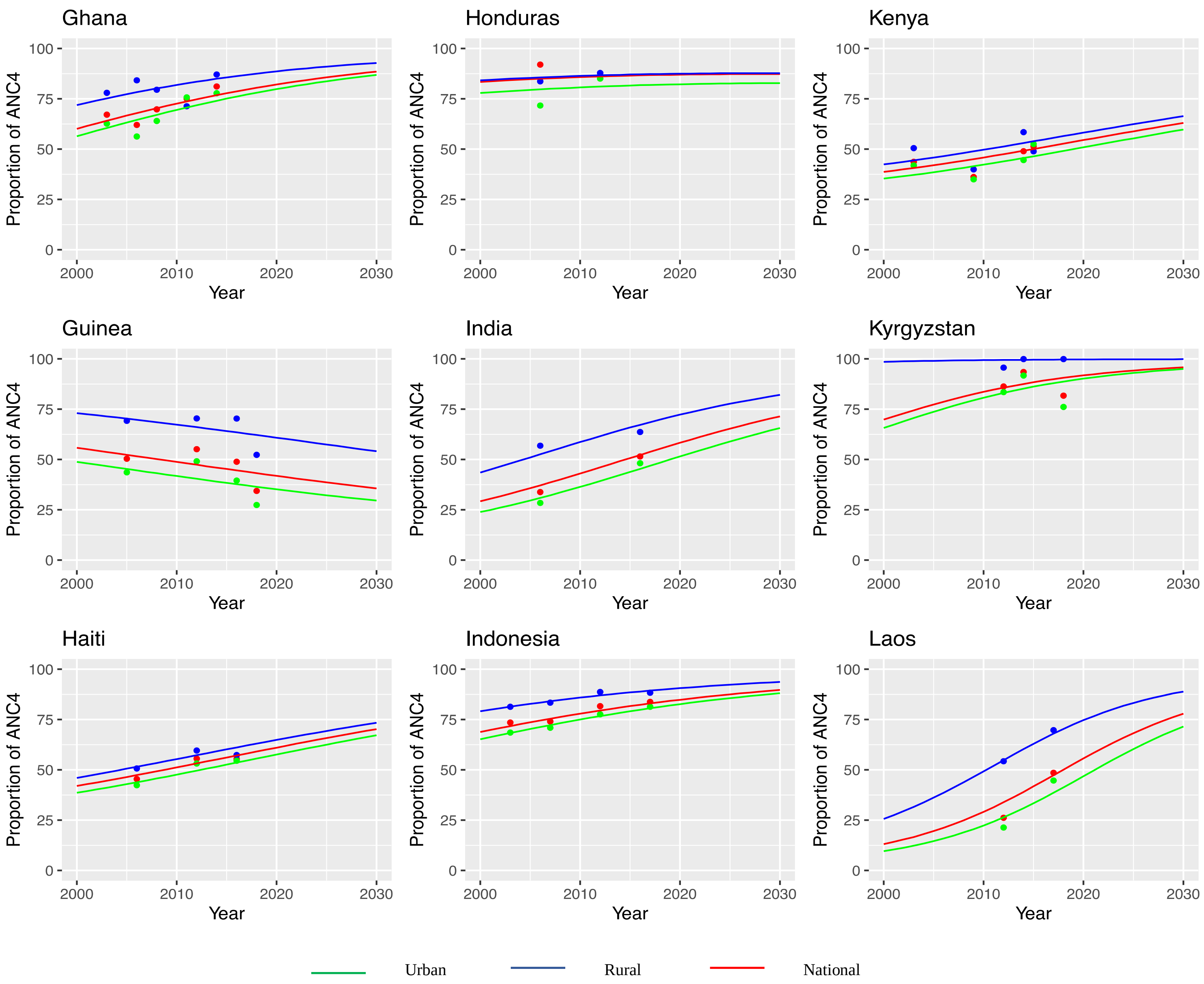
Ghana
Honduras
Kenya
Guinea
India
Kyrgyzstan
Haiti
Indonesia
Laos
Proportion of ANC4
Year
0
25
50
75
100
2000
2010
2020
2030
Urban
Rural
National

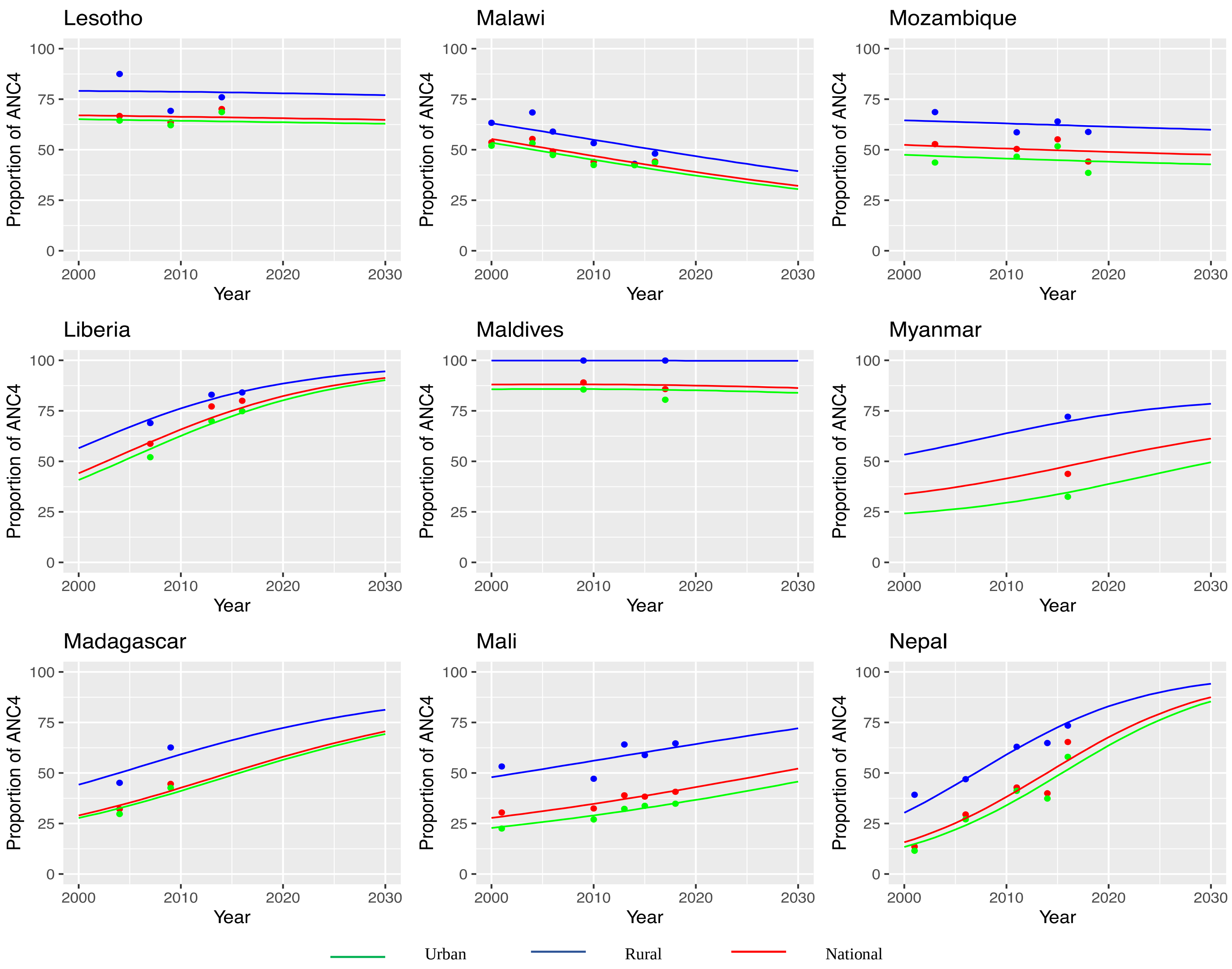
Lesotho
Malawi
Mozambique
Liberia
Maldives
Myanmar
Madagascar
Mali
Nepal
Proportion of ANC4
Year
0
25
50
75
100
2000
2010
2020
2030
Urban
Rural
National

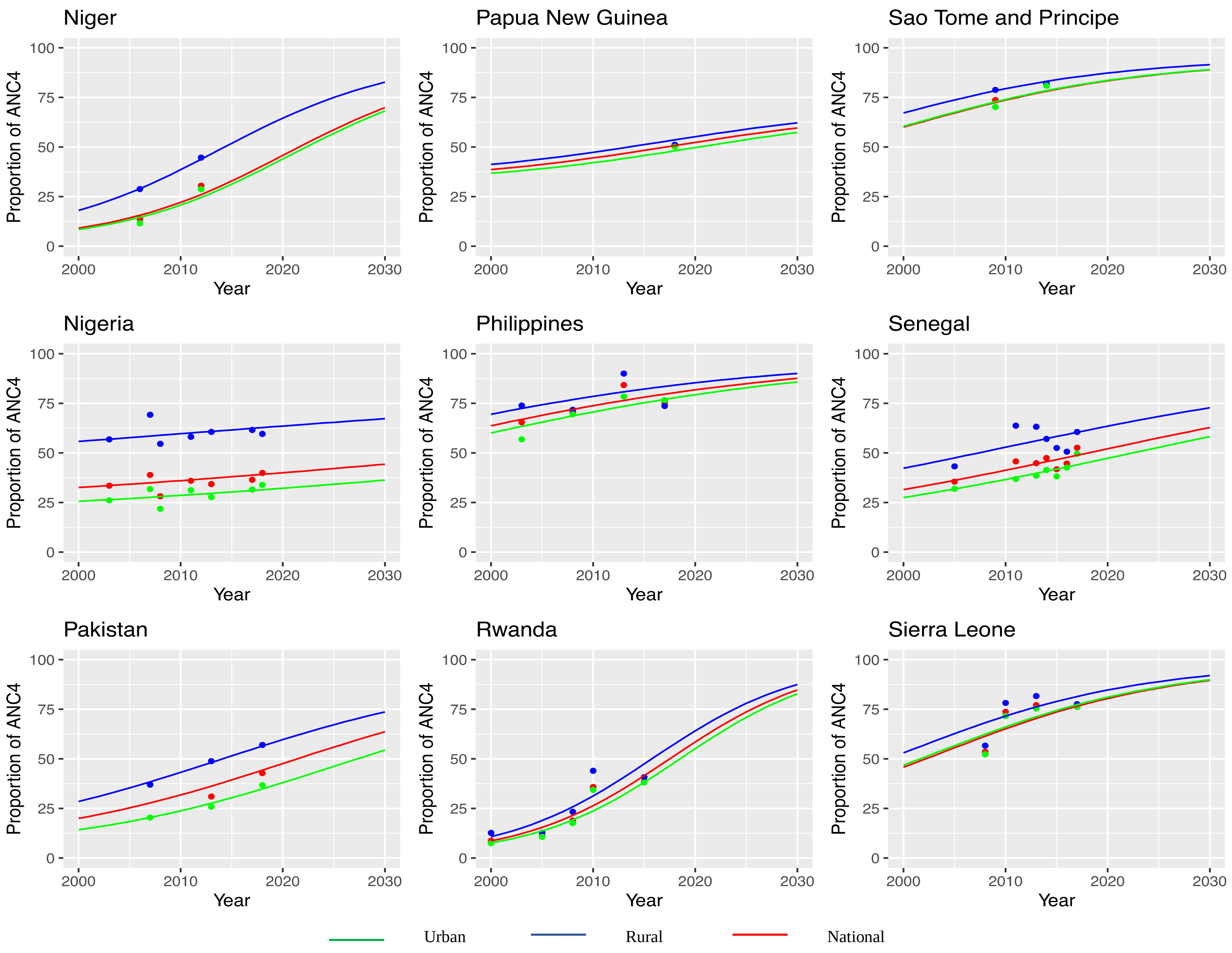

Niger
Papua New Guinea
Sao Tome and Principe
Nigeria
Philippines
Senegal
Pakistan
Rwanda
Sierra Leone
Proportion of ANC4
Year
0
25
50
75
100
2000
2010
2020
2030
Urban
Rural
National

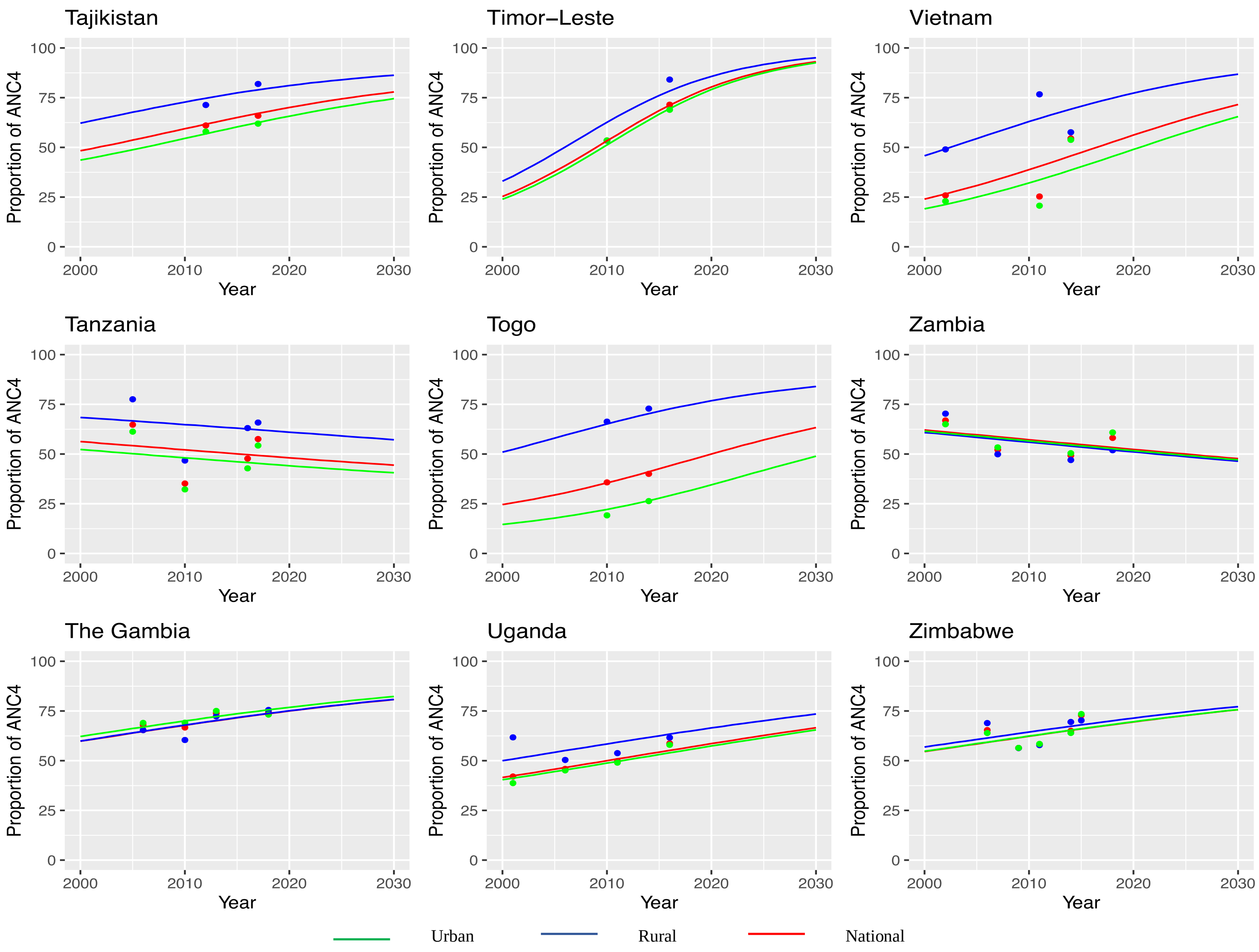
Tajikistan
Timor−Leste
Vietnam
Tanzania
Togo
Zambia
The Gambia
Uganda
Zimbabwe
Proportion of ANC4
Year
0
25
50
75
100
2000
2010
2020
2030
Urban
Rural
National
